# An Index for Characterization of Natural and Non-Natural Amino Acids for Peptidomimetics

**DOI:** 10.1371/journal.pone.0067844

**Published:** 2013-07-23

**Authors:** Guizhao Liang, Yonglan Liu, Bozhi Shi, Jun Zhao, Jie Zheng

**Affiliations:** 1 Key Laboratory of Biorheological Science and Technology (Chongqing University), Ministry of Education, Bioengineering College Chongqing University, Chongqing, China; 2 Department of Chemical and Biomolecular Engineering, The University of Akron, Akron, Ohio, United States of America; Russian Academy of Sciences, Institute for Biological Instrumentation, Russian Federation

## Abstract

Bioactive peptides and peptidomimetics play a pivotal role in the regulation of many biological processes such as cellular apoptosis, host defense, and biomineralization. In this work, we develop a novel structural matrix, Index of Natural and Non-natural Amino Acids (NNAAIndex), to systematically characterize a total of 155 physiochemical properties of 22 natural and 593 non-natural amino acids, followed by clustering the structural matrix into 6 representative property patterns including geometric characteristics, H-bond, connectivity, accessible surface area, integy moments index, and volume and shape. As a proof-of-principle, the NNAAIndex, combined with partial least squares regression or linear discriminant analysis, is used to develop different QSAR models for the design of new peptidomimetics using three different peptide datasets, i.e., 48 bitter-tasting dipeptides, 58 angiotensin-converting enzyme inhibitors, and 20 inorganic-binding peptides. A comparative analysis with other QSAR techniques demonstrates that the NNAAIndex method offers a stable and predictive modeling technique for *in silico* large-scale design of natural and non-natural peptides with desirable bioactivities for a wide range of applications.

## Introduction

Naturally occurring bioactive peptides such as amyloid peptides, antimicrobial peptides, cell penetration peptides, and fusion peptides play various biological roles (e.g. hormones, enzyme substrates and inhibitors, neurotransmitters, drugs and antibiotics, and self-assembly building blocks) in regulating various biological processes and metabolisms [1]–[3]. Due to peptidic nature, most of these native peptides suffer from poor bioavailability and poor proteolytic stability, which greatly limit their in vitro and in vivo applications. To address these limitations, using the existing peptides as structural templates and high-throughput screening approaches together with combinatorial library and analogue chemistry synthesis have been widely used to brute-force search and systematically design new stable and active peptide mimetics [4]. Such approaches enable (i) to explore a vast population of diverse chemical and biochemical sequences from other protein/peptide families to increase sequence diversity and (ii) to introduce non-natural, D-amino acids, or β-amino acids to improve proteolytic stability [5], [6]. The obtained potent peptide mimetics usually have similar backbone structures to their original peptide templates, but with key functional residues being modified for improving biological or physiochemical properties, metabolic stability, and sequence diversity and accessibility [7].

Cell-phage and mirror-phage approaches in combination with mutationgenetics are powerful high-throughput techniques to screen and identify active peptides and to construct combinatorial synthetic peptide libraries. These approaches have produced a number of FDA-approved peptide-based drugs including ACE inhibitors, HIV protease inhibitors, and cancer immunotherapeutics [3], [8]. Another common structural-assisted design approach lies in the replacement of individual amino acids with non-natural amino acids or specific structural motifs to iteratively optimize designs [7],[9]. The inclusion of the non-natural amino acids (e.g. isosteric replacements, cyclic peptide derivatives, and bond surrogates) [10] and/or the specific structural motifs (e.g. β-turn, helices, and β-sheets) [11] in the first-generation mimetics is expected to induce conformational changes of backbones and/or side chains, and thus to yield favorable bindings to targets. As the design process continuously proceeds to next generations, amine variants, side chain lengths, and conformational constraints can be further optimized to achieve desirable activity. However, given a large number of undetermined compounds and the limited synthesis/purification/characterization ability by experiments, it is almost infeasible to conduct a large-scale search for both sequences and structures in a complete sequence space [12]. In addition, such brute-force and high-cost screening methods would be tedious, prone to experimental errors, and require tremendous expense. More importantly, these experimental screening approaches provide little structural and binding information of designed peptides, which often lead to irrational design and many inactive compounds.

Complement to experimental screening approaches, computational virtual screening methods including quantitative structure-activity relationship (QSAR) and molecular docking provide valuable alternatives for rapidly screening and selecting potent compounds. More importantly, computational screening methods strive to illustrate structural, dynamic, and binding information at an atomic level, making it necessary for the better understanding of sequence-structure-activity relationship and design principles for peptides mimetics. The QSAR is currently an important contributor to rational design of drugs, materials, catalysts, and proteins/peptides with desirable activities and functions [13]–[17]. The underlying hypothetical principle of QSAR models is to define mathematical relationships between a set of molecular descriptors and a given activity (chemical, physical, or biological activity) as an end point, to predict the activity of unknown ligands [18]–[29]. In the past decades, a number of 2D-QSAR models for peptides have been developed [14], [22], [24], [30]–[32]. Most of the 2D-QSAR models use local descriptors, e.g. structural and property parameters, orthogonal binary codes, and principal properties, etc., to sequentially characterize the sequence and 2D chemical structure, of peptides based on their amino acid compositions and positions. Principal component analysis and factor analysis are often used to extract useful structural information from the original parameter matrix of amino acids. As actual spatial structural features (e.g. isotropic surface area, solvent-accessible surface area, electronic charge index, secondary structural population, etc.) of the ligands are well characterized [20], [33], the 3D-QSAR methods (e.g. CoMFA and CoMSIA) [34]–[36] generally have a better prediction in ligand design than the 2D-QSAR methods. However, in most cases, 3D atomic structures of the ligands in the dataset are often not available experimentally; thus structural homology modeling is required to predict 3D structures for conformational alignment and structural parameterization. Due to the lack of reliable 3D structures of ligands or misrepresented bioactive conformers, several studies have reported that 2D descriptors, if appropriately selected, are actually superior to 3D ones [37]–[39]. Additionally, high-dimensional QSAR models usually require more structural information to construct the structure-activity relationship, e.g. the conformational profile of ligands for 4D-QSAR [40], the receptor-induced fit mode for 5D-QSAR [41], and solvation model for 6D-QSAR [42]. As compared with those high-dimensional QSAR models, the 2D-QSAR descriptors could be more amenable to interpret some key structural features of small ligands without requiring more undetermined conformations.

Rapid development and considerable progress have been made to develop different QSAR models to produce more active peptides and to better understand the mechanism of their actions. However, most of the existing QSAR methods for peptide design focus on the characterization of natural amino acids, but pay less attention to non-natural amino acids [31], [32]. On one hand, inadequate data of non-natural amino acids provide rather limited selection for structural modification and render the uncertainty of predictive performance. On the other hand, recent progress enables to simultaneously encode hundreds of different non-natural sidechains in the same organism [43]. Another important progress is that the recently constructed SwissSidechain database [44] contains molecular and structural data for 210 non-natural alpha amino acid sidechains, both in L- and D-conformations. Yet it still remains a very challenging task to accurately describe non-natural amino acids by QSAR models to date.

In our previous work [24], we have developed a new 2D-QSAR index of factor analysis scales of generalized amino acid information (FASGAI) to characterize natural peptides. The FASGAI method clusters 335 physicochemical properties of each of 20 natural amino acids into 6 factors of hydrophobicity, alpha and turn propensities, bulky properties, compositional characteristics, local flexibility, and electronic properties, which can be generally used to characterize any given peptide. The FASGAI method has successfully predicted the activity of a variety of peptides, including the inhibitory activity of HIV type 1 protease [45], the binding affinity between the human amphiphysin-1 SH3 domains and designed ligands [46] and between MHC class I and binding peptides [14]. To continue developing peptide mimetics, here we developed a novel structural matrix, Index of Natural and Non-natural Amino Acids (NNAAIndex), to systematically characterize a total of 155 physiochemical properties of 22 natural and 593 non-natural amino acids. We then developed different QSAR models to design peptide mimics for three different classes of bitter-tasting dipeptide (BTD), angiotensin-converting enzyme (ACE) inhibitors, and inorganic-binding peptides with a specific activity in silico.

## Materials and Methods

### NNAAIndex Characterization

We first computed a total of 384 physicochemical properties of 22 natural amino acids and 593 non-natural amino acids (http://www.sigmaaldrich.com/chemistry/) using E-dragon [47] and MOE programs [48]. Then we applied tentative exploratory factor analysis to quantify the perceived structural features, which guide to identify 155 out of 384 properties, involving topological descriptors, physical properties, subdivided surface areas, Kier&Hall connectivity and Kappa shape indices, pharmacophore features, partial charge, surface area, volume and shape, conformation dependent charge, geometrical descriptors, etc., to characterize the key structural features of 615 amino acids. Among 155 properties, some of them may be coupled or inter-correlated. Thus, to improve the data interpretability, principal component method with a Kaiser normalized promax algorithm was used to identify and cluster a subset of numerical variables to map out the entire constellation of 155 highly intercorrelated physiochemical properties. The factor analysis method produced 6 new factor scores, which accounted for ∼82.70% variance of 155 variables based on the relationship between component number and eigenvalues. The 6 factor vectors, named as NNAAIndex, account for most of structural information of the 155 properties, so they can be used to represent the structural features of peptides. Since each natural or non-natural amino acid is represented by 6 NNAAIndex factors, the sequence and structural features of any peptide can be characterized by simply constructing a 6×*n* NNAAIndex matrix, where *n* is the number of amino acids.

### Structural Feature Selection

A genetic arithmetic-partial least square method (GA-PLS) [49] was used to select the variables related to their structural attributions of the peptides studied. In the GA-PLS, the chromosome and its fitness in the species corresponded to a set of variables and the internal prediction ability of the derived PLS model, respectively. The fitness of each chromosome is evaluated by the internal prediction ability of the PLS model derived from a binary bit pattern. The internal predictive performance of the model is expressed by a cross validation square of cumulative multiple correlation coefficient (*R* ^2^) value (denoted by *Q* ^2^) and validated by the leave-one-out (LOO) procedure as follows:
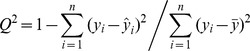
(1)where *y_i_* and 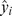 represents the observed value and the predicted value of the dependent variable, respectively; 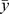 is the mean observed value of the dependent variable; *n* is the total number of samples. The empirical parameters in the GA-PLS method were set as follows: the number of populations was 200, the maximum number of generations was 200, the generation gap was 0.8, the crossover frequency was 0.5, and the mutation rate was 0.005.

### Partial Least Squares (PLS)

To validate the predictive statistical model, PLS regression was used to correlate variations in the biological activities with variations in the respective descriptors for a given data set. The PLS is particularly well suited to analyze biological data because the algorithm can handle noisy or collinear signals [50]–[53]. In PLS regression, a matrix of independent variables was regressed against a dependent matrix as described below. The optimal number of principal components (PCs), corresponding to the smallest standard error of prediction, was determined by the LOO cross-validation procedure, which yields a cross-validated Q^2^ to measure the predictivity of the PLS model. Using the optimal number of PCs, the final PLS analysis was carried out without cross-validation to generate a predictive QSAR model with a conventional R^2^. The PLS regression algorithm consists of an outer relation (X and Y block individually) and an inner relation linking both X and Y blocks:
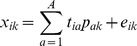
(2)

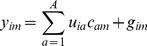
(3)where the *t* and *u* latent variables are correlated through the inner relation given below, which leads to the estimation of the *y* from the *x*.
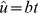
(4)


### Linear Discriminant Analysis (LDA)

LDA realizes the process that how objects will be classified according to many observed samples. The basic theory of LDA is to classify the dependent by dividing an n-dimensional descriptor space into two regions (two classes), which are separated by a hyperplane defined by a linear discriminant function as follows: Y = a_0_+a_1_X_1_+a_2_X_2_+…+a_n_X_n_, where Y is the dependent variable, X_1_, X_2_, …X_n_ represents the independent variable (observed values), a_1_, a_2_,…a_n_ corresponds to weights associated with the respective independent variable, which is discriminant coefficients. Independent variable space is divided into two regions through the hyperplane, then to discriminate which region each compound belongs to. As inputs of the LDA model, the variables are chosen according to F value of partial F test, i.e. the variable is accepted by the model when F value is greater than 3.84, but rejected when F value is less than 2.71.

## Results and Discussion

### Physicochemical Representation of NNAAIndex Model

It is generally accepted for the Anfinsen's dogma that the native structures and biological functions of peptides or proteins are determined by their primary amino acid sequences [54]. Here, we first selected a total of 155 properties, including topological descriptors, physical properties, subdivided surface areas, Kier and Hall connectivity and Kappa shape indices, pharmacophore features, partial charge, surface area, volume and shape, conformation dependent charge, geometrical descriptors, etc., to characterize the structural features of 615 non-natural amino acids. Considering that a large matrix containing much redundant information is not suitable for characterizing the structural and sequence properties of peptides and proteins, we used the promax algorithm with Kaiser normalization to further cluster 155 variables into 6 independent factors. The 6 factor scores (Table S1) accounted for ∼82.70% structural information of 155 variables.

To explore the physicochemical meaning of 6 factors, we summarized the loading and communality of 155 variables in Table 1. The first factor is designated as a geometric index. The parameters related to 3D-Wiener index, Wiener-type index from electronegativity weighted distance matrix, and Z weighted distance matrix, hyper-detour index, Gutman molecular topological index, centralization, D/D index, Schultz molecular topological index, Wiener W index, etc., produce positive loading on the first factor; while the parameters, concerned with log of the aqueous solubility, folding degree index, mass density, total structure connectivity index, spherosity, Balaban-type index from electronegativity and mass weighted distance matrix, etc., have negative loading on this factor.

**Table 1:** Loading and communality of 155 variables on 6 factors.

No.	Property	Factor 1	Factor 2	Factor 3	Factor 4	Factor 5	Factor 6	Communality
1	First Zagreb index M1	0.798	0.018	0.127	−0.015	−0.004	−0.146	0.992
2	Second Zagreb index M2	0.776	0.010	0.156	−0.008	−0.010	−0.211	0.989
3	Quadratic index	0.582	−0.060	0.294	−0.013	0.002	−0.409	0.949
4	Narumi simple topological index (log)	0.774	0.066	0.255	−0.011	−0.017	−0.072	0.991
5	Narumi harmonic topological index	0.204	0.089	0.787	−0.020	−0.016	0.001	0.918
6	Narumi geometric topological index	0.165	0.056	0.759	−0.029	0.004	−0.072	0.929
7	Total structure connectivity index	−0.347	−0.038	−0.297	0.059	−0.010	−0.059	0.936
8	Pogliani index	0.822	0.068	0.025	−0.014	−0.004	−0.022	0.992
9	Ramification index	0.658	−0.082	−0.111	−0.018	0.027	−0.352	0.932
10	Polarity number	0.794	−0.021	0.027	−0.003	−0.031	−0.239	0.978
11	Log of product of row sums (PRS)	0.904	0.046	0.058	−0.006	−0.003	−0.010	0.997
12	Average vertex distance degree	0.991	0.042	0.067	−0.002	0.018	0.072	0.996
13	Mean square distance index (Balaban)	−0.204	−0.043	−0.059	0.074	−0.041	0.761	0.946
14	Schultz molecular topological index (mti)	1.119	0.027	0.093	0.015	0.015	−0.086	0.990
15	Gutman molecular topological index	1.122	0.034	0.115	0.017	0.008	−0.096	0.989
16	Xu index	0.782	0.053	0.059	−0.023	−0.001	0.075	0.995
17	Superpendentic index	0.799	−0.051	−0.265	−0.012	0.027	0.036	0.975
18	Wiener W index	1.118	0.021	0.066	0.014	0.022	−0.073	0.989
19	Mean Wiener index	0.768	0.053	0.044	−0.020	0.017	0.392	0.988
20	Harary H index	0.835	0.032	0.102	−0.007	−0.017	−0.135	0.993
21	Quasi-Wiener index (Kirchhoff number)	1.110	0.037	0.038	0.007	0.038	−0.062	0.983
22	Detour index	1.094	−0.020	0.157	0.027	−0.012	−0.141	0.978
23	Hyper-detour index	1.122	−0.055	0.191	0.030	−0.014	−0.126	0.929
24	Reciprocal hyper-detour index	0.834	0.122	−0.330	−0.013	0.033	0.069	0.974
25	Distance/detour index	1.028	0.077	−0.063	0.000	0.023	−0.050	0.975
26	All-path Wiener index	0.985	−0.041	0.245	0.051	−0.065	−0.157	0.844
27	Wiener-type index from Z weighted distance matrix (Barysz matrix)	1.129	0.015	0.064	0.008	0.019	−0.056	0.986
28	Wiener-type index from van der Waals weighted distance matrix	1.105	0.039	0.048	0.003	0.043	−0.056	0.986
29	Wiener-type index from electronegativity weighted distance matrix	1.131	0.019	0.060	0.006	0.026	−0.070	0.986
30	Wiener-type index from polarizability weighted distance matrix	1.098	0.040	0.045	0.005	0.041	−0.046	0.984
31	Balaban distance connectivity index	−0.127	0.053	−0.925	−0.008	0.000	−0.146	0.968
32	Balaban-type index from mass weighted distance matrix	−0.243	0.089	−0.960	−0.028	0.056	−0.158	0.905
33	Balaban-type index from electronegativity weighted distance matrix	−0.277	0.027	−0.985	−0.032	0.008	−0.146	0.943
34	Maximal electrotopological positive variation	0.626	−0.052	0.038	−0.103	0.046	−0.202	0.812
35	Molecular electrotopological variation	0.557	0.078	−0.153	−0.020	0.028	−0.045	0.947
36	E-state topological parameter	0.827	0.015	−0.240	−0.003	0.091	−0.092	0.854
37	Kier symmetry index	0.829	0.017	0.101	−0.023	0.014	0.037	0.972
38	1-path Kier alpha-modified shape index	0.892	0.046	−0.192	−0.018	0.002	0.054	0.997
39	2-path Kier alpha-modified shape index	0.840	0.106	−0.131	0.000	−0.036	0.337	0.959
40	3-path Kier alpha-modified shape index	0.573	0.041	−0.397	−0.015	−0.059	0.532	0.899
41	Kier flexibility index	0.785	0.086	−0.296	0.002	−0.038	0.388	0.955
42	Path/walk 5 - Randic shape index	0.027	0.040	0.341	−0.078	−0.115	−0.176	0.795
43	Eccentric connectivity index	0.988	0.022	0.145	−0.003	0.013	0.125	0.991
44	Eccentricity	0.987	0.017	0.101	−0.003	0.018	0.154	0.991
45	Average eccentricity	0.738	0.006	0.116	−0.016	0.011	0.500	0.981
46	Eccentric	0.649	−0.092	0.137	0.006	−0.015	0.711	0.914
47	Mean distance degree deviation	1.017	0.008	0.051	0.011	−0.002	0.096	0.989
48	Unipolarity	0.965	0.028	0.086	−0.007	0.027	0.156	0.989
49	Centralization	1.122	0.038	0.037	0.023	0.005	−0.189	0.970
50	Variation	1.051	−0.008	0.063	0.022	−0.027	0.011	0.977
51	Balaban centric index	0.628	−0.105	−0.659	0.009	0.046	−0.066	0.930
52	Lopping centric index	0.203	0.050	−0.122	−0.031	0.151	0.727	0.817
53	Radial centric information index	0.544	−0.035	0.176	−0.012	−0.030	0.627	0.934
54	Unsaturation index	0.102	0.034	0.237	−0.079	0.065	0.082	0.893
55	Hydrophilic factor	0.022	0.352	−0.186	−0.172	−0.103	0.093	0.874
56	Ghose-Crippen molar refractivity	0.854	−0.006	0.038	−0.023	−0.013	−0.043	0.991
57	Moriguchi octanol-water partition coeff. (logp)	0.072	−0.263	0.068	−0.056	−0.137	−0.266	0.887
58	Ghose-Crippen octanol-water partition coeff. (logp)	0.498	−0.496	−0.058	−0.027	0.069	0.047	0.910
59	Verhaar model of Fish base-line toxicity from MLOGP	−0.188	0.195	−0.062	0.070	0.114	0.334	0.859
60	Sum of the atomic polarizabilities (including implicit hydrogens) with polarizabilities.	0.929	−0.032	0.022	−0.028	−0.017	−0.036	0.992
61	Sum of the absolute value of the difference between atomic polarizabilities of all bonded atoms in the molecule (including implicit hydrogens) with polarizabilities.	0.937	−0.041	−0.103	−0.049	−0.005	0.007	0.911
62	Total charge of the molecule (sum of formal charges).	0.044	−0.050	0.066	0.017	0.048	−0.006	0.938
63	Molecular refractivity (including implicit hydrogens). This property is an atomic contribution model	0.852	0.003	0.035	−0.008	−0.011	−0.051	0.991
64	Molecular weight (including implicit hydrogens) in atomic mass units with atomic weights.	0.765	0.103	−0.019	−0.001	0.007	−0.090	0.962
65	Log of the octanol/water partition coefficient (including implicit hydrogens)	0.313	−0.440	−0.017	−0.077	−0.289	−0.080	0.829
66	Log of the aqueous solubility (mol/L).	−0.564	0.208	0.077	−0.073	−0.083	0.062	0.920
67	Log of the octanol/water partition coefficient (including implicit hydrogens).	0.481	−0.573	−0.048	0.058	0.007	0.091	0.901
68	Polar surface area (Å^2^) calculated using group contributions to approximate the polar surface area from connection table information only	0.282	0.488	−0.165	0.089	0.003	0.025	0.930
69	Van der Waals volume (Å^3^) calculated using a connection table approximation.	0.903	−0.017	0.020	−0.031	−0.012	−0.025	0.996
70	Area of van der Waals surface (Å^2^) calculated using a connection table approximation.	0.895	0.033	−0.119	−0.021	0.001	0.005	0.993
71	Atomic connectivity index (order 0). This is calculated as the sum of 1/sqrt(*d_i_*) over all heavy atoms *i* with *d_i_*>0.	0.850	0.035	−0.038	−0.016	0.000	−0.028	0.996
72	Atomic valence connectivity index (order 0). This is calculated as the sum of 1/sqrt(*v_i_*) over all heavy atoms *i* with *v_i_*>0.	0.887	−0.003	−0.044	−0.009	0.004	−0.078	0.988
73	First kappa shape index: (*n*-1)^2^/*m* ^2^.	0.867	0.051	−0.145	−0.015	−0.001	0.055	0.995
74	First alpha modified shape index: *s* (*s*-1)^2^/*m* ^2^ where *s* = *n*+*a*.	0.881	0.046	−0.241	−0.003	0.025	0.019	0.969
75	Kier molecular flexibility index: (kiera1) (kiera2)/*n*.	0.705	0.073	−0.343	0.020	−0.015	0.313	0.907
76	Balaban's connectivity topological index	−0.136	0.051	−0.926	−0.009	0.000	−0.147	0.969
77	Number of hydrogen bond acceptor atoms	0.373	0.627	0.039	−0.068	−0.050	−0.073	0.845
78	Number of acidic atoms.	0.021	−0.039	0.049	−0.004	0.037	−0.011	0.949
79	Number of hydrogen bond donor atoms	0.240	0.627	0.001	−0.237	−0.145	−0.112	0.810
80	Number of hydrophobic atoms	0.806	−0.252	0.078	−0.026	−0.020	−0.007	0.980
81	Approximation to the sum of VDW surface areas of acidic atoms	0.022	−0.039	0.051	0.002	0.039	−0.018	0.945
82	Approximation to the sum of VDW surface areas of pure hydrogen bond donors	−0.085	−0.027	−0.182	−0.211	−0.160	−0.010	0.758
83	Approximation to the sum of VDW surface areas of hydrophobic atoms	0.869	−0.240	−0.083	−0.053	−0.014	0.013	0.974
84	Approximation to the sum of VDW surface areas (Å^2^) of atoms typed as “other”.	0.154	0.461	−0.072	0.308	0.116	−0.064	0.726
85	Approximation to the sum of VDW surface areas (Å^2^) of polar atoms (atoms that are both hydrogen bond donors and acceptors), such as -OH.	0.287	0.486	−0.092	−0.112	−0.016	0.017	0.915
86	Value of the potential energy	0.259	0.070	−0.099	0.134	0.011	−0.193	0.869
87	Angle bend potential energy	0.185	−0.048	−0.140	0.106	−0.107	−0.072	0.713
88	Value of the potential energy with all bonded terms disabled	0.337	0.066	−0.085	0.103	0.032	−0.155	0.823
89	Solvation energy	−0.093	−0.198	−0.131	0.008	0.028	0.033	0.836
90	Bond stretch-bend cross-term potential energy	0.187	0.088	0.057	−0.200	0.009	−0.103	0.859
91	Local strain energy	0.183	−0.021	−0.032	0.150	−0.022	0.024	0.811
92	Van der Waals component of the potential energy	0.579	−0.147	−0.062	0.003	−0.094	−0.118	0.889
93	Water accessible surface area calculated using a radius of 1.4 A for the water molecule. A polyhedral representation is used for each atom in calculating the surface area.	0.820	0.060	−0.005	−0.026	0.018	0.146	0.987
94	Mass density: molecular weight divided by van der Waals volume as calculated in the vol descriptor.	−0.349	0.338	−0.061	0.035	0.027	−0.181	0.819
95	Van der Waals volume calculated using a grid approximation (spacing 0.75 A).	0.915	−0.005	−0.004	−0.024	−0.002	−0.019	0.996
96	Van der Waals surface area. A polyhedral representation is used for each atom in calculating the surface area.	0.906	0.013	−0.033	−0.021	0.008	0.050	0.995
97	Amphiphilic moment	0.077	−0.119	−0.048	0.013	0.897	0.069	0.939
98	Hydrophobic volume (8 descriptors)	0.559	−0.008	0.059	0.022	0.090	−0.010	0.974
99	Hydrophobic volume (8 descriptors)	0.569	−0.023	0.052	0.031	0.065	0.004	0.980
100	Hydrophobic volume (8 descriptors)	0.605	−0.054	0.018	0.031	0.076	−0.030	0.983
101	Hydrophobic volume (8 descriptors)	0.604	−0.053	0.016	0.019	0.104	−0.051	0.985
102	Hydrophobic volume (8 descriptors)	0.578	−0.028	0.035	0.012	0.132	−0.056	0.985
103	Hydrophobic volume (8 descriptors)	0.521	0.030	0.127	0.014	0.128	−0.034	0.973
104	Hydrophobic volume (8 descriptors)	0.390	0.046	0.161	0.031	0.025	0.118	0.942
105	Hydrophobic volume (8 descriptors)	0.245	0.063	0.070	0.083	−0.046	0.025	0.900
106	Lowest hydrophobic energy (3 descriptors)	−0.150	0.021	−0.051	−0.149	0.023	0.216	0.853
107	Lowest hydrophobic energy (3 descriptors)	−0.129	0.030	−0.050	−0.153	0.020	0.216	0.864
108	Lowest hydrophobic energy (3 descriptors)	−0.107	0.004	−0.049	−0.151	0.012	0.213	0.868
109	Surface globularity	0.582	0.223	−0.024	−0.015	0.021	0.411	0.901
110	H-bond donor capacity (8 descriptors)	0.283	0.518	0.154	0.209	−0.153	0.296	0.843
111	H-bond donor capacity (8 descriptors)	0.338	0.340	0.116	0.186	−0.185	0.347	0.922
112	H-bond donor capacity (8 descriptors)	0.302	0.456	0.033	0.140	−0.096	0.213	0.957
113	H-bond donor capacity (8 descriptors)	0.208	0.786	0.016	0.081	0.020	−0.004	0.973
114	H-bond donor capacity (8 descriptors)	0.075	1.067	0.021	0.017	0.085	−0.169	0.967
115	H-bond donor capacity (8 descriptors)	−0.002	1.205	−0.007	−0.039	0.141	−0.316	0.940
116	Hydrophilic-Lipophilic (2 descriptors)	−0.223	0.808	−0.057	−0.042	−0.117	−0.102	0.883
117	Hydrophilic-Lipophilic (2 descriptors)	−0.237	0.909	−0.071	−0.058	−0.082	−0.175	0.844
118	Hydrophobic integy moment (8 descriptors)	−0.123	0.224	0.018	−0.019	0.928	0.137	0.895
119	Hydrophobic integy moment (8 descriptors)	−0.065	0.194	0.012	−0.083	0.889	0.136	0.866
120	Hydrophobic integy moment (8 descriptors)	−0.060	0.367	0.052	−0.054	0.514	−0.005	0.730
121	Hydrophilic integy moment (8 descriptors)	0.153	−0.080	−0.026	0.035	0.829	−0.058	0.903
122	Hydrophilic integy moment (8 descriptors)	0.112	0.003	0.045	0.008	0.805	−0.070	0.952
123	Hydrophilic integy moment (8 descriptors)	0.116	−0.085	0.014	0.007	0.759	−0.059	0.957
124	Hydrophilic integy moment (8 descriptors)	0.156	−0.281	−0.037	0.013	0.665	0.046	0.916
125	Hydrophilic integy moment (8 descriptors)	0.187	−0.592	−0.050	0.064	0.413	0.282	0.792
126	Surface rugosity	0.714	−0.206	−0.017	−0.005	0.016	−0.168	0.917
127	Interaction field surface area	0.820	0.047	−0.011	−0.004	0.022	0.137	0.987
128	Interaction field volume	0.876	0.000	−0.011	0.000	0.016	0.054	0.995
129	Hydrophilic volume (8 descriptors)	0.666	0.258	0.076	0.046	−0.068	0.280	0.978
130	Hydrophilic volume (8 descriptors)	0.507	0.312	0.063	0.067	−0.161	0.365	0.966
131	Hydrophilic volume (8 descriptors)	0.369	0.438	−0.014	0.086	−0.116	0.229	0.969
132	Hydrophilic volume (8 descriptors)	0.254	0.730	0.001	0.030	−0.009	0.067	0.978
133	Hydrophilic volume (8 descriptors)	0.123	1.014	0.017	−0.012	0.061	−0.114	0.975
134	Hydrophilic volume (8 descriptors)	0.020	1.188	−0.004	−0.050	0.126	−0.310	0.943
135	Polar volume (8 descriptors)	0.696	0.123	0.035	−0.020	−0.026	0.230	0.963
136	Polar volume (8 descriptors)	0.497	0.176	−0.010	−0.072	−0.080	0.255	0.910
137	Water accessible surface area of all atoms with positive partial charge (strictly greater than 0).	−0.080	−0.009	0.037	0.959	−0.025	0.026	0.833
138	Water accessible surface area of all atoms with negative partial charge (strictly less than 0).	−0.083	−0.014	−0.018	0.997	−0.002	0.023	0.977
139	Water accessible surface area of all hydrophobic (|*q_i_*|<0.2) atoms.	0.826	0.061	−0.004	−0.151	0.019	0.142	0.987
140	Water accessible surface area of all polar (|*q_i_*|> = 0.2) atoms.	−0.084	−0.013	−0.010	1.007	−0.005	0.024	0.982
141	Positive charge weighted surface area, ASA+ times max.	−0.063	0.002	0.017	0.996	−0.011	0.004	0.914
142	Negative charge weighted surface area, ASA− times max.	−0.045	0.002	−0.036	0.921	0.018	−0.004	0.872
143	Dipole moment calculated from the partial charges of the molecule.	−0.105	−0.019	−0.049	0.931	0.005	0.033	0.899
144	3D-Wiener index	1.166	−0.074	0.007	−0.018	−0.001	0.022	0.973
145	3D-Balaban index	0.138	−0.062	−0.810	0.038	−0.065	0.050	0.953
146	3D-Harary index	1.084	−0.105	−0.045	−0.013	−0.056	−0.084	0.969
147	Average geometric distance degree	1.033	−0.038	0.009	−0.034	0.004	0.145	0.979
148	D/D index	1.121	−0.079	−0.006	−0.015	−0.026	−0.018	0.978
149	Gravitational index G1	0.808	0.098	−0.004	0.005	−0.016	−0.209	0.968
150	Span R	0.718	−0.071	0.040	−0.033	0.021	0.600	0.951
151	Spherosity	−0.328	−0.110	0.031	−0.162	0.019	0.836	0.781
152	Asphericity	0.005	−0.254	0.206	0.041	0.024	1.160	0.797
153	Folding degree index	−0.519	0.010	0.464	−0.019	0.158	0.512	0.762
154	Aromaticity index	−0.030	−0.126	0.236	−0.165	0.052	0.186	0.819
155	HOMA total	0.310	−0.025	0.229	−0.075	−0.017	−0.031	0.892

The second factor mainly represents as an H-bond index. The parameters, e.g. H-bond donor capacity, hydrophilic volume, hydrophilic-lipophilic, number of hydrogen bond donor and acceptor atoms, etc. have large positive loading coefficients; while the parameters, e.g. log of the octanol/water partition coefficient(including implicit hydrogens), Ghose-Crippen and Moriguchi octanol-water partition coeff.(logP), asphericity, number of hydrophobic atoms, approximation to the sum of VDW surface areas of hydrophobic atoms, solvation energy, surface rugosity, van der Waals component of the potential energy, aromaticity index, etc. have large negative loading coefficients.

The third factor is a connectivity index, which is a set of miscellaneous descriptors related to molecular size, shape, and branching. Generally, the larger the connectivity index is, the larger the corresponding molecular size and branching are. Here, the third factor refers to the variables with high positive coefficients of Narumi harmonic topological index, Narumi geometric topological index, folding degree index, path/walk 5-Randic shape index, quadratic index, Narumi simple topological index (log), all-path Wiener index, unsaturation index, aromaticity index, etc. Conversely, the third factor also involves the variables with large negative loading coefficients on Balaban centric index, 3D-Balaban index, Balaban distance connectivity index, Balaban's connectivity topological index, Balaban-type index from mass weighted distance matrix, etc.

The fourth factor belongs to an index of accessible surface area. It basically represents water accessible surface area of all polar atoms, water accessible surface area of all atoms with negatively and positively partial charges, charge weighted surface area, dipole moment calculated from the partial charges of the molecule, etc. The variables vary inversely with the number of hydrogen-bond donor atoms, approximation to the sum of VDW surface areas of hydrogen bond donors, bond stretch-bend cross-term potential energy, spherosity, aromaticity index, hydrophilic factor, lowest hydrophobic energy, water accessible surface area of all hydrophobic atoms, etc.

The fifth factor refers to an integy moments index, which measures the imbalance between the center of mass of a molecule and the positions of hydrophilic or hydrophobic regions around the mass center. The parameters with relatively large positive loading on the fifth factor include hydrophobic integy moment, amphiphilic moment, hydrophilic integy moment, and hydrophobic integy moment, etc.; while the parameters with relatively large negative loading involve log of the octanol/water partition coefficient, H-bond donor capacity, hydrophilic volume, approximation to the sum of VDW surface areas of pure hydrogen bond donors, etc.

The sixth factor is related to a volume and shape index. The parameters with positive contributions to the sixth factor include the sum of spherosity, spherosity, mean square distance index, lopping centric index, eccentric, radial centric information index, span R, 3-path Kier alpha-modified shape index, folding degree index, and average eccentricity, etc.; while the parameters with negative contributions to the sixth factor are Quadratic index, ramification index, H-bond donor capacity, Hydrophilic volume, polarity number, gravitational index G1, maximal electrotopological positive variation, etc.

Taking together, once the scores of the 6 NNAAIndex factors (i.e. geometric characteristics, H-bond, connectivity, accessible surface area, integy moments index, and volume and shape) for 615 amino acids were determined, these scores can be used to characterize structural features of any peptide, to predict and design new peptides with desirable activity, along with other modeling methods. As shown in Table 1, the averaged communality value for a total of 146 out of 155 variables is ∼0.929 and most of communality values are greater than 0.8, except for 9 variables, e.g. Path/walk 5-Randic shape index, approximation to the sum of VDW surface areas of pure hydrogen bond donors, approximation to the sum of VDW surface areas of atoms typed as “other”, angle bend potential energy, hydrophobic integy moment, hydrophilic integy moment, spherosity, asphericity, folding degree index. This indicates that most variables are well presented by all the factors jointly with a high degree of reliability.

### Correlation and Difference among Six Factors of NNAAIndex

Due to the peptidic nature, certain correlation could exist between various attributes of natural amino acids and non-natural amino acids. As an oblique solution, here we used a promax algorithm with Kaiser Normalization to rotate the factors for improving the interpretation ability of the factors obtained. The correlation coefficients among the 6 factors are summarized in Table 2. It can be seen that most of the correlations show a weak or non- dependence on one another, as evidenced by very small correlation values of <0.4. However, a relatively significant intercorrelation value of 0.382 was observed between the geometric characteristics (the first factor) and the volume and shape factor (the sixth factor). This is not surprising because the volume and shape partially reflects the geometrical characteristics.

**Table 2:** The correlation coefficients among six factors.

No.	Factor 1	Factor 2	Factor 3	Factor 4	Factor 5	Factor 6
Factor 1	1.000	0.324	0.097	−0.004	0.247	0.382
Factor 2	0.324	1.000	−0.358	0.241	−0.251	0.306
Factor 3	0.097	−0.358	1.000	−0.076	0.173	−0.128
Factor 4	−0.004	0.241	−0.076	1.000	−0.232	−0.017
Factor 5	0.247	−0.251	0.173	−0.232	1.000	0.178
Factor 6	0.382	0.306	−0.128	−0.017	0.178	1.000

**Figure 1:**
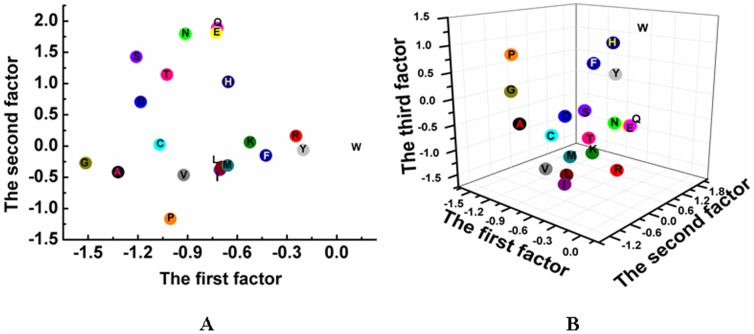
The 2- and 3-dimensional distribution of 22 natural amino acids on the first 2 and 3 factors. The A and B present the 2- and 3-dimensional distribution, respectively.

The characterization abilities of the 6 factor scores for 22 natural amino acids were assessed by 2- and 3-dimensional distributions of 22 natural amino acids on the first 2 and the first 3 factors (Figure 1), respectively. It can be seen that 22 amino acids are diversely distributed on the basis of hydrophobicity (polarity), size, and electrostatic charge, e.g. the geometric score of the first factor of Gly was less than that of other amino acids, simply because the Gly is the only amino acid without side chain, which is likely to minimize the potential steric hindrance upon interacting with other amino acids. The second distinct character was the Pro, a cyclic amino-acid, which can easily forms amide linkage with carboxyl groups of other amino acids, resulting in a cis-peptide bond that could influence its stability and steric conformation. It is simply due to the remarkable characteristic that Pro is far from other amino acids as shown in Figure 1.

**Figure 2:**
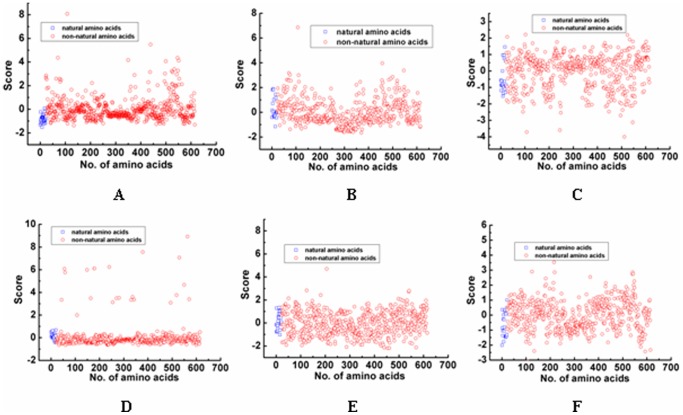
The difference of 22 natural amino acids and 593 non-natural amino acids on each factor. The A, B, C, D, E and F is the difference for geometric characteristics, H-bond, connectivity, accessible surface area, integy moments index, and volume and shape, respectively.

Figure 2 showed that the score distribution of each NNAAIndex factor for both 22 natural amino acids and 593 non-natural amino acids. The scatter score distribution of 593 non-natural amino acids was able to well cover the 22 natural amino acids, indicating peptide mimetics can be specifically obtained by the structural modification with non-natural amino acids. Upon establishment of the 6 factor scores, we are able to apply the NNAAIndex method to develop three predictive QSAR models to design peptide mimics for three different types of BTDs, ACE inhibitors, and inorganic-binding peptides, as follows.

### NNAAIndex QSAR Model for BTDs

Taste, which is often classified into four types of sweet, bitter, salty, and acid, plays a very important role in all mammals. Among them, bitter taste and sensitivity help to protect humans and organisms from being harmed by toxic substances [19]. A dataset containing 48 bitter tasting dipeptides (BTDs) [55], which has been widely used to test new structural characterization method in many different QSAR models [18]–[27], was used to train the NNAAIndex QSAR model. A total of 12 independent structural-based descriptors for each BTD were determined using the NNAAIndex scales, while the activity of these BTDs was expressed by the negative logarithm of bitter-tasting threshold concentrations (pT) as an end point [20] (Table S2). Both 12 structural-based descriptors and 1 activity-based end point were used to build a QSAR model using PLS regression. The predictive performance of the NNAAIndex QSAR model was measured by the LOO cross-validated procedure and then compared with other 13 different 2D- and 3D-QSAR methods, including z-scales [18], ISA-ECI [20], MS-WHIM [21], FASGAI [24], SZOTT [26], etc. To achieve an objective comparison, we used the same training dataset and modeling methods for all QSAR models. Final *R* ^2^, *Q* ^2^, and root-mean-square (*RMS*) error values were listed in Table 3. The results showed that the NNAAIndex/PLS model (entry 14) achieved satisfactory *R* ^2^ = 0.863 and *Q* ^2^ = 0.765. The results of the NNAAIndex/PLS model are comparable to or even superior to the results as reported by most of 2D-/3D-QSAR methods/PLS (Table 3). Upon removal of 7 redundant variables using the GA algorithm, the predictive *Q* ^2^ of the GA-PLS model (entry 15) was improved from 0.765 (entry 14) to 0.830 by ∼8.5%. Overall, different approaches for the PLS and GA-PLS models show good and consistent predictivity. Further comparison of *Q* ^2^ values across all QSAR models clearly shows that our NNAAIndex QSAR models performed better than most of existing 2D and 3D-QSAR models using 1 or 2 PCs.

**Table 3:** The performance comparison among different QSAR models of BTDs.

No.	Descriptors	Data size	Correlation methods	*A* a	*R* ^2^ b	*Q* ^2^ c	*RMS* d
1	z-scales [18]	48	PLS	2	0.824	nd	0.260
2	GRID [19]	48	PLS	1	nd^e^	0.780	nd
3	ISA-ECI [20]	48	PLS	2	0.847	nd	nd
4	MS-WHIM [21]	48	PLS	3	0.704	0.633	nd
5	MS-WHIM(extended) [21]	48	PLS	3	0.754	0.710	0.320
6	VHSE [22]	48	PLS	3	0.910	0.816	0.200
7	MARCH-INSIDE [23]	48	PLS	3	0.858	nd	0.230
8	FASGAI [24]	48	PLS	3	0.886	0.723	0.220
9	FASGAI [24]	48	GA-PLS	3	0.907	0.848	0.198
10	FASGAI [24]	24/24^f^	GA-PLS	2	0.936	0.761	0.172
11	3D-HoVAIF [25]	48	PLS	3	0.936	0. 849	nd
12	SZOTT [26]	48	PLS	2	0.908	0. 736	0.195
13	ST-SCALE [27]	48	PLS	5	0.855	0.774	0.400
14	NNAAIndex	48	PLS	2	0.863	0.765	0.238
15	NNAAIndex	48	GA-PLS	1	0.864	0.830	0.234
16	NNAAIndex	24/24^f^	GA-PLS	2	0.898	0.772	0.198

a The *A* is the number of principal component.

b The *R* ^2^ is the cumulative multiple correlation coefficient.

c The *Q* ^2^ is a cross validation square of cumulative multiple correlation coefficient by a leave-one-out procedure.

d The *RMS* is the root mean square error of modeling simulation.

e The nd shows that the correlative value is not given out.

f Two numbers separated by slashes denote the number of samples in training and test sets, respectively.

To further validate the characterization ability of NNAAIndex, we used *k*-means cluster analysis [56] to divide 48 BTDs into two groups. Each group samples were sorted from low to high activity. The 24 odd samples (i.e. the first, the third, the fifth, etc.) in each group were selected for constructing a training set, which was used to construct the QSAR model. The 24 even samples (i.e. the second, the fourth, the sixth, etc.) as a test set were used to validate the external predictive performance of the QSAR model. Table 3 shows that the *Q* ^2^ and *RMS* for internal validation were 0.772 and 0.198 (entry 16), respectively, while the *R* ^2^ and *RMS* for external validation (*Q* ^2^_ext_) were 0.806 and 0.180, respectively. Although the *R* ^2^ of 0.898 in NNAAIndex/GA-PLS was slightly smaller than the *R* ^2^ of 0.936 in FASGAI/GA-PLS, the *Q* ^2^ and *Q* ^2^_ext_ in NNAAIndex/GA-PLS were larger than those in FASGAI/GA-PLS (*Q* ^2^ = 0.761 and *Q* ^2^_ext_ = 0.797 [24]), indicating that the NNAAIndex/GA-PLS model has satisfactory external predictive ability.

**Figure 3:**
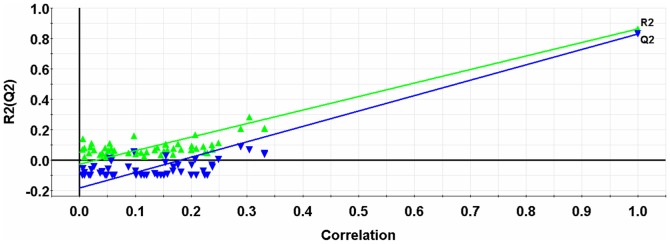
Plot of the 50-random-permutation validation for the GA-PLS model of BTDs. The intercepts of the *R* ^2^- and *Q* ^2^-regression lines with the ordinate axis are −0.026 and −0.183, which are below limits of *R* ^2^<0.300 and *Q* ^2^<0.050, respectively.

We also performed a response permutation (i.e. Y-randomization response) test to assess the robustness of the NNAAIndex QSAR model. Figure 3 displays the response permutation results through 50-random-permutation tests for the GA-PLS model. The interceptions of the *R* ^2^- and *Q* ^2^-regression lines with the ordinate axis were −0.026 and −0.183, respectively, which were below the limits of *R* ^2^<0.300 and *Q* ^2^<0.050 [57]. This is a clear evidence that our model is not affected by any chance correlation, i.e. the probability to obtain a similar or better model using random numbers is zero, and it is likely to depict a true linear relationship between the NNAAIndex descriptors and pT values.

Figure S1 shows the centered and scaled coefficients of the GA-PLS model which are used to describe the extent of influence of 5 independent variables (integy moments index, and volume and shape of the first residue, geometric characteristics, H-bond, and integy moments index of the second residue) on the activity of BTDs. A positive coefficient value indicates that the variable prefers to improve the BTD activity, and *vice versa* for a negative value. It can be clearly seen in Figure S1 that the 5th, 6th, 7th and 11th variables corresponding to integy moments index, and volume and shape of the first residue, geometric characteristics and integy moments index of the second residue have positive coefficients, while only the 8th variable corresponding to H-bond index of the second residue has a negative coefficient.

To enable the rational design of new peptidomimetics with high bitter taste sensitivity, we developed an in-house C++ program to map out amino acid preferences at different sequence positions based on the GA-PLS coefficients (Figure S1), along with the scores of 615 amino acids (Table S1). To obtain BTD peptidomimetics with higher predictive activity, we alternatively selected the first amino acid with the score of both integy moments and volume and shape larger than 2.0, the second amino acid with the score of geometric characteristics and integy moments larger than 2.0, and H-bond less than −0.5. This design strategy led to 1178 new molecules with high potent predictive activity, and 613 out of 1178 new peptidomimetics (Table S3) achieved the higher predictive activity than the 48 training peptides.

**Figure 4:**
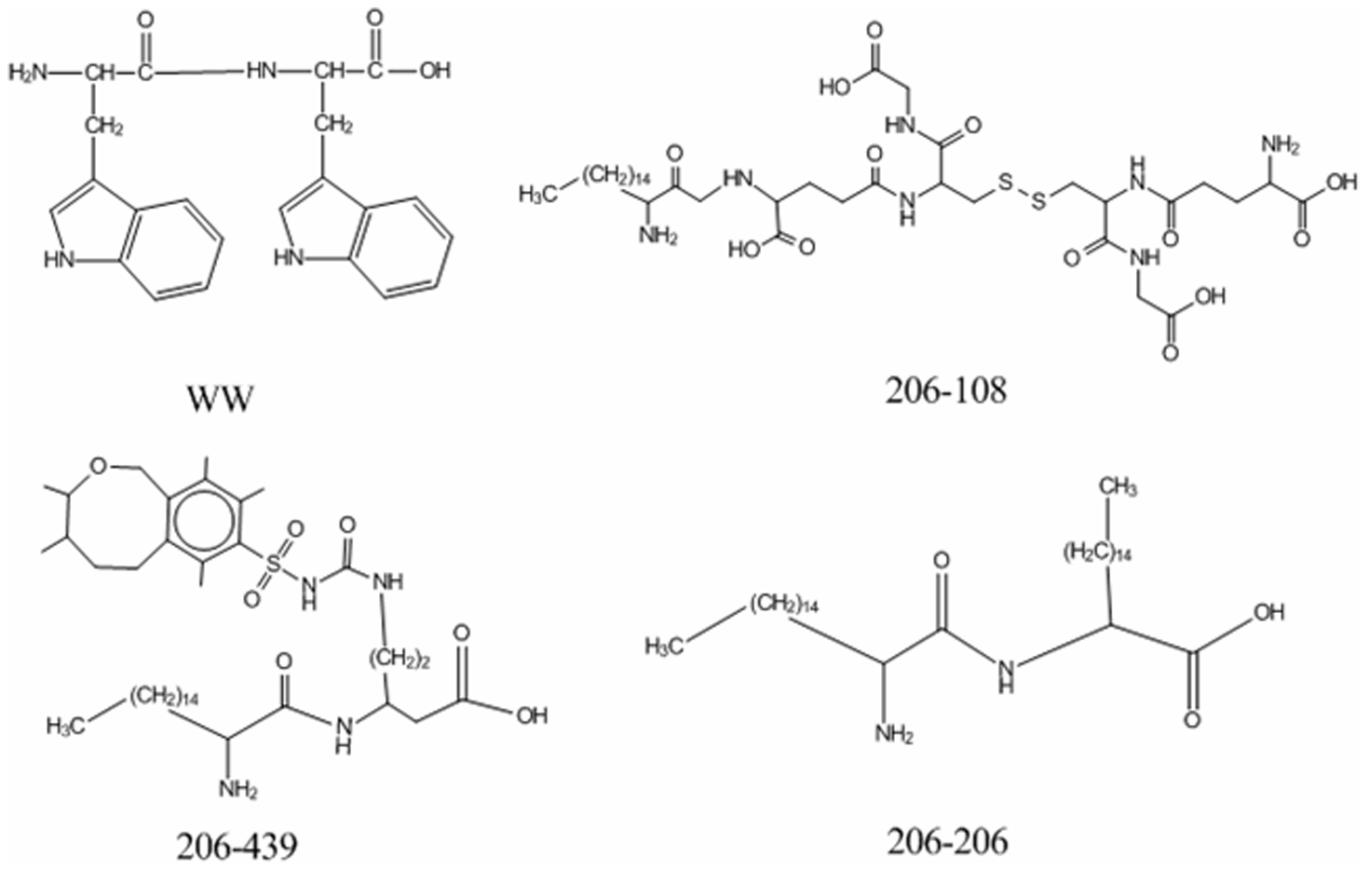
The newly designed peptide mimetics for BTDs. The forty-sixth sample in training set, WW, is regarded as a template to design molecules. The activity of WW, 206-108, 206-439 and 206-206 is 3.60, 9.78, 9.20, and 9.17, respectively.

As a proof-of-concept, Figure 4 shows that 3 different designed BTD mimetics (namely 206-108, 206-439, and 206-206) had potential high activity. These three peptides were all derived from the dipeptide (WW) by replacement of the first residue with the 206th non-natural amino acid and the second residue with the 108th, 439th, or 206th non-natural amino acid, respectively. According to the relationship between the variables and the activity of BTDs as discussed above, integy moments index of the first residue, geometric characteristics and integy moments index of the second residue contribute positively to the activity of BTDs. The integy moments and volume and shape index of the 206th molecule were 4.692 and 4.202 (Table S1), which are the largest values in all 615 amino acids, respectively. The geometric characteristics of the 106th, 437th, and 206th molecule were also relatively large. By comparison with the structure of WW, we observed that the activity of 3 newly designed peptide mimics was greatly improved by substitution of two natural residues with non-natural residues, which should be validated by future experiments.

### NNAAIndex QSAR Model for ACE Inhibitors

Angiotensin-converting enzyme (ACE) inhibitors have been used for over 30 years to treat cardiovascular diseases. In addition to their antihypertensive effects, ACE inhibitors are now also widely used for congestive heart failure, acute myocardial infarction, and diabetic nephropathy [58]. However, ACE inhibitor therapy may also cause some adverse effects including dry cough [59] and activation of the renin-angiotensin system [60]. To assess the structure-activity relationship of ACE inhibitors, a dataset of 58 ACE inhibitor dipeptides as reported by Collantes and Dunn [20] was used to build the second NNAAIndex QSAR model for design of new ACE inhibitors. This dataset has been often used as a model set to test and validate the performance of different QSAR models [18]–[22], [24], [28], [29]. In this dataset, the dipeptide sequences were characterized by 12 NNAAIndex scores (i.e. each amino acid by 6 NNAAIndex scores), with *p*IC_50_ values ranging from 1.77 to 5.80 as an end point. The structures and bioactivity for the 58 ACE dipeptide inhibitors are summarized in Table S4. To validate the predictive performance of our NNAAIndex method, we collected the modeling statistics from other 11 different QSAR models for comparison (Table 4). The LOO cross-validated PLS analysis led to *R* ^2^ = 0.749 and *Q* ^2^ = 0.719 using 2 PCs, which were comparable to those values from other QSAR/PLS methods in Table 4. To further improve the quality of the NNAAIndex model, we used GA to remove some redundant descriptors, resulting in the improvement in both *R* ^2^ = 0.803 and *Q* ^2^ = 0.779 (entry 13, Table 4). Consistent with the NNAAIndex model for the BTDs as described above, the number of PCs used in the PLS analysis has influence on the quality of the QSAR models. It appears that the minimum number of PC yields the optimal results, due to the minimization of data overfitting during the model training process.

**Table 4:** The performance comparison among different QSAR models of ACE inhibitors.

No.	Descriptors	Data size	Correlation methods	*A* a	*R* ^2^ b	*Q* ^2^ c	*RMS* d
1	z-scales [18]	58	PLS	2	0.770	nd^e^	nd
2	GRID [19]	58	PLS	1	0.744	nd	0.500
3	ISA-ECI [20]	58	PLS	2	0.700	nd	nd
4	MS-WHIM(rotameric) [21]	58	PLS	6	0.657	0.541	nd
5	MS-WHIM(extended) [21]	58	PLS	2	0.708	0.637	0.540
6	VHSE [22]	58	SMR-PLS	1	0.770	0.745	0.480
7	FASGAI [24]	58	PLS	1	0.760	0.728	0.495
8	FASGAI [24]	58	GA-PLS	1	0.796	0.775	0.456
9	FASGAI [24]	29/29^f^	GA-PLS	1	0.869	0.835	0.357
10	3D-HoVAIF [28]	58	GA-PLS	3	0.857	0.811	0.376
11	T-scale [29]	58	SMR-PLS	2	0.845	0.786	0.390
12	NNAAIndex	58	PLS	2	0.749	0.719	0.511
13	NNAAIndex	58	GA-PLS	2	0.803	0.779	0.453
14	NNAAIndex	29/29^f^	GA-PLS	1	0.852	0.832	0.369

a The *A* is the number of principal component.

b The *R* ^2^ is the cumulative multiple correlation coefficient.

c The *Q* ^2^ is a cross validation square of cumulative multiple correlation coefficient by the leave-one-out procedure.

d The *RMS* is the root mean square error of modeling simulation.

e The nd shows that the correlative value is not given out.

f Two numbers separated by slashes denote the number of samples in training and test sets, respectively.

Table 4 shows that the NNAAIndex/GA-PLS model had relative higher *Q* ^2^ than that of the NNAAIndex/PLS model. Besides, the *Q* ^2^ of the NNAAIndex/GA-PLS model was merely smaller than 3D-HoVAIF [28] as a 3-dimentional structural characterization approach and T-scale [29] as a topologically structural characterization approach. This may provide some hints to improve the characterization ability of the NNAAIndex model by considering 3D structural and/or other topological information in our future work.

Following the same method to validate the characterization ability of the BTD dataset, we equally divided 58 ACE inhibitors into the 29 training samples for the development of the QSAR model and the remaining 29 test samples for the validation of the QSAR model using the NNAAIndex/GA-PLS. *Q* ^2^ = 0.832 and *RMS* = 0.369 for internal validation and *Q* ^2^_ext_ = 0.731 and *RMS* = 0.500 for external validation were obtained, respectively, further confirming that the external predictive ability of our NNAAIndex/GA-PLS model is superior to that of the FASGAI/GA-PLS model as previously developed by our group (*Q* ^2^_ext_ = 0.706) [24].

**Figure 5:**
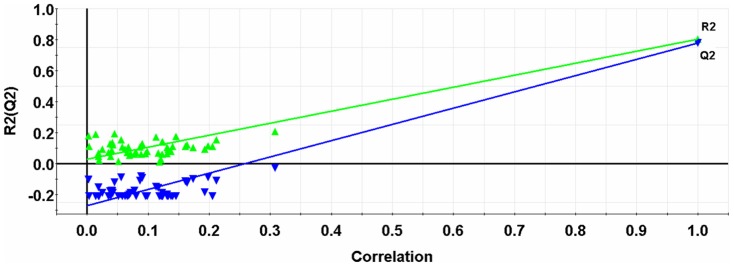
Plot of the 50-random-permutation validation for the GA-PLS model of ACE inhibitors. The intercepts of the *R* ^2^- and *Q* ^2^- lines with the ordinate axis are 0.029 and −0.270, which are below limits of *R* ^2^<0.300 and *Q* ^2^<0.050, respectively.

The NNAAIndex model was further validated using the response permutation test, which was performed by rebuilding the models using randomized activities of the training set, followed by the subsequent assessment of the model statistics. This test was repeated 50 times to obtain reliable statistics on model robustness. Figure 5 showed that the interceptions of *R* ^2^ and *Q* ^2^ were 0.029 and −0.270, respectively. This indicates the probability of chance correlation is very low (*p*<0.001), reinforcing the validity of the model. Figure S2 shows the GA-PLS regression coefficients for 8 variables. Two variables of 7 and 9, corresponding to geometric characteristics and connectivity of the second residue, respectively, had large positive regression coefficients. Conversely, 2 variables of 3 and 10 that represent the connectivity of the first residue and the solvent accessible surface area of the second residue respectively, exhibited large negative coefficients. Other 4 variables corresponding to geometric characteristics, H-bond, accessible surface area of the first residue, and H-bond of the second residue appeared to have negligible effects on ACE inhibitory activity.

**Figure 6:**
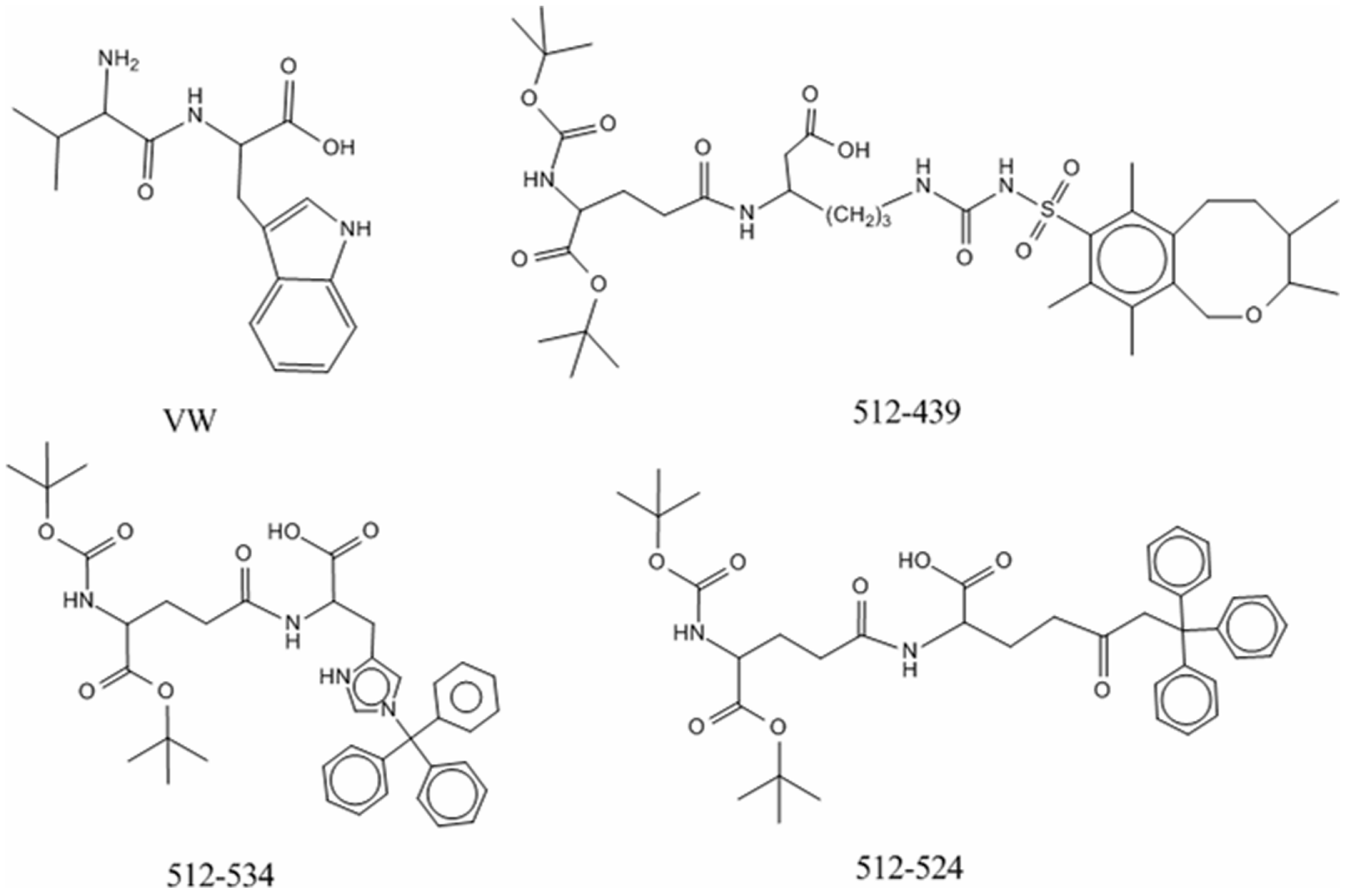
The newly designed peptide mimetics for ACE inhibitors. The first sample in training set, VW, is regarded as a template to design molecules. The activity of VW, 512-439, 512-534 and 512-524 is 5.80, 8.33, 8.04, and 8.04, respectively.

Based on the preferential variables in the GA-PLS model and the scores of amino acids in and Figure S2, a total of 616 potent peptide mimetics of ACE inhibitors was designed and their inhibitory activities were evaluated by the GA-PLS model. A total of 205 out of 616 peptides showed higher inhibitory activity than that of 58 peptides in the training set (Table S5). Figure 6 shows three new peptide mimics of ACE inhibitors with highly predictive activity. The activity of ACE inhibitors has a negative correlation with the connectivity index of the first residue, i.e. the smaller the value is, the higher the activity is. The first residue of the dipeptide (VW) is replaced by the 512th molecule with a smallest connectivity index of −4.006 among all amino acids. Through comparison, the connectivity index of the 512th molecule is significantly less than that of the W residue (1.454) (Table S1), and the side chain of the 512th molecule has multiple benzene rings, which would lead to complicated spatially geometric features and molecular branching as a reflection of connectivity. Thus, it is expected that the 512-W mutant has a higher inhibitory ability. The geometric characteristics of the second residue are positively correlated with biological activity of ACE inhibitors. The second residue was replaced by the 439th, 534th, or 524th molecule separately, whose geometric scores are 5.484, 3.388, and 3.233, respectively. It should be noted, although the geometric scores of the 439th, 534th, or 524th molecule are not the largest ones in factor scores of all 615 amino acids, the new designed molecules (512-439, 512-534 and 512-524) rank as top 3 candidates in all 205 designed ACE inhibitors, together with a modification of the 510 molecule at the first residue, suggesting that single-residue mutation is not sufficient enough to ensure the improved activity of ACE inhibitors.

It is of great interest to screen and examine if some dipeptides derived from a complete sequence space possess both BTD and ACE inhibition activities. If any sequence possesses dual predicted activities in BTD and ACE inhibition, this sequence is likely to be served as a promising pharmaceutical lead compound for experimental tests. To achieve this, we used the NNAAIndex/GA-PLS model to predict new 436 (426) dipeptides, which exclude 48 (58) sequences in a training BTD (ACE inhibitor) set from a complete dipeptide sequence space consisting of 484 sequences, for their BTD activity (Table S6) or ACE inhibitory activity (Table S7), separately. The results, however, showed that the predicted pT values of 436 dipeptides using the NNAAIndex/GA-PLS model of BTDs were lower than that of the 46th sample (WW) with a relative largest activity in all 48 BTD training samples. Similarly, the predicted pIC_50_ values of 426 dipeptides using the NNAAIndex/GA-PLS model of ACE inhibitors were lower than that of the 1st sample (WW) with a relative largest activity in all 58 ACE-inhibitor training samples. Moreover, we observed that the activities of the designed peptidomimetics for BTDs and ACE inhibitors were significantly higher than those of the designed coded-amino-acid peptides, indicating the advantage of peptidomimetics theoretically designed using the NNAAIndex scales.

### NNAAIndex QSAR Model for Inorganic-binding Peptides

Bioactive peptides binding to inorganic surfaces play a central role in many scientific and technological applications including nanoparticle synthesis [61], surface quality control [62], molecular linkers [63], peptide sensors [64], and self-assembly nanostructures [65]. Phage display approach has been widely used to discover new inorganic-binding peptides with a wealth of data [62], but such brute-force and high-cost method is very sensitive to experimental conditions such as peptide purification, peptide concentration, large-scale peptide production, and/or cellulose support. Additionally, this method provides little structural and binding information of peptides on solid surfaces, which negatively impacts the ability to rationally design or post-engineering new sequences for further improving their activity. Here, a total of 20 heptapeptides with binding affinity to mica surface [66] (Table S8) was used to construct the third QSAR model using LDA, which was used to classify inorganic-binding peptides into two groups (namely, strong and moderate groups) based on predicted binding affinity.

Due to the relatively small number of available peptides, to achieve meaningful model statistics, the validation process was also ensured using the LOO cross-validated procedure. The discriminant performance was evaluated using a variety of statistical values: (1) *accuracy*, characterizes the percentage of all chemicals which is correctly identified in each group; (2) *sensitivity*, measures the percentage of biologically active chemicals (in this case, i.e. strong-binding peptides) which is correctly identified; (3) *specificity*, measures the percentage of biologically inactive (i.e. moderate-binding peptides) which is correctly identified; and Matthews correlation coefficient (*MCC*) accounting for over- and under-predictions indicates how the predictions relate to the target observations [67], [68]. The discriminant performance by the LOO cross validation achieved a performance of *accuracy* = 75.00%, *sensitivity* = 75.00%, *specificity* = 75.00%, and *MCC* = 0.492. This confirms that the NNAAIndex QSAR model has a favorable predictive ability for mica-binding peptides.

**Figure 7:**
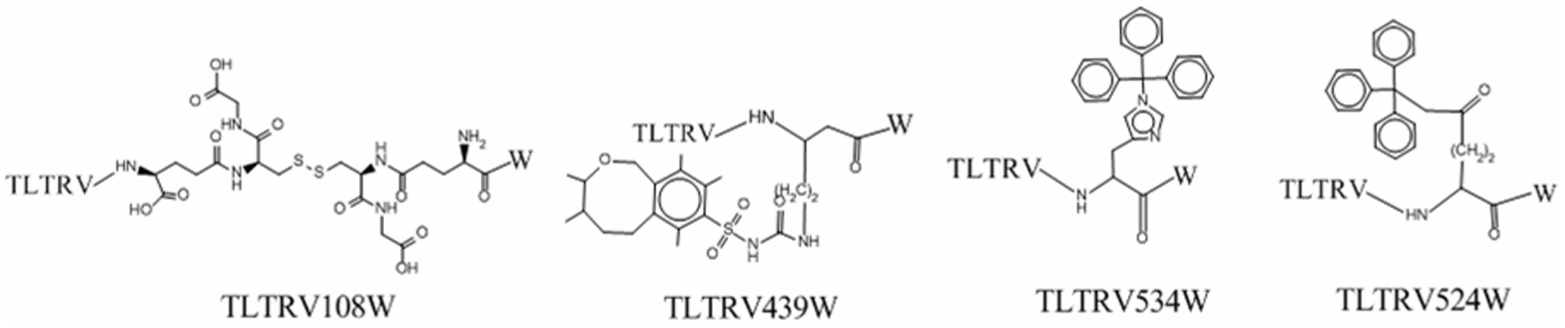
The newly designed mica-binding peptide mimetics. The 13th sample in training set, TLTRVGW, is regarded as a template to design molecules. The predicted score of TLTRVGW, TLTRV108W, TLTRV439W, TLTRV534W and TLTRV524W is 0.94, 1.00, 1.00, and 1.00, respectively.

To investigate how the variables influence the activity of mica-binding peptides, we analyzed the standardized canonical discriminant function coefficients of the LDA model (Figure S3). We observed that only 2 out of 42 variables were selected by the LDA model. Two variables with positive coefficients corresponded to geometric characteristics and accessible surface area of the sixth residue. The interpretation of the QSAR model indicates that the use of large geometric characteristics and solvent accessible surface area of the sixth residue of the heptapeptides can improve their mica-binding ability. We designed a total of 54 mica-binding peptidomimetics *in silico* modified by non-natural amino acids (Table S9). Among these designs, 37 peptidomimetics exhibited strong predictive binding affinity. We also found the newly-designed peptide mimics with an increased predictive mica-binding activity being acquired by modifying main chain and side chain (Figure 7). The 108th, 439th, 534th, and 524th molecules had complicated geometric features such as extended lateral aromatic moieties and branching, which are positively contributed to the mica-binding affinity of peptide mimics. Moreover, large solvent accessible surface area of those molecules enables to improve the binding affinity of the newly designed peptide mimics.

It is of interest to compare the details and performance of QSAR models between our previous FASGAI model [24] and current NNAAindex model. The comparison between previous work (FASGAI) and current work (NNAAindex) seems insufficient. For instance, for BTDs, the *Q* ^2^ value is 0.848 for FASGAI/GA-PLS and 0.830 for NNAAIndex/GA-PLS, respectively (Table 3). This could be due to the intrinsic differences as follows: (1) FASGAI and NNAAIndex models used different sources to obtain the physicochemical properties of amino acids, i.e., the FASGAI used the AAindex database [69] to derive 516 experimentally-based properties, while the NNAAIndex used the E-dragon [47] and MOE programs [48] to acquire 384 computationally-based properties, simply because there are no available physicochemical properties for 593 non-natural amino acids by experiments. Consequently, the computationally calculated properties may inevitably bring some uncertainties to the model, thus leading to the limited improvement of the NNAAIndex method to some extents. (2) FASGAI and NNAAIndex models used different scale factors to present amino acids, i.e., the FASGAI represents hydrophobicity, alpha and turn propensities, bulky properties, compositional characteristics, local flexibility, and electronic properties, while the NNAAIndex represents geometric characteristics, H-bond, connectivity, accessible surface area, integy moments index, and volume and shape. Apparently, different factor properties in both models reflect fundamental difference for capturing and characterizing the structural features of natural and non-natural amino acids, resulting in different results and accuracies. (3) More importantly, the FASGAI characterized the structural features of the peptides consisting of 20 natural amino acids, while the NNAAindex characterized the structural features of peptides and peptidomimetics consisting of 22 natural and 593 non-natural amino acids. Therefore, FASGAI and NNAAindex models have different characterization ability for different peptide datasets. Because of this, we can not arbitrarily deny the characterization ability for any one of FASGAI and NNAAIndex models.

## Conclusions

In this work, a new index, NNAAIndex, was proposed to represent the structures of 22 natural and 593 non-natural amino acids. To test the applicability of the NNAAIndex method on three different datasets, three predictive QSAR models were developed to identify the contributing descriptors to the most to the activity of BTD, inhibitory activity of ACE inhibitors, and binding affinity of mica-binding peptides using a combination of the NNAAIndex and PLS regression or LDA method. As compared to the prediction power of other 2D- and 3D-QSAR models, the NNAAIndex QSAR model using 6 field descriptors yielded satisfactory statistical results. In addition, we provided a large pool for BTD, ACE inhibitors, and inorganic-binding peptides for experimental validation in the future. Our results suggest that the NNAAIndex model can be generally applied to in *silico* design any other new natural or non-natural peptidomimetics in a high throughput manner since it only requires 2D fingerprints as inputs.

## Supporting Information

Figure S1 The centered and scaled coefficients for the GA-PLS model of BTDs. Four positive coefficients for the variables of 5, 6, 7, and 11 correspond to integy moments index, and volume and shape of the first residue, geometric characteristics and integy moments index of the second residue, respectively. One negative coefficient for the variable of 8 corresponds to H-bond of the second residue, respectively.
(TIF)

Figure S2 The centered and scaled coefficients of the GA-PLS model of ACE inhibitors. Three positive coefficients for the variables of 1, 7, and 9 correspond to geometric of the first residue, geometric characteristics and connectivity of the second residue, respectively. Five negative coefficients for the variables of 2, 3, 4, 8 and 10 correspond to H-bond, connectivity, accessible surface area of the first residue, H-bond and accessible surface area of the second residue, respectively.
(TIF)

Figure S3 The standardized coefficients of the LDA model of mica-binding peptides. Two positive coefficients for the variables of 31 and 34 correspond to geometric characteristics and accessible surface area of the sixth residue, respectively.
(TIF)

Table S1 Scores of six NNAAIndex factors for 22 natural and 593 non-natural amino acids.
(DOC)

Table S2 Sequences of BTDs with the observed and predicted activities.
(DOC)

Table S3 Computationally designed peptidomimetics of BTDs.
(DOC)

Table S4 Sequences of ACE inhibitors with the observed and predicted activities.
(DOC)

Table S5 Computationally designed peptidomimetics of ACE inhibitors.
(DOC)

Table S6 Predicted activities of BTDs composed of 22 natural amino acids by excluding the 48 training samples using the QSAR model of BTDs.
(DOC)

Table S7 Predicted activities of ACE inhibitors composed of 22 natural amino acids by excluding the 58 training samples using the QSAR model for ACE inhibitors.
(DOC)

Table S8 Sequences of 20 inorganic-binding peptides with actual group and predicted group.
(DOC)

Table S9 Computationally designed mica-binding peptidomimetics.
(DOC)
