# Supplementary material for: An Index for Characterization of Natural and Non-Natural Amino Acids for Peptidomimetics
Source: PLoS One. 2013 Jul 23;8(7):e67844. doi: 10.1371/journal.pone.0067844 (PMC3720802; doi:10.1371/journal.pone.0067844)
Supplement: Table S1 — Scores of six NNAAIndex factors for 22 natural and 593 non-natural amino acids. (DOC) [file pone.0067844.s004.doc]

# Table S1. Scores of six NNAAIndex factors for 22 natural and 593 non-natural amino acids

| **No.** | **Molecule** | **Factor 1** | **Factor**  **2** | **Factor 3** | **Factor 4** | **Factor 5** | **Factor 6** |
| --- | --- | --- | --- | --- | --- | --- | --- |
| 1(A) | OC(=O)C(N)C | -1.319 | -0.418 | -0.571 | 0.490 | -0.165 | -2.024 |
| 2(R) | OC(=O)C(N)CCCNC(=N)[NH-] | -0.245 | 0.161 | -1.192 | 0.117 | 1.278 | 0.349 |
| 3(N) | OC(=O)C(N)CC(=O)N | -0.913 | 1.796 | -0.880 | 0.061 | -0.775 | -0.858 |
| 4(D) | OC(=O)C(N)CN | -1.183 | 0.705 | -0.603 | 0.222 | -0.748 | -1.376 |
| 5(C) | SCC(N)C(O)=O | -1.068 | 0.023 | -0.808 | 0.537 | -0.113 | -1.055 |
| 6(Q) | OC(=O)C(N)CCC(=O)N | -0.721 | 1.887 | -0.887 | -0.030 | -0.251 | -0.281 |
| 7(E) | OC(=O)C(N)CCC(O)=O | -0.726 | 1.814 | -0.870 | 0.095 | -0.671 | -0.005 |
| 8(G) | OC(=O)CN | -1.515 | -0.272 | -0.009 | 0.606 | -0.496 | -1.792 |
| 9(H) | OC(=O)C(N)Cc1nc[nH]c1 | -0.654 | 1.027 | 0.967 | -0.020 | 0.166 | -0.304 |
| 10(I) | OC(=O)C(N)C(CC)C | -0.705 | -0.380 | -1.537 | 0.055 | 0.516 | -1.071 |
| 11(L) | OC(=O)C(N)CC(C)C | -0.694 | -0.336 | -1.357 | 0.061 | 0.774 | -0.807 |
| 12(K) | OC(=O)C(N)CCCCN | -0.523 | 0.063 | -0.949 | -0.104 | 0.427 | 0.302 |
| 13(M) | S(CCC(N)C(O)=O)C | -0.657 | -0.310 | -0.988 | 0.074 | 1.316 | 0.321 |
| 14(F) | OC(=O)C(N)Cc1ccccc1 | -0.427 | -0.150 | 0.783 | -0.304 | 1.049 | 0.130 |
| 15(P) | OC(=O)C1NCCC1 | -1.004 | -1.165 | 0.941 | 0.285 | 1.026 | -1.492 |
| 16(S) | OC(=O)C(N)CO | -1.210 | 1.428 | -0.631 | 0.342 | -1.030 | -1.424 |
| 17(T) | OC(C(N)C(O)=O)C | -1.024 | 1.143 | -1.107 | 0.201 | -0.636 | -1.408 |
| 18(W) | OC(=O)C(N)Cc1c2c([nH]c1)cccc2 | 0.072 | -0.027 | 1.454 | -0.444 | 0.767 | 0.071 |
| 19(Y) | Oc1ccc(cc1)CC(N)C(O)=O | -0.202 | -0.061 | 0.638 | -0.236 | -0.101 | 0.459 |
| 20(V) | OC(=O)C(N)C(C)C | -0.924 | -0.464 | -1.305 | 0.165 | 0.450 | -1.416 |
| 21(U) | [SeH]CC(N)C(O)=O | -1.189 | 0.003 | -0.901 | 0.677 | -0.089 | -1.319 |
| 22(O) | OC(=O)C(N)CCCCNC(=O)C1N=CCC1C | -0.829 | 0.009 | -0.865 | -0.400 | 0.078 | 1.001 |
| 23 | O(C)c1ccc(cc1)C(NC(=O)CC(N)C(O)=O)c1ccc(OC)cc1 | 2.674 | 0.860 | 0.537 | -0.286 | 0.683 | 0.334 |
| 24 | OC(=O)C(N)CC(=O)NC(c1ccccc1)(c1ccccc1)c1ccccc1 | 2.853 | 0.133 | 1.106 | -0.416 | 1.354 | -0.937 |
| 25 | OC(=O)C(NC(=O)C)CC(=O)N | -0.373 | 1.928 | -1.784 | -0.057 | -1.689 | -0.436 |
| 26 | O(C)c1cc(cc(OC)c1)C(OC(=O)NC(CC(=O)N)C(O)=O)(C)C | 2.527 | 1.432 | -0.971 | -0.292 | -0.128 | 0.520 |
| 27 | O1c2c(cccc2)C(NC(=O)CC(N)C(O)=O)c2c1cccc2 | 1.762 | 0.472 | 2.068 | -0.176 | 0.837 | -0.741 |
| 28 | Ic1ccccc1C(=O)NCC(O)=O | 0.157 | 0.028 | 0.113 | -0.334 | 1.066 | 0.613 |
| 29 | OC(=O)CNC(=O)c1ccccc1 | -0.227 | 0.086 | 0.724 | -0.162 | 0.834 | 0.459 |
| 30 | O(C)c1cc(OC)cc(OC)c1CNCC(O)=O | 0.850 | 0.412 | -0.458 | -0.437 | -0.153 | 0.856 |
| 31 | SC(C(=O)NCC(O)=O)C | -0.534 | 0.006 | -1.251 | 0.245 | -0.560 | 0.046 |
| 32 | OC(=O)CN(CCCN)CCCN | 0.119 | 0.815 | -1.883 | -0.337 | -1.666 | 1.274 |
| 33 | OC(=O)CNC(c1ccccc1)(c1ccccc1)c1ccccc1 | 1.830 | -1.055 | 1.121 | -0.648 | 0.728 | -1.039 |
| 34 | OC(=O)CNC(=O)c1ccc(N)cc1 | 0.000 | 0.343 | 0.586 | -0.238 | -0.566 | 0.486 |
| 35 | OC(=O)CNCc1ccccc1 | -0.358 | -0.280 | 0.958 | -0.320 | 0.946 | 0.617 |
| 36 | S(=O)(=O)(NCC(O)=O)c1ccc(cc1)C | 0.207 | 0.580 | -0.091 | -0.476 | 0.807 | 0.594 |
| 37 | O(C(=O)CC(N)C(O)=O)C1CCCCC1 | 0.296 | 0.467 | 0.197 | -0.190 | 1.584 | 0.843 |
| 38 | O(C(=O)CC(N)C(O)=O)CC=C | -0.310 | 0.527 | -1.256 | -0.116 | 1.612 | 0.880 |
| 39 | O(Cc1ccccc1)C(=O)C(N)CC(O)=O | 0.367 | 0.704 | 0.476 | -0.303 | 1.648 | 1.355 |
| 40 | OC(=O)CC(N)C(=O)N | -0.877 | 1.292 | -0.984 | 0.132 | -1.566 | -0.937 |
| 41 | O(C(=O)C(N)CC(O)=O)CC=C | -0.295 | 0.784 | -1.369 | -0.069 | 0.703 | 0.970 |
| 42 | OC(=O)CNC=O | -1.177 | 0.055 | -0.211 | 0.533 | -1.916 | -0.558 |
| 43 | OC(=O)CNCC | -1.092 | -0.340 | -0.346 | 0.192 | 0.191 | -0.360 |
| 44 | OC(=O)C(N)Cc1cc([N+](=O)[O-])ccc1 | 0.093 | 0.114 | 0.233 | 3.339 | 0.028 | 0.468 |
| 45 | Brc1ccccc1CC(N)C(O)=O | -0.101 | -0.495 | 0.191 | -0.473 | 1.292 | 0.004 |
| 46 | O(CC)C(=O)NC(Cc1ccccc1)C(O)=O | 0.620 | 0.289 | -0.099 | -0.549 | -0.268 | 0.678 |
| 47 | OC(=O)C(NC(=O)C)Cc1ccccc1 | 0.156 | 0.079 | 0.145 | -0.471 | -0.280 | 0.162 |
| 48 | O(C(=O)C(NC(=O)CC(N)C(O)=O)Cc1ccccc1)C | 1.426 | 1.871 | -0.338 | -0.387 | 0.782 | 0.928 |
| 49 | OC(=O)C(N)CC(O)=O | -0.941 | 1.704 | -0.794 | 0.175 | -0.930 | -0.612 |
| 50 | OC(=O)C(NC(=O)C)CC(O)=O | -0.415 | 2.111 | -1.697 | -0.011 | -1.219 | -0.226 |
| 51 | OC(=O)C(N)Cc1ccc(cc1)-c1ccccc1 | 0.814 | -0.807 | 1.372 | -0.451 | 2.140 | 0.908 |
| 52 | O(Cc1ccccc1)C(=O)CC(N)C(O)=O | 0.366 | 0.535 | 0.596 | -0.324 | 1.441 | 1.375 |
| 53 | O(C(=O)CC(N)C(O)=O)C | -0.726 | 0.735 | -1.060 | 0.132 | 0.107 | -0.001 |
| 54 | O(C(=O)C(N)CC(O)=O)C | -0.731 | 0.632 | -1.097 | 0.077 | -1.015 | -0.042 |
| 55 | O(C(C)(C)C)C(=O)NCC(O)=O | -0.178 | -0.141 | -1.894 | -0.058 | 0.258 | 0.256 |
| 56 | OC(=O)C(N)CCN=[N+]=[N-] | -0.717 | 1.568 | -0.672 | 6.076 | -1.432 | 0.616 |
| 57 | O(CC=C)C(=O)NC(C(O)=O)C | -0.243 | 0.351 | -1.509 | -0.120 | -0.067 | 0.793 |
| 58 | OC(=O)C(N)CN=[N+]=[N-] | -0.963 | 1.662 | -0.611 | 5.746 | -0.816 | -0.111 |
| 59 | OC(=O)C1N(CCC1)C(=O)C(N)C | -0.221 | 0.540 | 0.008 | -0.086 | 0.140 | -0.731 |
| 60 | OC(=O)C(NC(=O)C(N)C)Cc1c2c([nH]c1)cccc2 | 1.198 | 0.564 | 0.720 | -0.646 | -0.224 | -0.225 |
| 61 | OC(=O)C(NC(=O)CN)C | -0.651 | 0.793 | -1.204 | -0.011 | -0.976 | -0.293 |
| 62 | OC(=O)C(NC(=O)CN)CC(O)=O | -0.213 | 2.629 | -1.761 | -0.190 | -1.476 | -0.012 |
| 63 | OC(=O)C(NC(=O)CN)Cc1nc[nH]c1 | 0.117 | 1.820 | 0.256 | -0.362 | -1.164 | -0.025 |
| 64 | Oc1ccc(cc1)CC(NC(=O)C(NC(=O)CN)CC(C)C)C(O)=O | 2.588 | 2.048 | -1.014 | -0.473 | -0.737 | 0.557 |
| 65 | OC(=O)CN(C(=O)CN)C | -0.603 | -0.171 | -1.401 | 0.182 | -1.148 | -0.631 |
| 66 | OC(=O)CNC(=O)C(N)Cc1ccccc1 | 0.431 | 0.646 | 0.420 | -0.424 | 0.999 | 1.173 |
| 67 | OC(=O)C(NC(=O)CN)Cc1ccccc1 | 0.359 | 0.887 | 0.111 | -0.541 | 0.123 | 0.282 |
| 68 | OC(=O)C1N(CCC1)C(=O)CN | -0.439 | 0.556 | 0.367 | -0.073 | -0.368 | -0.596 |
| 69 | OC(=O)C(N)CCC(=O)NC(C(O)=O)C | 0.317 | 2.613 | -1.734 | -0.144 | -1.306 | 1.226 |
| 70 | OC(=O)C(NC(=O)C(N)CC(O)=O)Cc1ccccc1 | 1.117 | 2.556 | -0.082 | -0.438 | 0.959 | 0.731 |
| 71 | OC(=O)C(NC(=O)C(NC(=O)CNC(=O)c1ccccc1)Cc1nc[nH]c1)CC(C)C | 4.359 | 3.163 | 0.737 | 0.286 | -0.606 | -0.340 |
| 72 | OC(C(NC(=O)c1ccccc1)C(O)=O)C | 0.287 | 1.020 | 0.115 | -0.325 | -0.355 | 0.307 |
| 73 | OC(=O)C1NCC(C1)=C | -0.904 | -0.533 | 0.820 | 0.089 | -0.297 | -1.114 |
| 74 | Oc1ccc(cc1)CC(NC(=O)C)C(O)=O | 0.369 | 0.558 | 0.063 | -0.428 | -1.107 | 0.453 |
| 75 | P(Oc1ccc(cc1)CC(N)C(O)=O)(O)(O)=O | 0.580 | 3.018 | 0.123 | -0.370 | -0.804 | 1.329 |
| 76 | OC(=O)C(Cc1c2c([nH]c1)cccc2)C | 0.132 | -1.373 | 1.264 | -0.536 | 0.521 | -0.210 |
| 77 | O(CC)c1ccc(cc1)CC(N)C(O)=O | 0.309 | -0.418 | 0.299 | -0.418 | 0.866 | 1.156 |
| 78 | P(OCc1ccccc1)(OCc1ccccc1)(OCC(N)C(O)=O)=O | 2.569 | 1.069 | 0.576 | -0.407 | 1.350 | 0.676 |
| 79 | P(OCc1ccccc1)(OCC(N)C(O)=O)(O)=O | 0.869 | 2.548 | 0.118 | -0.474 | 1.820 | 1.677 |
| 80 | O(C(c1ccccc1)(c1ccccc1)c1ccccc1)CC(N)C(O)=O | 2.328 | -0.204 | 1.021 | -0.531 | 1.255 | -0.955 |
| 81 | O(C(C)(C)C)CC(N)C(O)=O | -0.387 | 0.325 | -1.828 | -0.137 | 0.518 | -0.253 |
| 82 | O(Cc1ccccc1)CC(N)C(O)=O | -0.007 | 0.515 | 0.724 | -0.376 | 1.626 | 1.049 |
| 83 | Brc1ccccc1COC(Oc1ccc(cc1)CC(N)C(O)=O)=O | 2.491 | -0.080 | 1.251 | -0.033 | 1.750 | 1.488 |
| 84 | O(CC=C)c1ccc(cc1)CC(N)C(O)=O | 0.524 | -0.358 | 0.290 | -0.412 | 1.620 | 1.413 |
| 85 | O(C(C)(C)C)c1ccc(cc1)CC(N)C(O)=O | 0.762 | -0.475 | -0.274 | -0.494 | 0.880 | 0.897 |
| 86 | OC(=O)C(N)Cc1c2c(n(c1)C)cccc2 | 0.299 | -0.602 | 1.127 | -0.496 | 1.349 | -0.074 |
| 87 | OC(=O)C(N)Cc1c2c(n(c1)C=O)cccc2 | 0.406 | 0.488 | 1.168 | -0.200 | 0.860 | 0.137 |
| 88 | Oc1c(cc(cc1C)CC(N)C(O)=O)C | 0.191 | 0.165 | 0.049 | -0.451 | -0.824 | 0.425 |
| 89 | O(C)c1ccc(cc1)CC(N)C(O)=O | 0.037 | -0.362 | 0.400 | -0.368 | 0.758 | 0.711 |
| 90 | O(C(C(N)C(O)=O)C)C | -0.849 | 0.625 | -1.360 | 0.046 | 0.411 | -0.978 |
| 91 | O(Cc1ccccc1)C(=O)NC(C(O)C)C(O)=O | 0.835 | 1.273 | 0.073 | -0.432 | 1.544 | 1.207 |
| 92 | O(C(C)(C)C)C(C(N)C(O)=O)C | -0.196 | 0.127 | -2.485 | -0.220 | 0.833 | -0.622 |
| 93 | P(OCc1ccccc1)(OC(C(N)C(O)=O)C)(O)=O | 1.097 | 1.837 | -0.284 | -0.535 | 1.222 | 1.108 |
| 94 | [SiH](OC(C(N)C(O)=O)C)(C)C | -0.253 | 0.027 | -2.188 | -0.091 | 1.477 | -0.202 |
| 95 | P(Oc1ccc(cc1)CC(N)C(O)=O)(OC)(OC)=O | 1.176 | 0.401 | -0.415 | -0.366 | 0.296 | 1.294 |
| 96 | OC(=O)CNC(=O)C(N)C | -0.638 | 0.698 | -1.066 | 0.028 | -1.215 | -0.083 |
| 97 | P(O)(O)(=O)CCCC(N)C(O)=O | -0.203 | 2.070 | -1.468 | 0.003 | -1.463 | 0.689 |
| 98 | OC(=O)C(N)CCC | -0.912 | -0.521 | -0.889 | 0.102 | 0.566 | -0.756 |
| 99 | OC(=O)C1Nc2c(C1)cccc2 | -0.469 | -1.129 | 1.612 | -0.225 | 0.843 | -0.947 |
| 100 | OC(=O)C1NC2CC1CC2 | -0.758 | -0.580 | 1.460 | 0.040 | 0.274 | -1.794 |
| 101 | S1CC(NC1=S)C(O)=O | -0.748 | -0.872 | 0.504 | 0.105 | 0.707 | -1.072 |
| 102 | O=C1NC(CC1)C(O)=O | -0.941 | 0.160 | 0.821 | 0.266 | -0.695 | -1.394 |
| 103 | OC(=O)C1NCc2[nH]c3c(c2C1)cccc3 | 0.251 | -0.437 | 2.214 | -0.477 | 1.052 | -0.436 |
| 104 | O=C1CC(NCC1)C(O)=O | -0.813 | 0.690 | 0.847 | 0.136 | -1.138 | -0.828 |
| 105 | O(CC(N)C(O)=O)CCN | -0.569 | 1.025 | -0.932 | 0.007 | -1.947 | 0.528 |
| 106 | O(C)c1cc2CC(C(O)=O)C(N)Cc2cc1OC | 0.802 | -0.691 | 0.777 | -0.441 | 0.169 | -0.057 |
| 107 | OC(=O)C1NCCNC1 | -0.938 | 0.603 | 0.989 | 0.067 | -1.235 | -1.017 |
| 108 | S(SCC(NC(=O)CCC(N)C(O)=O)C(=O)NCC(O)=O)CC(NC(=O)CCC(N)C(O)=O)C(=O)NCC(O)=O | 8.087 | 6.856 | -3.716 | 1.991 | -0.955 | -3.070 |
| 109 | OC(=O)C1NCC1 | -1.224 | -0.495 | 1.110 | 0.329 | -0.575 | -1.626 |
| 110 | OC(=O)CC1CCNCC1 | -0.663 | -0.631 | 0.839 | -0.023 | -1.148 | -0.360 |
| 111 | OC(=O)C(N)C(N)C(O)=O | -0.793 | 2.996 | -1.412 | -0.101 | -0.926 | -1.300 |
| 112 | OC(=O)C(N)CCCC(N)C(O)=O | -0.072 | 2.461 | -1.430 | -0.135 | -1.668 | 0.964 |
| 113 | S(CC(O)=O)c1ccc(N)cc1 | -0.308 | -0.738 | 0.663 | -0.354 | -0.480 | 0.690 |
| 114 | OC(=O)CN1CCNCC1 | -0.712 | 0.162 | 0.856 | 0.056 | -1.503 | -0.370 |
| 115 | Fc1ccc(cc1)C(N1CCNCC1)C(O)=O | 0.448 | 0.129 | 1.117 | -0.345 | -1.556 | -0.184 |
| 116 | OC(=O)C1(N)C2CC(C1)CC2 | -0.576 | -0.830 | 1.167 | -0.078 | 0.570 | -1.904 |
| 117 | OC(=O)C(N)(CC)c1ccccc1 | -0.241 | -0.491 | 0.060 | -0.470 | 0.022 | -0.727 |
| 118 | OC(=O)Cc1cc(N)ccc1 | -0.572 | -1.059 | 0.731 | -0.249 | -0.510 | -0.532 |
| 119 | OC(=O)CCCc1ccc(N)cc1 | -0.057 | -1.163 | 0.718 | -0.358 | -0.361 | 0.701 |
| 120 | S1CCC(N)(CC1)C(O)=O | -0.660 | -0.604 | 0.426 | -0.005 | 0.106 | -1.217 |
| 121 | OC(=O)CN1CCN(CC1)CCN | -0.057 | 0.725 | 0.508 | -0.118 | -1.721 | 0.903 |
| 122 | OC(=O)c1cnccc1N | -0.843 | -0.397 | 0.700 | 0.024 | -0.885 | -1.147 |
| 123 | OC(=O)C=Cc1ccc(N)cc1 | -0.352 | -1.333 | 0.796 | -0.113 | -0.334 | 0.234 |
| 124 | S1C2N(C(=O)C2N)C(C(O)=O)=C(C1)COC(=O)C | 0.717 | 1.407 | 0.715 | 0.085 | -0.045 | 0.040 |
| 125 | O(Cc1ccccc1)C(=O)CCCCCC(N)C(O)=O | 1.649 | 0.238 | 0.440 | -0.279 | 2.829 | 2.895 |
| 126 | OC(=O)C(N)CCCCCC | -0.206 | -0.488 | -1.223 | -0.197 | 1.655 | 1.178 |
| 127 | OC(=O)C(N)CC(=O)c1ccccc1N | 0.108 | 0.743 | 0.302 | -0.436 | 1.045 | 0.130 |
| 128 | OC(=O)C(N)CCCNC(=O)N | -0.224 | 1.579 | -1.062 | -0.194 | -1.562 | 0.974 |
| 129 | O(NC(N)=N)CCC(N)C(O)=O | -0.218 | 1.264 | -1.139 | -0.195 | -0.947 | 0.632 |
| 130 | S1CC(NC1)C(O)=O | -1.022 | -0.449 | 0.937 | 0.143 | 0.569 | -1.197 |
| 131 | OC(C(N)CC(C)C)CC(O)=O | -0.142 | 0.243 | -1.860 | -0.296 | 0.028 | 0.266 |
| 132 | OC(=O)C1NCc2c(C1)cccc2 | -0.303 | -0.380 | 1.682 | -0.361 | 1.224 | -0.326 |
| 133 | OC(=O)C1(N)CCNCC1 | -0.753 | 0.198 | 0.581 | -0.052 | -1.274 | -1.414 |
| 134 | OC(=O)C(N)N | -1.382 | 1.687 | -0.496 | 0.304 | -1.600 | -2.401 |
| 135 | O=C1N(CCC1N)CC(O)=O | -0.612 | 0.496 | 0.607 | 0.024 | 0.318 | -0.798 |
| 136 | OC(=O)C=1CNCCC=1 | -0.927 | -0.760 | 0.962 | 0.166 | -0.675 | -0.972 |
| 137 | OC(=O)CNN(C)C | -0.963 | 0.368 | -0.773 | 0.147 | -0.478 | -0.378 |
| 138 | OC(=O)CN(CC(=O)N)CC(O)=O | -0.161 | 2.028 | -2.009 | -0.038 | -1.399 | -0.122 |
| 139 | OC(=O)Cc1ccc(N)cc1 | -0.546 | -1.216 | 0.799 | -0.209 | -0.644 | -0.270 |
| 140 | O(C)c1cc(ccc1OC)CC(N)C(O)=O | 0.426 | -0.322 | -0.145 | -0.358 | 0.601 | 0.513 |
| 141 | FC(F)(F)c1ccc(cc1)CC(N)C(O)=O | 0.297 | -0.342 | 0.249 | -0.299 | -0.193 | 0.861 |
| 142 | OC(=O)C(N)Cc1ccc(N)cc1 | -0.189 | -0.325 | 0.547 | -0.398 | -0.086 | 0.142 |
| 143 | Clc1ccc(cc1)CC(N)C(O)=O | -0.140 | -0.643 | 0.448 | -0.421 | 1.437 | 0.511 |
| 144 | O(O)c1ccc(cc1)CC(N)C(O)=O | -0.040 | 0.253 | 0.573 | -0.231 | -1.060 | 0.722 |
| 145 | Ic1ccc(cc1)CC(N)C(O)=O | 0.064 | -0.781 | 0.230 | -0.442 | 1.632 | 0.952 |
| 146 | Oc1cc(O)c(O)cc1CC(N)C(O)=O | 0.098 | 1.188 | 0.170 | -0.339 | -1.241 | 0.193 |
| 147 | Fc1cc(cc(F)c1)CC(N)C(O)=O | -0.122 | -0.226 | 0.436 | -0.259 | -0.475 | 0.328 |
| 148 | OC(=O)C(N)Cc1ccc(cc1)C(C)(C)C | 0.555 | -0.861 | -0.214 | -0.502 | 1.922 | 0.557 |
| 149 | OC(=O)C(N)Cc1ccc(N=[N+]=[N-])cc1 | 0.124 | 1.071 | 0.670 | 5.979 | -1.220 | 1.484 |
| 150 | OC(=O)C(N)Cc1ccc(cc1)C(=O)c1ccccc1 | 1.226 | -0.126 | 1.306 | -0.297 | 1.370 | 1.027 |
| 151 | Clc1cc(Cl)ccc1CC(N)C(O)=O | 0.084 | -0.562 | 0.085 | -0.460 | 1.503 | 0.533 |
| 152 | FC(F)(F)c1ccccc1CC(N)C(O)=O | 0.201 | -0.224 | -0.085 | -0.422 | 0.477 | -0.146 |
| 153 | OC(=O)C(N)Cc1ccccc1C#N | -0.104 | 0.318 | 0.385 | -0.223 | 0.702 | 0.129 |
| 154 | Clc1ccccc1CC(N)C(O)=O | -0.187 | -0.590 | 0.314 | -0.430 | 1.049 | -0.049 |
| 155 | Fc1ccccc1CC(N)C(O)=O | -0.281 | -0.303 | 0.541 | -0.413 | 1.350 | -0.079 |
| 156 | OC(=O)C(N)Cc1ccccc1C | -0.203 | -0.646 | 0.314 | -0.430 | 1.085 | -0.158 |
| 157 | Clc1cc(ccc1Cl)CC(N)C(O)=O | 0.098 | -0.702 | 0.115 | -0.482 | 1.470 | 0.582 |
| 158 | Fc1cc(ccc1F)CC(N)C(O)=O | -0.105 | -0.356 | 0.480 | -0.349 | 0.270 | 0.387 |
| 159 | FC(F)(F)c1cc(ccc1)CC(N)C(O)=O | 0.241 | -0.306 | 0.146 | -0.351 | 0.497 | 0.456 |
| 160 | OC(=O)C(N)Cc1cc(ccc1)C#N | -0.061 | 0.213 | 0.517 | -0.182 | 0.428 | 0.526 |
| 161 | Clc1cc(ccc1)CC(N)C(O)=O | -0.164 | -0.637 | 0.388 | -0.416 | 1.264 | 0.194 |
| 162 | Fc1cc(ccc1)CC(N)C(O)=O | -0.258 | -0.527 | 0.549 | -0.271 | 0.398 | 0.152 |
| 163 | OC(=O)C(N)Cc1ccc(cc1)C#N | -0.044 | 0.263 | 0.646 | -0.187 | -0.214 | 0.909 |
| 164 | OC(=O)C(N)Cc1ccc([N+](=O)[O-])cc1 | 0.121 | 0.134 | 0.374 | 3.479 | -0.439 | 0.809 |
| 165 | Fc1c(CC(N)C(O)=O)c(F)c(F)c(F)c1F | 0.313 | 0.029 | -0.177 | -0.347 | -0.509 | 0.165 |
| 166 | P(O)(O)(=O)c1ccc(cc1)CC(N)C(O)=O | 0.371 | 1.890 | 0.220 | -0.305 | -1.090 | 0.940 |
| 167 | OC(=O)C(N)Cc1ccc(N=C(N)[NH-])cc1 | 0.420 | -0.291 | 0.131 | -0.072 | 0.731 | 0.381 |
| 168 | Fc1ccccc1CC(NC(=O)C)C(O)=O | 0.281 | 0.133 | -0.011 | -0.484 | 0.056 | -0.031 |
| 169 | O(C)c1cc(ccc1OC)CC(NC(=O)C)C(O)=O | 1.007 | 0.370 | -0.490 | -0.461 | -0.345 | 0.507 |
| 170 | Fc1cc(ccc1)CC(NC(=O)C)C(O)=O | 0.287 | 0.030 | 0.050 | -0.403 | -0.669 | 0.150 |
| 171 | Fc1ccc(cc1)CC(NC(=O)C)C(O)=O | 0.310 | 0.117 | 0.111 | -0.386 | -0.907 | 0.336 |
| 172 | OC(=O)C(N)(Cc1ccccc1)C | -0.201 | -0.577 | 0.296 | -0.419 | 1.073 | -0.314 |
| 173 | OC(=O)C(N)C(C)c1ccccc1 | -0.209 | -0.642 | 0.286 | -0.412 | 1.111 | -0.253 |
| 174 | Fc1ccc(cc1)CC(N)C(O)=O | -0.232 | -0.482 | 0.622 | -0.298 | -0.164 | 0.427 |
| 175 | Brc1ccc(cc1)CC(N)C(O)=O | -0.035 | -0.748 | 0.314 | -0.426 | 1.533 | 0.727 |
| 176 | OC(=O)C(Cc1ccccc1)CN | -0.194 | -0.662 | 0.512 | -0.393 | 0.568 | 0.044 |
| 177 | O(CC=C)C(=O)NCC(N)C(O)=O | -0.081 | 1.127 | -1.338 | -0.169 | 1.032 | 1.300 |
| 178 | OC(=O)C(N)CCCC | -0.691 | -0.561 | -1.001 | -0.004 | 0.726 | 0.035 |
| 179 | OC(=O)C(N)CCCCN=[N+]=[N-] | -0.205 | 1.333 | -0.854 | 6.105 | -1.316 | 1.934 |
| 180 | OC(=O)C(N)CCCN=[N+]=[N-] | -0.465 | 1.509 | -0.780 | 6.099 | -1.202 | 1.270 |
| 181 | OC(=O)C(N)CCCN | -0.740 | 0.492 | -0.784 | -0.029 | -0.864 | 0.109 |
| 182 | Clc1ccccc1COC(=O)NCCCC(N)C(O)=O | 1.578 | 0.446 | 0.248 | -0.364 | 2.415 | 2.439 |
| 183 | O(CC=C)C(=O)NCCCC(N)C(O)=O | 0.531 | 0.308 | -1.520 | -0.149 | 0.673 | 2.035 |
| 184 | OC(=O)C(NC)Cc1c2c([nH]c1)cccc2 | 0.341 | -0.271 | 1.153 | -0.560 | -0.100 | -0.032 |
| 185 | OC(=O)C(NC)C | -1.106 | -0.571 | -0.866 | 0.258 | -1.302 | -1.312 |
| 186 | OC(=O)C(NC)(C)C | -0.919 | -0.815 | -1.596 | 0.138 | -0.237 | -1.849 |
| 187 | OC(=O)C(NC)C(CC)C | -0.511 | -0.757 | -1.887 | -0.143 | -0.503 | -0.886 |
| 188 | OC(=O)C(NC)CO | -0.993 | 0.681 | -1.046 | 0.194 | -1.441 | -1.299 |
| 189 | O(Cc1ccccc1)C(C(NC)C(O)=O)C | 0.429 | 0.393 | 0.073 | -0.606 | 0.550 | 0.781 |
| 190 | O(C)c1ccc(cc1)CC(NC)C(O)=O | 0.270 | -0.324 | 0.099 | -0.484 | 0.164 | 0.643 |
| 191 | OC(=O)C(NC)C(C)C | -0.716 | -0.554 | -1.726 | 0.010 | -0.425 | -1.426 |
| 192 | OC(=O)C(NC)CCCC | -0.475 | -0.474 | -1.461 | -0.192 | -0.145 | 0.037 |
| 193 | O(Cc1ccccc1)CC(NC)C(O)=O | 0.234 | 0.646 | 0.414 | -0.504 | 0.689 | 0.981 |
| 194 | OC(=O)C(NC)CC(C)C | -0.486 | -0.714 | -1.771 | -0.152 | -0.171 | -0.759 |
| 195 | OC(=O)C(NC)Cc1ccccc1 | -0.195 | -0.304 | 0.432 | -0.431 | 0.190 | 0.017 |
| 196 | OC(=O)C(C(N)c1ccccc1)C | -0.193 | -0.694 | 0.259 | -0.453 | 1.110 | -0.200 |
| 197 | Clc1ccc(cc1)C(N)C(C(O)=O)C | 0.085 | -0.762 | 0.018 | -0.494 | 1.338 | 0.310 |
| 198 | OC(=O)C(N)CCCCCC(O)=O | 0.062 | 1.178 | -1.178 | -0.086 | -1.183 | 1.762 |
| 199 | OC(=O)C(C(C)C)CN | -0.716 | -0.948 | -1.588 | 0.009 | -0.658 | -1.195 |
| 200 | OC(=O)CC(N)CC#C | -0.781 | -0.623 | -0.765 | 0.035 | -0.245 | 0.153 |
| 201 | OC(=O)C(CN)CO | -0.999 | 0.681 | -0.905 | 0.185 | -1.379 | -1.115 |
| 202 | OC(=O)CC(N)CC | -0.913 | -0.757 | -0.808 | 0.145 | -1.048 | -0.528 |
| 203 | OC(=O)CCC(N)Cc1ccccc1 | 0.069 | -0.564 | 0.607 | -0.397 | 0.556 | 1.038 |
| 204 | OC(=O)CCC(N)CC(C)C | -0.238 | -0.740 | -1.525 | -0.148 | 0.156 | 0.662 |
| 205 | OC(=O)CCCCCCCCCCCNC | 1.270 | -0.384 | -1.775 | -0.429 | -0.179 | 4.027 |
| 206 | OC(=O)C(N)CCCCCCCCCCCCCC | 2.221 | -0.597 | -2.194 | -0.465 | 4.692 | 4.202 |
| 207 | OC(=O)C(N)CCCCC | -0.451 | -0.484 | -1.132 | -0.113 | 1.306 | 0.541 |
| 208 | OC(=O)CCCNC | -0.849 | -0.583 | -0.471 | 0.144 | -0.330 | 0.452 |
| 209 | OC(CC(O)=O)CN | -0.986 | 0.660 | -0.638 | 0.190 | -1.300 | -0.556 |
| 210 | OC(=O)CCC(=O)CN | -0.816 | 0.622 | -0.533 | 0.144 | -1.420 | 0.313 |
| 211 | OC(=O)CCCCN | -0.818 | -0.824 | -0.444 | 0.184 | -1.781 | 0.583 |
| 212 | OC(=O)CCCCCN | -0.604 | -0.722 | -0.573 | 0.060 | -1.025 | 1.091 |
| 213 | OC(=O)CCCCCCN | -0.350 | -0.774 | -0.787 | -0.021 | -1.702 | 1.724 |
| 214 | OC(=O)CCCCCCCN | -0.117 | -0.658 | -0.931 | -0.122 | -1.100 | 2.142 |
| 215 | O1CCOC1CCCC(N)C(O)=O | -0.131 | 1.043 | 0.704 | 0.055 | -0.543 | 1.050 |
| 216 | OC(=O)CCCCCCCCCCN | 0.697 | -0.649 | -1.446 | -0.312 | -1.353 | 3.524 |
| 217 | OC(=O)CCCCCCCCCCCN | 0.984 | -0.561 | -1.595 | -0.363 | -0.921 | 3.827 |
| 218 | OC(=O)CCCN | -1.048 | -0.760 | -0.264 | 0.305 | -0.988 | -0.156 |
| 219 | O(Cc1ccccc1)C(=O)NCCCCCC(O)=O | 1.440 | -0.223 | 0.455 | -0.294 | 1.045 | 2.859 |
| 220 | OC(=O)C1NCCCC1 | -0.890 | -0.326 | 0.867 | -0.008 | 0.666 | -1.129 |
| 221 | OC(=O)C1(NCCC1)CC=C | -0.536 | -0.440 | 0.232 | -0.225 | 0.588 | -1.292 |
| 222 | O=C1CC(NC1)C(O)=O | -0.984 | 0.498 | 1.008 | 0.319 | -1.834 | -0.903 |
| 223 | OC(=O)C1NCC=C1 | -1.121 | -0.498 | 1.088 | 0.154 | 0.374 | -1.349 |
| 224 | OC1CC(NC1)C(O)=O | -0.912 | -0.412 | 0.928 | 0.184 | -0.628 | -1.009 |
| 225 | OC(=O)C1NCC(C1)CC#C | -0.499 | -0.307 | 0.747 | -0.130 | 0.917 | 0.029 |
| 226 | Fc1cc(ccc1F)CC1CC(NC1)C(O)=O | 0.550 | -0.409 | 1.387 | -0.315 | 0.149 | 0.502 |
| 227 | FC(F)(F)c1ccccc1CC1CC(NC1)C(O)=O | 0.900 | -0.249 | 1.014 | -0.371 | 0.393 | 0.076 |
| 228 | FC(F)(F)c1ccc(cc1)CC1CC(NC1)C(O)=O | 0.970 | -0.253 | 1.282 | -0.293 | -0.010 | 0.810 |
| 229 | FC(F)(F)c1ccc(cc1)CC1(CCNC1)C(O)=O | 0.811 | -0.509 | 1.081 | -0.416 | -0.827 | 0.268 |
| 230 | OC(=O)C1(NCCC1)Cc1ccccc1 | 0.076 | -0.626 | 1.227 | -0.535 | -0.881 | -0.804 |
| 231 | Fc1ccc(cc1)CC1(NCCC1)C(O)=O | 0.248 | -0.464 | 1.221 | -0.461 | -1.134 | -0.318 |
| 232 | O(C)C1CC(NC1)C(O)=O | -0.718 | 0.089 | 0.751 | 0.092 | -0.880 | -0.416 |
| 233 | OC(=O)C1(NCCC1)Cc1c2c(ccc1)cccc2 | 0.821 | -0.818 | 1.755 | -0.635 | -0.740 | -0.936 |
| 234 | OC(=O)C1(NCCC1)CC#C | -0.531 | -0.506 | 0.287 | -0.148 | 0.508 | -1.270 |
| 235 | Clc1ccccc1CC1(NCCC1)C(O)=O | 0.301 | -0.775 | 0.931 | -0.606 | -0.544 | -0.805 |
| 236 | Brc1ccccc1CC1(NCCC1)C(O)=O | 0.385 | -0.857 | 0.809 | -0.632 | -0.429 | -0.748 |
| 237 | OC(=O)C1(NCCC1)Cc1ccc(cc1)C | 0.338 | -0.681 | 1.063 | -0.598 | -0.800 | -0.422 |
| 238 | Brc1ccc(cc1)CC1(NCCC1)C(O)=O | 0.434 | -0.701 | 0.949 | -0.607 | -0.564 | 0.014 |
| 239 | OC(=O)C1(NCCC1)CCC | -0.455 | -0.705 | 0.089 | -0.183 | -0.473 | -1.206 |
| 240 | OC(=O)C1NCC(N)C1 | -0.887 | 0.251 | 0.856 | 0.063 | -1.625 | -1.165 |
| 241 | OC(=O)C1NCC(N=[N+]=[N-])C1 | -0.605 | 1.166 | 0.920 | 6.242 | -1.825 | 0.196 |
| 242 | OC(=O)C1(NCCC1)C | -0.856 | -0.730 | 0.403 | 0.094 | -0.910 | -1.894 |
| 243 | OC1CCNC1C(O)=O | -0.966 | 0.488 | 0.787 | 0.080 | -0.154 | -1.469 |
| 244 | OC1CC(N(C1)C(=O)C)C(O)=O | -0.483 | 0.391 | 0.265 | 0.004 | 0.240 | -0.951 |
| 245 | S(SC(C(N)C(O)=O)(C)C)C(C(N)C(O)=O)(C)C | 1.056 | 1.904 | -3.599 | -0.503 | -0.241 | 0.925 |
| 246 | SC(C(N)C(O)=O)(C)C | -0.693 | -0.567 | -1.939 | 0.212 | 0.086 | -1.581 |
| 247 | S(Cc1ccc(OC)cc1)C(C(N)C(O)=O)(C)C | 1.034 | -0.584 | -0.341 | -0.518 | 0.722 | 1.428 |
| 248 | S(C(C(N)C(O)=O)(C)C)C | -0.535 | -0.633 | -2.150 | -0.114 | 0.003 | -1.054 |
| 249 | SC(C(NC(=O)C)C(O)=O)(C)C | -0.210 | 0.148 | -2.543 | -0.047 | -0.281 | -0.877 |
| 250 | OC(C(N)C(O)=O)c1ccccc1 | -0.314 | 0.534 | 0.455 | -0.365 | 0.835 | -0.032 |
| 251 | OC(CN)C(O)=O | -1.209 | 0.942 | -0.590 | 0.230 | -1.003 | -1.285 |
| 252 | OC(C(NC(=O)c1ccccc1)c1ccccc1)C(O)=O | 1.211 | 0.699 | 0.959 | -0.413 | -0.591 | -0.269 |
| 253 | Oc1ccc(cc1N)CC(N)C(O)=O | -0.028 | 0.360 | 0.349 | -0.386 | -0.800 | 0.037 |
| 254 | Clc1cc(ccc1O)CC(N)C(O)=O | 0.020 | -0.215 | 0.246 | -0.357 | 0.070 | 0.404 |
| 255 | Oc1ccc(cc1[N+](=O)[O-])CC(N)C(O)=O | 0.271 | 0.740 | 0.062 | 3.119 | -0.829 | 0.434 |
| 256 | Oc1ccccc1CC(N)C(O)=O | -0.271 | 0.091 | 0.481 | -0.322 | 0.798 | -0.147 |
| 257 | Oc1cc(ccc1)CC(N)C(O)=O | -0.247 | -0.005 | 0.546 | -0.308 | 0.222 | 0.077 |
| 258 | Ic1cc(cc(I)c1O)CC(N)C(O)=O | 0.544 | 0.181 | -0.554 | -0.586 | -0.229 | 0.957 |
| 259 | Fc1cc2c([nH]cc2CC(N)C(O)=O)cc1 | 0.245 | -0.382 | 1.320 | -0.406 | 0.272 | 0.024 |
| 260 | O(C)c1cc2c([nH]cc2CC(N)C(O)=O)cc1 | 0.525 | -0.292 | 1.124 | -0.472 | 0.079 | 0.175 |
| 261 | OC(=O)C(N)Cc1c2cc(ccc2[nH]c1)C | 0.343 | -0.670 | 1.142 | -0.552 | 1.011 | 0.008 |
| 262 | Oc1cc2c([nH]cc2CC(N)C(O)=O)cc1 | 0.262 | -0.011 | 1.279 | -0.426 | -0.143 | -0.006 |
| 263 | OC(=O)C(N)Cc1c2c([nH]c1)cc(cc2)C | 0.342 | -0.518 | 1.182 | -0.496 | 1.139 | 0.158 |
| 264 | OC(=O)C1(N)CCc2c(C1)cccc2 | -0.109 | -0.618 | 1.390 | -0.429 | 1.130 | -0.623 |
| 265 | O(C)c1c(N)c(cc(OC)c1OC)C(O)=O | 0.326 | -0.417 | -0.760 | -0.360 | -0.696 | -0.631 |
| 266 | Brc1cc(Br)cc(C(O)=O)c1N | -0.157 | -1.407 | -0.383 | -0.393 | 1.010 | -0.727 |
| 267 | FC(F)(F)c1cccc(C(O)=O)c1N | -0.205 | -0.831 | 0.004 | -0.195 | -0.287 | -0.639 |
| 268 | Clc1cccc(C(O)=O)c1N | -0.558 | -1.379 | 0.244 | -0.213 | 0.821 | -1.068 |
| 269 | Brc1cc(cc(C(O)=O)c1N)C | -0.260 | -1.428 | -0.166 | -0.336 | 0.962 | -0.880 |
| 270 | OC(=O)c1cccc(C)c1N | -0.583 | -1.368 | 0.278 | -0.217 | 0.738 | -1.274 |
| 271 | OC(=O)c1cccc([N+](=O)[O-])c1N | -0.331 | -0.325 | 0.060 | 3.472 | -0.381 | -0.809 |
| 272 | O(C)c1cc(C(O)=O)c(N)cc1OC | -0.002 | -0.628 | -0.253 | -0.285 | 0.110 | -0.580 |
| 273 | Fc1cc(N)c(cc1)C(O)=O | -0.612 | -1.220 | 0.500 | -0.075 | -0.976 | -0.770 |
| 274 | Clc1cc(N)c(cc1)C(O)=O | -0.522 | -1.373 | 0.325 | -0.219 | 0.886 | -0.662 |
| 275 | Brc1cc(N)c(cc1)C(O)=O | -0.417 | -1.485 | 0.203 | -0.225 | 0.995 | -0.415 |
| 276 | Fc1cc(C(O)=O)c(N)cc1 | -0.639 | -1.159 | 0.479 | -0.122 | -0.442 | -1.035 |
| 277 | Clc1cc(C(O)=O)c(N)c(c1)C | -0.357 | -1.354 | -0.050 | -0.327 | 0.939 | -1.123 |
| 278 | Clc1cc(C(O)=O)c(N)cc1 | -0.545 | -1.346 | 0.286 | -0.244 | 0.873 | -1.008 |
| 279 | Brc1cc(C(O)=O)c(N)cc1 | -0.450 | -1.399 | 0.172 | -0.261 | 1.028 | -0.756 |
| 280 | OC(=O)c1cc(ccc1N)C | -0.571 | -1.345 | 0.330 | -0.239 | 0.734 | -1.171 |
| 281 | OC(=O)c1cc([N+](=O)[O-])ccc1N | -0.321 | -0.261 | 0.157 | 3.509 | -0.912 | -0.703 |
| 282 | Oc1cc(C(O)=O)c(N)cc1 | -0.632 | -0.537 | 0.450 | -0.188 | -0.621 | -1.166 |
| 283 | Fc1cccc(N)c1C(O)=O | -0.661 | -0.943 | 0.365 | -0.184 | 0.265 | -1.420 |
| 284 | Clc1cccc(N)c1C(O)=O | -0.561 | -1.486 | 0.173 | -0.213 | 0.746 | -1.407 |
| 285 | OC(=O)c1c(cccc1N)C | -0.579 | -1.426 | 0.186 | -0.221 | 0.869 | -1.566 |
| 286 | OC(=O)c1ccccc1C(=O)c1ccccc1N | 0.618 | -0.464 | 0.900 | 0.113 | -1.057 | -1.253 |
| 287 | OC(=O)c1ccc(cc1N)C(O)=O | -0.288 | 0.381 | 0.338 | -0.061 | -2.098 | -0.388 |
| 288 | OC(=O)Cc1ccccc1Nc1ccccc1 | 0.537 | -1.200 | 1.201 | -0.315 | 0.007 | -0.225 |
| 289 | OC(=O)c1cc(N)c(N)cc1 | -0.595 | -0.681 | 0.509 | -0.207 | -1.346 | -0.954 |
| 290 | OC(=O)c1cc(N)cc(N)c1 | -0.603 | -0.690 | 0.464 | -0.256 | -1.543 | -1.260 |
| 291 | Clc1cc(Cl)cc(C(O)=O)c1N | -0.326 | -1.365 | -0.132 | -0.331 | 0.985 | -0.979 |
| 292 | OC(=O)c1cc(cc(C)c1N)C | -0.372 | -1.408 | -0.012 | -0.316 | 0.873 | -1.234 |
| 293 | OC(=O)CCc1ccc(N)cc1 | -0.304 | -1.206 | 0.806 | -0.309 | -0.679 | 0.369 |
| 294 | FNCc1cc(ccc1)C(O)=O | -0.486 | -0.143 | 0.768 | -0.167 | -0.248 | 0.167 |
| 295 | OC(=O)c1cc(ccc1)CN | -0.603 | -0.573 | 0.721 | -0.201 | -0.267 | -0.387 |
| 296 | Brc1c(cccc1N)C(O)=O | -0.509 | -1.522 | 0.191 | -0.250 | -0.512 | -0.976 |
| 297 | OC(=O)c1cc(NC(=O)C)ccc1 | -0.253 | -0.481 | 0.428 | -0.161 | -0.153 | -0.096 |
| 298 | Clc1c(cc(Cl)cc1N)C(O)=O | -0.346 | -1.604 | -0.068 | -0.316 | -0.244 | -0.859 |
| 299 | Clc1c(cccc1N)C(O)=O | -0.574 | -1.585 | 0.286 | -0.197 | -0.480 | -1.076 |
| 300 | OC(=O)c1cccc(N)c1C | -0.589 | -1.601 | 0.318 | -0.188 | -0.538 | -1.167 |
| 301 | O(C)c1c(cccc1N)C(O)=O | -0.455 | -0.960 | 0.126 | -0.248 | -0.411 | -1.135 |
| 302 | OC(=O)c1cc2c(cc1N)cccc2 | -0.109 | -1.372 | 1.274 | -0.380 | 0.953 | -0.709 |
| 303 | Clc1ccc(cc1N)C(O)=O | -0.534 | -1.283 | 0.403 | -0.197 | 0.259 | -0.546 |
| 304 | Brc1ccc(cc1N)C(O)=O | -0.434 | -1.410 | 0.284 | -0.206 | 0.371 | -0.330 |
| 305 | OC(=O)c1cc(N)c(cc1)C | -0.568 | -1.258 | 0.461 | -0.214 | 0.088 | -0.760 |
| 306 | O(C)c1ccc(cc1N)C(O)=O | -0.381 | -0.971 | 0.310 | -0.117 | -0.465 | -0.483 |
| 307 | Oc1ccc(cc1N)C(O)=O | -0.615 | -0.512 | 0.572 | -0.127 | -1.690 | -0.739 |
| 308 | FC(F)(F)c1cc(cc(N)c1)C(O)=O | -0.193 | -0.628 | 0.105 | -0.244 | -0.971 | -0.508 |
| 309 | Brc1cc(cc(N)c1)C(O)=O | -0.454 | -1.295 | 0.214 | -0.244 | 0.225 | -0.726 |
| 310 | O(C)c1cc(cc(N)c1)C(O)=O | -0.404 | -1.000 | 0.276 | -0.199 | -0.709 | -0.698 |
| 311 | Oc1c(cccc1N)C(O)=O | -0.664 | 0.005 | 0.448 | -0.198 | -0.028 | -1.131 |
| 312 | OC(=O)c1c(cccc1N)C(O)=O | -0.330 | 0.061 | 0.074 | -0.117 | -0.389 | -1.333 |
| 313 | OC(=O)c1cc(N)ccc1 | -0.778 | -1.184 | 0.744 | -0.124 | -0.296 | -0.997 |
| 314 | OC(=O)c1cc(NC)ccc1 | -0.568 | -1.557 | 0.600 | -0.133 | -0.146 | -0.492 |
| 315 | Oc1cccc(C(O)=O)c1N | -0.634 | -0.641 | 0.391 | -0.125 | -0.523 | -1.225 |
| 316 | Fc1cc(C(O)=O)c(N)cc1F | -0.493 | -1.175 | 0.296 | -0.152 | -0.711 | -0.826 |
| 317 | OC(=O)c1ccc(cc1)CCN | -0.312 | -0.908 | 0.722 | -0.161 | -1.842 | 0.558 |
| 318 | FNNc1ccc(cc1)C(O)=O | -0.483 | 0.027 | 0.877 | -0.125 | -1.308 | 0.426 |
| 319 | OC(=O)c1ccc(NCCCC)cc1 | 0.211 | -1.524 | 0.443 | -0.287 | 1.413 | 1.182 |
| 320 | OC(=O)c1ccc(cc1)CN | -0.570 | -0.939 | 0.830 | -0.140 | -0.858 | 0.005 |
| 321 | OC(=O)c1ccc(NC(=O)C)cc1C | -0.019 | -0.903 | 0.226 | -0.176 | -1.175 | 0.322 |
| 322 | OC(=O)c1ccc(NC(=O)C)cc1 | -0.222 | -0.779 | 0.601 | -0.066 | -0.795 | 0.507 |
| 323 | Fc1cc(N)ccc1C(O)=O | -0.640 | -1.051 | 0.575 | -0.161 | -0.708 | -0.625 |
| 324 | OC(=O)c1ccc(N)cc1C | -0.564 | -1.571 | 0.410 | -0.187 | -0.939 | -0.761 |
| 325 | O(C)c1cc(N)ccc1C(O)=O | -0.432 | -1.009 | 0.244 | -0.233 | -0.862 | -0.719 |
| 326 | Clc1cc(cc(Cl)c1N)C(O)=O | -0.323 | -1.486 | -0.049 | -0.257 | -0.451 | -0.670 |
| 327 | Clc1cc(ccc1N)C(O)=O | -0.537 | -1.416 | 0.393 | -0.179 | -0.665 | -0.595 |
| 328 | Brc1cc(ccc1N)C(O)=O | -0.446 | -1.526 | 0.278 | -0.199 | -0.398 | -0.418 |
| 329 | OC(=O)c1cc(C)c(N)cc1 | -0.566 | -1.508 | 0.469 | -0.183 | -0.721 | -0.680 |
| 330 | O(C)c1cc(ccc1N)C(O)=O | -0.437 | -0.766 | 0.277 | -0.246 | -1.636 | -0.721 |
| 331 | OC(=O)c1cc([N+](=O)[O-])c(N)cc1 | -0.330 | -0.226 | 0.153 | 3.327 | -1.528 | -0.646 |
| 332 | Oc1cc(ccc1N)C(O)=O | -0.624 | -0.438 | 0.563 | -0.193 | -1.515 | -0.789 |
| 333 | Clc1cc(C(O)=O)c(OC)cc1N | -0.206 | -1.107 | -0.098 | -0.303 | -0.606 | -0.629 |
| 334 | Oc1cc(N)ccc1C(O)=O | -0.606 | -0.785 | 0.532 | -0.128 | -1.015 | -0.688 |
| 335 | OC(=O)c1cc(N)ccc1C(O)=O | -0.321 | 0.225 | 0.201 | -0.119 | -1.042 | -0.878 |
| 336 | OC(=O)c1ccc(NC)cc1 | -0.532 | -1.604 | 0.664 | -0.077 | 0.347 | -0.227 |
| 337 | OC(=O)c1ccc([N+](=O)[O-])cc1N | -0.292 | -0.207 | 0.256 | 3.593 | -1.005 | -0.245 |
| 338 | Ic1c(C(O)=O)c(I)c(N)c(I)c1C(O)=O | 0.800 | -0.627 | -1.633 | -0.534 | -1.345 | -0.398 |
| 339 | Clc1ccc(N)cc1C(O)=O | -0.557 | -1.324 | 0.317 | -0.253 | 0.018 | -0.985 |
| 340 | Brc1ccc(N)cc1C(O)=O | -0.472 | -1.352 | 0.206 | -0.285 | 0.245 | -0.815 |
| 341 | OC(=O)c1cc(N)ccc1C | -0.580 | -1.321 | 0.356 | -0.247 | -0.023 | -1.156 |
| 342 | O(C)c1ccc(N)cc1C(O)=O | -0.434 | -0.779 | 0.226 | -0.279 | -0.314 | -0.891 |
| 343 | OC(=O)c1cc(N)ccc1[N+](=O)[O-] | -0.324 | -0.497 | 0.065 | 3.356 | -0.855 | -0.910 |
| 344 | Oc1ccc(N)cc1C(O)=O | -0.628 | -0.561 | 0.497 | -0.173 | -1.047 | -1.039 |
| 345 | OC(=O)c1cc(cc(N)c1)C(O)=O | -0.327 | 0.803 | 0.265 | -0.146 | -1.888 | -0.724 |
| 346 | OC(=O)c1cc2c(cc(N)cc2)cc1 | -0.080 | -1.439 | 1.442 | -0.350 | -0.847 | -0.159 |
| 347 | OC(=O)c1ccccc1N | -0.782 | -1.273 | 0.603 | -0.114 | 0.399 | -1.311 |
| 348 | OC(=O)c1ccc(N)cc1 | -0.760 | -1.371 | 0.838 | -0.084 | -1.010 | -0.553 |
| 349 | FNCc1ccc(cc1)C(O)=O | -0.448 | -0.414 | 0.889 | -0.097 | -1.013 | 0.654 |
| 350 | FC(F)(F)C(=O)NCCCCC(NC(CCc1ccccc1)C(OCC)=O)C(O)=O | 4.155 | 2.222 | -0.537 | 0.112 | 1.262 | 1.379 |
| 351 | OC(CCC(N)C(O)=O)CN | -0.380 | 1.391 | -1.228 | -0.121 | -1.896 | 0.447 |
| 352 | FNC(CCCN)C(O)=O | -0.612 | 0.761 | -1.075 | -0.047 | -1.358 | 0.270 |
| 353 | FC(F)(F)C(CC(N)C(O)=O)C | -0.398 | 0.146 | -1.757 | -0.078 | 0.013 | -0.456 |
| 354 | OC(=O)C(N)CC(C)=C | -0.778 | -0.654 | -1.188 | 0.023 | 0.679 | -0.808 |
| 355 | FNC(C(C)(C)C)C(O)=O | -0.616 | -0.707 | -2.109 | -0.025 | 0.435 | -1.566 |
| 356 | O(C)c1cccc(C(O)=O)c1N | -0.425 | -0.922 | 0.194 | -0.219 | 0.298 | -0.772 |
| 357 | OC(=O)C(NC(=O)CC1c2c(NC1)cccc2)C(CC)C | 1.561 | -0.298 | 0.796 | -0.460 | -1.583 | 0.221 |
| 358 | OC(=O)CCNC(=N)[NH-] | -0.758 | -1.216 | -0.790 | 0.404 | 0.139 | -0.401 |
| 359 | OC(=O)CCCNC(=N)[NH-] | -0.531 | -0.984 | -0.898 | 0.283 | 0.667 | 0.102 |
| 360 | OC(=O)C(NC(=N)[NH-])N | -0.903 | 0.224 | -1.084 | 0.384 | -0.149 | -1.536 |
| 361 | OC(C(N)Cc1ccccc1)C(=O)NC(CC(C)C)C(O)=O | 1.911 | 1.531 | -0.781 | -0.655 | -0.338 | 0.907 |
| 362 | OC(=O)C(N)C(C)(C)C | -0.740 | -0.941 | -1.884 | 0.032 | 0.572 | -1.779 |
| 363 | OC(=O)C(N)CCC#C | -0.776 | -0.329 | -0.749 | -0.020 | 0.968 | -0.036 |
| 364 | OC(=O)C(N)CCO | -1.017 | 0.809 | -0.665 | 0.165 | -0.656 | -0.650 |
| 365 | SCCC(N)C(O)=O | -0.875 | -0.168 | -0.893 | 0.277 | 0.487 | -0.484 |
| 366 | S(SCCC(N)C(O)=O)CCC(N)C(O)=O | 0.847 | 2.448 | -2.089 | -0.299 | -1.065 | 2.545 |
| 367 | OC(=O)C(N)CCCCNC(=N)[NH-] | 0.081 | -0.175 | -1.386 | 0.059 | 1.157 | 0.904 |
| 368 | O=C1NC(CC1)CC(O)=O | -0.737 | 0.010 | 0.839 | 0.176 | -1.557 | -0.650 |
| 369 | FNC(CCc1ccccc1)C(O)=O | -0.023 | -0.001 | 0.614 | -0.401 | 1.609 | 0.772 |
| 370 | OC(=O)c1ccccc1Nc1ccccc1 | 0.304 | -1.663 | 1.206 | -0.247 | -0.135 | -0.818 |
| 371 | OC(=O)c1cccc(N)c1N | -0.604 | -0.818 | 0.321 | -0.218 | -0.324 | -1.496 |
| 372 | OC(=O)c1ccccc1CN | -0.624 | -0.856 | 0.528 | -0.219 | 0.580 | -0.997 |
| 373 | Fc1c(C(O)=O)c(N)c(F)c(F)c1F | -0.245 | -0.730 | -0.232 | -0.194 | -0.980 | -1.124 |
| 374 | Ic1cc(C(O)=O)c(N)cc1 | -0.371 | -1.389 | 0.079 | -0.298 | 1.162 | -0.562 |
| 375 | FNC(CCOC(c1ccccc1)(c1ccccc1)c1ccccc1)C(O)=O | 2.898 | -0.293 | 1.167 | -0.402 | 1.473 | -0.625 |
| 376 | OC(=O)C(N)CCc1ccccc1 | -0.144 | -0.562 | 0.658 | -0.364 | 1.679 | 0.476 |
| 377 | O(C(C)(C)C)C(=O)CC(N)C(O)=O | -0.070 | 0.331 | -2.048 | -0.133 | 0.893 | 0.181 |
| 378 | OC(=O)C(N)CCCC(O)=O | -0.449 | 1.493 | -0.969 | 0.067 | -1.601 | 0.716 |
| 379 | OC(=O)C(NC(=O)c1cc([N+](=O)[O-])cc([N+](=O)[O-])c1)CC(C)C | 1.865 | 1.834 | -1.012 | 7.563 | -0.578 | 0.292 |
| 380 | OC(=O)CC(N)Cc1ccc(cc1)-c1ccccc1 | 1.013 | -0.682 | 1.439 | -0.455 | 1.020 | 1.080 |
| 381 | OC(=O)C1CC(N)CC1 | -0.839 | -0.909 | 0.817 | 0.107 | -1.329 | -0.965 |
| 382 | OC(C(N)Cc1ccccc1)C(O)=O | -0.080 | 0.692 | 0.456 | -0.478 | 1.070 | 0.200 |
| 383 | OC(=O)C(CN)C | -1.116 | -0.558 | -0.782 | 0.185 | 0.136 | -1.381 |
| 384 | OC(=O)C(CN)c1ccccc1 | -0.406 | -0.543 | 0.464 | -0.354 | 1.059 | -0.571 |
| 385 | O(C)c1ccc(cc1)C(N)CC(O)=O | 0.024 | -0.407 | 0.396 | -0.321 | 0.333 | 0.734 |
| 386 | FNC(Cc1cccnc1)CC(O)=O | -0.108 | 0.440 | 0.687 | -0.273 | -0.551 | 0.899 |
| 387 | Fc1ccc(cc1)C(NF)CC(O)=O | -0.113 | -0.484 | 0.503 | -0.236 | -1.169 | 0.630 |
| 388 | FC(F)(F)c1ccc(cc1)C(N)CC(O)=O | 0.278 | -0.670 | 0.274 | -0.264 | -1.189 | 0.960 |
| 389 | FNCC(C(O)=O)C | -1.015 | -0.362 | -0.721 | 0.216 | -1.226 | -0.542 |
| 390 | OC(=O)CC(N)CC=C | -0.760 | -0.540 | -0.884 | 0.037 | -0.859 | 0.004 |
| 391 | OC(=O)CC(N)CCc1ccccc1 | 0.060 | -0.517 | 0.675 | -0.397 | 0.694 | 1.161 |
| 392 | OC(=O)CC(N)c1ccccc1C | -0.221 | -0.351 | 0.277 | -0.445 | 0.353 | -0.171 |
| 393 | OC(=O)CC(N)Cc1ccccc1C#N | 0.081 | 0.276 | 0.437 | -0.258 | 0.015 | 0.459 |
| 394 | O(C)c1cc(ccc1OC)C(N)CC(O)=O | 0.442 | -0.276 | -0.161 | -0.366 | 0.357 | 0.674 |
| 395 | O(C)c1cc(ccc1)C(N)CC(O)=O | -0.020 | -0.645 | 0.337 | -0.338 | -0.799 | 0.554 |
| 396 | OC(=O)CC(N)c1cc(ccc1)C | -0.220 | -0.671 | 0.439 | -0.370 | -0.126 | 0.208 |
| 397 | OC(=O)CC(N)c1cc(ccc1)C#N | -0.075 | -0.021 | 0.532 | -0.181 | -0.787 | 0.657 |
| 398 | OC(=O)CC(N)Cc1ccncc1 | -0.235 | -0.022 | 0.779 | -0.265 | -1.381 | 0.719 |
| 399 | Ic1ccc(cc1)CC(N)CC(O)=O | 0.206 | -0.568 | 0.252 | -0.495 | 0.544 | 1.214 |
| 400 | OC(=O)CC(N)Cc1ccc(cc1)C | 0.007 | -0.553 | 0.506 | -0.448 | 0.235 | 0.616 |
| 401 | OC(=O)CC(N)c1ccc(cc1)C | -0.180 | -0.908 | 0.474 | -0.360 | 0.006 | 0.407 |
| 402 | Fc1ccc(cc1)C(N)CC(O)=O | -0.229 | -0.839 | 0.583 | -0.226 | -1.615 | 0.467 |
| 403 | Clc1ccc(cc1)C(N)CC(O)=O | -0.172 | -0.810 | 0.462 | -0.365 | 0.206 | 0.582 |
| 404 | Clc1ccc(cc1)CC(N)CC(O)=O | 0.023 | -0.536 | 0.472 | -0.467 | 0.400 | 0.772 |
| 405 | OC(=O)CC(N)c1ccc(cc1)C#N | -0.063 | 0.119 | 0.647 | -0.138 | -1.247 | 0.938 |
| 406 | OC(=O)CC(N)Cc1ccc(cc1)C#N | 0.148 | 0.346 | 0.664 | -0.226 | -0.705 | 1.188 |
| 407 | Brc1ccc(cc1)C(N)CC(O)=O | -0.059 | -1.048 | 0.326 | -0.379 | 0.460 | 0.813 |
| 408 | Oc1ccc(cc1)C(N)CC(O)=O | -0.237 | -0.248 | 0.603 | -0.269 | -1.677 | 0.451 |
| 409 | OC(=O)CC(N)C(c1ccccc1)c1ccccc1 | 0.818 | -0.843 | 0.874 | -0.595 | 0.360 | -0.276 |
| 410 | OC(=O)CC(N)Cc1ccccc1C | 0.053 | -0.589 | 0.234 | -0.427 | 1.237 | 0.377 |
| 411 | Fc1cc(ccc1F)CC(N)CC(O)=O | 0.101 | -0.366 | 0.453 | -0.347 | -1.085 | 0.622 |
| 412 | FC(F)(F)c1cc(ccc1)CC(N)CC(O)=O | 0.445 | -0.096 | 0.194 | -0.410 | -0.596 | 0.662 |
| 413 | OC(=O)CC(N)Cc1cc(ccc1)C#N | 0.117 | 0.333 | 0.555 | -0.237 | -0.409 | 0.843 |
| 414 | OC(=O)CC(N)Cc1cc(ccc1)C | 0.046 | -0.696 | 0.393 | -0.454 | 0.474 | 0.647 |
| 415 | OC(=O)C1CCNC1 | -1.066 | -0.745 | 1.060 | 0.210 | -0.926 | -1.277 |
| 416 | OC(=O)CC(N)CC=Cc1ccccc1 | 0.222 | -0.578 | 0.694 | -0.384 | 0.683 | 1.259 |
| 417 | FC(F)(F)c1ccc(cc1)CC(N)CC(O)=O | 0.502 | -0.217 | 0.280 | -0.352 | -1.045 | 1.023 |
| 418 | FC(F)(F)c1ccccc1CC(N)CC(O)=O | 0.449 | -0.245 | -0.062 | -0.473 | 0.105 | 0.325 |
| 419 | FC(F)(F)c1ccccc1C(N)CC(O)=O | 0.208 | -0.150 | -0.104 | -0.428 | 0.384 | -0.008 |
| 420 | FC(F)(F)c1cc(ccc1)C(N)CC(O)=O | 0.262 | -0.856 | 0.054 | -0.216 | -0.405 | 0.102 |
| 421 | OC(=O)Cc1ccccc1CN | -0.414 | -0.671 | 0.450 | -0.322 | -0.085 | -0.521 |
| 422 | Brc1cc(ccc1)C(N)CC(O)=O | -0.046 | -0.828 | 0.220 | -0.417 | 1.523 | 0.524 |
| 423 | OC(=O)CC(N)CO | -0.992 | 0.804 | -0.675 | 0.185 | -1.321 | -0.574 |
| 424 | OC1CC(NC1)CC(O)=O | -0.739 | 0.217 | 0.912 | 0.063 | -1.811 | -0.434 |
| 425 | OC(=O)CC(N)Cc1[nH]c2c(c1)cccc2 | 0.277 | -0.221 | 1.537 | -0.511 | 0.515 | 0.304 |
| 426 | OC(C(N)CC(O)=O)C | -0.834 | 0.784 | -1.023 | 0.030 | -1.140 | -0.552 |
| 427 | OC(=O)CC(N)CCC(O)=O | -0.449 | 1.242 | -1.040 | 0.061 | -1.970 | 0.801 |
| 428 | Oc1ccc(cc1)CC(N)CC(O)=O | -0.046 | -0.003 | 0.626 | -0.349 | -0.800 | 0.639 |
| 429 | O(Cc1ccccc1)C(=O)CC(N)CC(O)=O | 0.645 | 0.433 | 0.534 | -0.288 | 0.896 | 1.905 |
| 430 | S(=O)(=O)(NC(NCCCC(N)CC(O)=O)=[N-])c1ccc(O)cc1 | 2.290 | 1.764 | -0.099 | -0.292 | -0.253 | 2.134 |
| 431 | O(Cc1ccccc1)C1CC(NC1)CC(O)=O | 0.609 | -0.084 | 1.696 | -0.398 | 0.706 | 1.079 |
| 432 | O(Cc1ccccc1)C(C(N)CC(O)=O)C | 0.484 | 0.274 | 0.165 | -0.546 | 1.268 | 1.060 |
| 433 | O(Cc1ccccc1)c1ccc(cc1)CC(N)CC(O)=O | 1.600 | -0.475 | 1.506 | -0.394 | 1.115 | 1.516 |
| 434 | OC(=O)CC(N)CCCN | -0.511 | 0.255 | -0.940 | -0.079 | -1.770 | 0.822 |
| 435 | O(C(C)(C)C)C(=O)CC(N)CC(O)=O | 0.203 | 0.242 | -2.161 | -0.111 | 0.776 | 0.929 |
| 436 | OC(=O)CC(N)CC(=O)N | -0.656 | 1.234 | -1.084 | -0.018 | -1.936 | -0.220 |
| 437 | OC(=O)CC(N)CC(=O)NC(c1ccccc1)(c1ccccc1)c1ccccc1 | 3.147 | -0.604 | 1.255 | -0.374 | 0.766 | -1.000 |
| 438 | OC(=O)CC(N)Cc1cc2c(cc1)cccc2 | 0.508 | -0.800 | 1.502 | -0.469 | 0.599 | 0.495 |
| 439 | S(=O)(=O)(NC(=O)NCCCC(N)CC(O)=O)c1c(C)c(c2c(CCC(C)C(OC2)C)c1C)C | 5.484 | -0.511 | 0.646 | 0.566 | -0.874 | -1.826 |
| 440 | OC(=O)CC(N)CCC(=O)N | -0.420 | 1.163 | -1.099 | -0.047 | -2.142 | 0.567 |
| 441 | O(C(C)(C)C)C(=O)CCC(N)CC(O)=O | 0.464 | 0.223 | -2.085 | -0.155 | 0.209 | 1.495 |
| 442 | O(C(C)(C)C)C1CC(NC1)CC(O)=O | 0.155 | 0.001 | -0.011 | -0.186 | 0.290 | 0.553 |
| 443 | OC(=O)CC(N)C(CC)C | -0.492 | -0.828 | -1.555 | -0.115 | 0.586 | -0.164 |
| 444 | OC(=O)CC(N)CC(C)C | -0.506 | -0.657 | -1.413 | -0.059 | -0.567 | 0.088 |
| 445 | S(CCC(N)CC(O)=O)C | -0.458 | -0.487 | -1.108 | -0.052 | -0.409 | 1.064 |
| 446 | OC(=O)CC(N)Cc1ccccc1 | -0.235 | -0.543 | 0.737 | -0.372 | 0.031 | 0.441 |
| 447 | O(C(C)(C)C)CC(N)CC(O)=O | -0.170 | 0.328 | -1.886 | -0.201 | -0.143 | 0.458 |
| 448 | O(C(C)(C)C)C(C(N)CC(O)=O)C | 0.018 | -0.096 | -2.479 | -0.316 | -0.470 | -0.014 |
| 449 | OC(=O)CC(N)Cc1cc(ccc1)C(C)(C)C | 0.771 | -1.052 | -0.320 | -0.613 | 1.141 | 0.699 |
| 450 | OC(=O)CC(N)C(C)C | -0.744 | -0.747 | -1.229 | 0.023 | -0.801 | -0.613 |
| 451 | OC(=O)CC(N)c1ccccc1 | -0.395 | -0.663 | 0.661 | -0.335 | 1.101 | 0.054 |
| 452 | OC(=O)CC(NC(=O)C)c1ccccc1 | 0.128 | -0.403 | 0.080 | -0.399 | 0.162 | 0.070 |
| 453 | OC(=O)CC(N)CNN | -0.804 | 2.091 | -0.782 | -0.127 | -0.630 | 0.030 |
| 454 | OC(=O)CC(N)CCCCN | -0.262 | 0.169 | -1.095 | -0.125 | -2.203 | 1.401 |
| 455 | OC(=O)CC(N)Cc1c2c([nH]c1)cccc2 | 0.256 | -0.392 | 1.455 | -0.522 | -0.333 | 0.128 |
| 456 | S(CC(N)C(O)=O)CCC(N)C(O)=O | 0.238 | 2.583 | -1.556 | -0.210 | -1.292 | 1.603 |
| 457 | OC(=O)C(CC(N)C(O)=O)C(O)=O | -0.302 | 3.956 | -1.805 | -0.152 | -1.510 | -0.285 |
| 458 | FC(CC(N)C(O)N)C(O)=O | -0.525 | 1.937 | -1.372 | -0.163 | -1.363 | -0.436 |
| 459 | FC(CC(N)C(O)=O)C(O)=O | -0.617 | 2.378 | -1.206 | -0.064 | -1.512 | -0.336 |
| 460 | OC(=O)C1CC(N)CCC1 | -0.669 | -0.772 | 0.663 | -0.050 | -0.683 | -0.862 |
| 461 | OC(=O)CC(N)CC(O)=O | -0.693 | 1.627 | -0.994 | 0.107 | -2.183 | 0.062 |
| 462 | OC(=O)CCN | -1.259 | -0.825 | -0.174 | 0.484 | -1.724 | -0.791 |
| 463 | S(O)(=O)CC(N)C(O)=O | -0.830 | 0.705 | -1.173 | 0.238 | -0.792 | -0.627 |
| 464 | S(O)(=O)(=O)CC(N)C(O)=O | -0.732 | 0.999 | -1.591 | -0.074 | 0.237 | -1.000 |
| 465 | s1ccnc1SCC(N)C(O)=O | -0.306 | 0.578 | 0.506 | -0.036 | 0.898 | 0.633 |
| 466 | S(CC(N)C(O)=O)c1ccc(cc1)C | 0.109 | -0.185 | 0.380 | -0.441 | 1.601 | 1.093 |
| 467 | S(=O)(=O)(NC(NCCCC(N)C(O)=O)=[N-])c1ccc(cc1)C | 2.027 | 1.210 | -0.278 | -0.419 | 1.482 | 1.717 |
| 468 | O(CC=C)C(=O)NCCC(N)C(O)=O | 0.202 | 0.870 | -1.357 | -0.179 | 1.089 | 1.896 |
| 469 | OC(=O)C(N)CCN | -0.976 | 0.699 | -0.696 | 0.061 | -0.674 | -0.713 |
| 470 | OC(=O)C1CC1C(N)C(O)=O | -0.613 | 1.378 | 0.546 | 0.020 | -0.479 | -0.795 |
| 471 | OC(=O)C(N)CC(C)(C)C | -0.550 | -0.505 | -1.805 | -0.122 | 0.737 | -0.890 |
| 472 | s1cccc1C(N)C(O)=O | -0.771 | -0.241 | 0.639 | 0.157 | 0.673 | -0.916 |
| 473 | s1cc(cc1)C(N)C(O)=O | -0.760 | -0.353 | 0.655 | 0.185 | 0.271 | -0.862 |
| 474 | S=1=[CH]C(CC=1)C(N)C(O)=O | -0.699 | 0.013 | 0.583 | 0.389 | 1.350 | -1.237 |
| 475 | OC(=O)C(N)C1CC1 | -1.028 | -0.699 | 0.806 | 0.170 | 0.697 | -1.435 |
| 476 | OC(=O)C(N)C#C | -1.218 | -0.190 | -0.551 | 0.294 | 0.143 | -1.356 |
| 477 | OC(=O)C(N)CC#C | -0.998 | -0.357 | -0.656 | 0.114 | 0.561 | -0.729 |
| 478 | OC(=O)CNC1CCNCC1 | -0.506 | 0.669 | 0.877 | -0.113 | -1.022 | 0.248 |
| 479 | O(C)c1cc(OC)ccc1CNCC(O)=O | 0.481 | 0.078 | 0.053 | -0.350 | -0.140 | 1.122 |
| 480 | OC(=O)CNCCN | -0.913 | 0.497 | -0.329 | 0.088 | -1.222 | 0.494 |
| 481 | OC(=O)CNCCCCN | -0.425 | 0.456 | -0.702 | -0.084 | -1.538 | 1.589 |
| 482 | o1cccc1C(=O)NCC(O)=O | -0.466 | 0.422 | 0.795 | 0.197 | 0.570 | 0.139 |
| 483 | OC(=O)c1ccccc1NCC(=O)C | -0.033 | -0.786 | 0.264 | -0.165 | 0.936 | -0.077 |
| 484 | P(O)(O)(=O)CNCC(O)=O | -0.659 | 2.618 | -1.172 | 0.018 | -0.236 | 0.225 |
| 485 | OC(=O)CNCC(O)=O | -0.883 | 1.858 | -0.483 | 0.195 | -0.799 | 0.383 |
| 486 | OC(=O)C(N)C1Cc2c(C1)cccc2 | -0.072 | -0.653 | 1.430 | -0.373 | 1.616 | -0.380 |
| 487 | OC(=O)CC1NCCC1 | -0.864 | -0.740 | 0.992 | 0.084 | -0.397 | -0.680 |
| 488 | OC(=O)CC(N)C | -1.116 | -0.469 | -0.665 | 0.229 | 0.051 | -1.032 |
| 489 | OC(=O)C(N)C1CCCCC1 | -0.486 | -0.541 | 0.406 | -0.162 | 0.925 | -0.621 |
| 490 | o1cccc1C=CC(=O)NCC(O)=O | -0.029 | 0.302 | 0.783 | 0.037 | 0.715 | 1.182 |
| 491 | OC(=O)CNC | -1.227 | -1.119 | -0.396 | 0.464 | -0.084 | -1.272 |
| 492 | S(CC(N)C(O)=O)CC | -0.649 | -0.317 | -1.074 | -0.028 | 0.780 | 0.295 |
| 493 | S(SCC(N)C(O)=O)CC(N)C(O)=O | 0.254 | 2.711 | -1.926 | -0.252 | -0.621 | 1.544 |
| 494 | S(SCC(N)C(O)=O)c1ncccc1[N+](=O)[O-] | 0.590 | 1.255 | -0.219 | 3.079 | 0.432 | 1.015 |
| 495 | S(C(c1ccccc1)(c1ccccc1)c1ccccc1)CC(N)C(O)=O | 2.372 | -0.528 | 0.974 | -0.608 | 1.215 | -0.658 |
| 496 | SCC(NC(OC(C)(C)C)=O)C(O)=O | 0.232 | 0.458 | -2.712 | -0.117 | -0.396 | 0.160 |
| 497 | S(C(c1ccc(OC)cc1)(c1ccccc1)c1ccccc1)CC(N)C(O)=O | 3.016 | -0.693 | 0.847 | -0.470 | 0.893 | -0.428 |
| 498 | S(Cc1ccc(cc1)C)CC(N)C(O)=O | 0.409 | -0.490 | 0.345 | -0.463 | 2.099 | 1.544 |
| 499 | S(C(C)(C)C)CC(N)C(O)=O | -0.265 | -0.454 | -1.987 | -0.208 | 1.252 | 0.059 |
| 500 | S(SCC(NC(=O)c1ccccc1)C(O)=O)CC(NC(=O)c1ccccc1)C(O)=O | 4.067 | 2.860 | 0.998 | 0.390 | -1.093 | 0.028 |
| 501 | S(CC(NC(OCc1ccccc1)=O)C(O)=O)c1ccccc1 | 2.120 | 0.200 | 1.200 | -0.370 | 0.190 | 1.157 |
| 502 | S(CC(NC(=O)C)C(O)=O)c1ccccc1 | 0.442 | 0.214 | 0.116 | -0.485 | 0.937 | 1.138 |
| 503 | S(CC(N)C(O)=O)C | -0.886 | -0.286 | -0.910 | 0.101 | 0.818 | -0.455 |
| 504 | S(SCC(N)C(O)=O)C(C)(C)C | 0.026 | -0.550 | -2.375 | -0.319 | 1.170 | 0.477 |
| 505 | S(CC(N)C(O)=O)CNC(=O)C | -0.133 | 0.794 | -1.419 | -0.047 | -0.430 | 1.197 |
| 506 | S(CC(N)C(O)=O)c1ccccc1 | -0.123 | -0.428 | 0.558 | -0.401 | 1.451 | 0.789 |
| 507 | S(Cc1ccccc1)CC(N)C(O)=O | 0.142 | -0.330 | 0.548 | -0.395 | 2.029 | 1.337 |
| 508 | OC(=O)C(NC(=O)c1ccccc1)CCC(O)=O | 0.726 | 1.926 | 0.068 | -0.231 | 0.204 | 0.954 |
| 509 | O(C(=O)CCC(N)C(O)=O)CC | -0.208 | 0.565 | -1.340 | -0.045 | 0.352 | 1.007 |
| 510 | O(C(=O)CCC(N)C(O)=O)C | -0.471 | 0.479 | -1.101 | 0.091 | 0.271 | 0.533 |
| 511 | O(C(=O)C(N)CCC(O)=O)c1ccccc1 | 0.365 | 0.952 | 0.445 | -0.259 | 1.085 | 1.499 |
| 512 | O(C(C)(C)C)C(=O)C(NC(OC(C)(C)C)=O)CCC(O)=O | 1.815 | 0.663 | -4.006 | -0.259 | 0.947 | 0.649 |
| 513 | O1c2c(ccc(NC(=O)C(N)CCC(O)=O)c2)C(=CC1=O)C | 1.729 | 1.598 | 1.234 | 0.105 | -0.058 | 1.300 |
| 514 | O(C(=O)CCC(N)C(O)=O)CC=C | -0.043 | 0.771 | -1.308 | -0.062 | -0.185 | 1.463 |
| 515 | O(Cc1ccccc1)C(=O)CCC(N)C(O)=O | 0.673 | 0.773 | 0.561 | -0.319 | 1.953 | 1.872 |
| 516 | O(C(C)(C)C)C(=O)CCC(N)C(O)=O | 0.208 | 0.395 | -2.057 | -0.142 | 0.785 | 0.912 |
| 517 | O(C(=O)C(N)CCC(O)=O)CC=C | -0.019 | 1.001 | -1.525 | -0.080 | 0.300 | 1.336 |
| 518 | OC(=O)C(NC(=O)c1ccc(N)cc1)CCC(O)=O | 0.999 | 2.286 | -0.028 | -0.252 | -0.623 | 0.965 |
| 519 | OC(=O)C(NC(=O)c1ccc([N+](=O)[O-])cc1)CCC(O)=O | 1.400 | 2.554 | -0.149 | 3.751 | -0.866 | 1.358 |
| 520 | OC(=O)C(NC(=O)C)CCC(O)=O | -0.124 | 1.943 | -1.806 | -0.020 | -1.604 | 0.402 |
| 521 | O(Cc1ccccc1)C(=O)C(N)CCC(O)=O | 0.679 | 0.925 | 0.364 | -0.297 | 1.224 | 1.724 |
| 522 | OC(=O)C(N)CCC(=O)CC(C)(C)C | 0.276 | 0.391 | -2.042 | -0.242 | 1.429 | 0.925 |
| 523 | O(C(=O)C(N)CCC(O)=O)C | -0.450 | 0.828 | -1.250 | 0.115 | -0.792 | 0.507 |
| 524 | OC(=O)C(N)CCC(=O)CC(c1ccccc1)(c1ccccc1)c1ccccc1 | 3.233 | -0.895 | 1.237 | -0.326 | 1.868 | -0.713 |
| 525 | O1c2c(cccc2)C(NC(=O)CCC(N)C(O)=O)c2c1cccc2 | 2.142 | 0.781 | 2.184 | -0.152 | 0.069 | -0.255 |
| 526 | O(C)c1cc(OC)cc(OC)c1CNC(=O)CCC(N)C(O)=O | 2.101 | 1.017 | -0.460 | -0.254 | 0.185 | 1.130 |
| 527 | OC(=O)C(N)CCC(=O)NC(c1ccccc1)(c1ccccc1)c1ccccc1 | 3.141 | 0.101 | 1.335 | -0.304 | 0.631 | -0.951 |
| 528 | OC(=O)C(NC(=O)C)CCC(=O)N | -0.098 | 1.960 | -1.839 | -0.114 | -1.273 | 0.230 |
| 529 | OC(=O)C(N)Cc1ncn(c1)C | -0.434 | 0.570 | 0.642 | -0.062 | 1.092 | 0.106 |
| 530 | OC(=O)C(N)Cc1ncn(c1)-c1ccc([N+](=O)[O-])cc1[N+](=O)[O-] | 1.743 | 1.761 | 0.928 | 7.056 | -0.561 | 0.789 |
| 531 | S(=O)(=O)(n1cc(nc1)CC(N)C(O)=O)c1ccc(cc1)C | 1.453 | 0.356 | 0.748 | -0.189 | 1.833 | 0.183 |
| 532 | O(Cc1ccccc1)Cn1cncc1CC(N)C(O)=O | 1.226 | 1.008 | 1.392 | -0.031 | 0.014 | 0.711 |
| 533 | OC(=O)C(N)Cc1ncn(c1)Cc1ccccc1 | 0.779 | 0.465 | 1.347 | -0.220 | 1.962 | 0.657 |
| 534 | OC(=O)C(N)Cc1ncn(c1)C(c1ccccc1)(c1ccccc1)c1ccccc1 | 3.388 | -0.174 | 1.907 | -0.113 | 1.595 | -1.364 |
| 535 | OC(=O)C(N)Cc1[nH]cnc1 | -0.657 | 0.865 | 0.919 | -0.017 | -0.264 | -0.226 |
| 536 | O(C)c1cc(cc(OC)c1)C(OC(=O)NC(CC)C(O)=O)(C)C | 2.034 | -0.336 | -0.952 | -0.324 | -0.473 | 0.595 |
| 537 | OC(=O)C(NC(=O)C)CC(C)C | -0.215 | 0.204 | -2.136 | -0.217 | -0.201 | -0.324 |
| 538 | OC(=O)C(NC(=O)c1ccccc1)CC(C)C | 0.644 | 0.206 | -0.275 | -0.466 | -0.008 | 0.360 |
| 539 | O(Cc1ccccc1)C(=O)NC(CC(C)C)C(O)=O | 1.191 | 0.143 | -0.247 | -0.514 | -0.472 | 1.278 |
| 540 | Clc1ccccc1COC(=O)NCCCCC(N)C(O)=O | 1.913 | 0.502 | 0.260 | -0.322 | 2.709 | 2.732 |
| 541 | Brc1ccccc1COC(=O)NCCCCC(N)C(O)=O | 2.019 | 0.324 | 0.106 | -0.340 | 2.793 | 2.853 |
| 542 | OC(=O)C(N)CCCCNC=O | -0.155 | 0.775 | -0.975 | 0.036 | -0.800 | 1.613 |
| 543 | O=C1CC(CC(=O)C1=C(NCCCCC(N)C(O)=O)CC(C)C)(C)C | 2.946 | 0.518 | -1.116 | -0.064 | 0.715 | 1.033 |
| 544 | O(CC=C)C(=O)NCCCCC(N)C(O)=O | 0.779 | 1.042 | -1.501 | -0.185 | 1.042 | 2.741 |
| 545 | O(C(C)(C)C)C(=O)NCCCCC(N)C(O)=O | 1.042 | 0.758 | -2.104 | -0.180 | 0.848 | 1.995 |
| 546 | OC(=O)C(N(CC(O)=O)CC(O)=O)CCCCN | 0.996 | 3.375 | -3.131 | -0.333 | -1.730 | 0.767 |
| 547 | OC(=O)C(N)CCCCNC(=O)c1ccc(N=Nc2ccc(N(C)C)cc2)cc1 | 4.344 | 1.785 | 1.790 | 0.783 | -0.266 | 1.382 |
| 548 | S1CC2NC(=O)NC2C1CCCCC(=O)NCCCCC(N)C(O)=O | 3.267 | 2.039 | 1.432 | 0.189 | -0.685 | 2.256 |
| 549 | FC(F)(F)C(=O)NCCCCC(N)C(O)=O | 0.524 | 1.354 | -1.548 | -0.041 | -1.012 | 1.825 |
| 550 | OC(=O)C(N)CCCNC(N[N+](=O)[O-])=[N-] | 0.310 | 1.416 | -1.500 | 4.656 | 0.039 | 1.325 |
| 551 | S(=O)(=O)(NC(NCCCC(N)C(O)=O)=N)c1c(C)c(c2OC(Cc2c1C)(C)C)C | 4.120 | 0.196 | 0.000 | 0.062 | -0.071 | -0.687 |
| 552 | S(=O)(=O)(NC(NCCCC(N)C(O)=O)=N)c1ccc(cc1)C | 2.084 | 0.833 | -0.413 | -0.359 | -0.147 | 1.338 |
| 553 | S(=O)(=O)(NC(NCCCC(N)C(O)=O)=[N-])c1c(cc(cc1C)C)C | 2.583 | 0.677 | -0.771 | -0.382 | 1.635 | 1.191 |
| 554 | S(=O)(CCC(N)C(O)=O)C | -0.588 | 1.371 | -1.113 | 0.172 | -1.793 | 0.608 |
| 555 | S(=O)(=O)(CCC(N)C(O)=O)C | -0.417 | 0.514 | -1.560 | -0.075 | 0.287 | 0.262 |
| 556 | S(CCC(NC(=O)C)C(O)=O)C | -0.113 | 0.417 | -1.921 | -0.203 | 0.555 | 0.529 |
| 557 | Clc1ccccc1C(N)C(O)=O | -0.428 | -0.308 | 0.315 | -0.342 | 0.980 | -0.566 |
| 558 | Clc1ccc(cc1)C(N)C(O)=O | -0.360 | -0.730 | 0.427 | -0.332 | 1.269 | -0.151 |
| 559 | OC(=O)C(N)C=1CC=CCC=1 | -0.541 | -0.605 | 0.511 | -0.131 | 0.879 | -0.864 |
| 560 | OC(=O)C(N)(c1ccccc1)c1ccccc1 | 0.398 | -0.653 | 0.948 | -0.608 | 0.673 | -0.886 |
| 561 | Fc1ccccc1C(N)C(O)=O | -0.520 | 0.039 | 0.515 | -0.303 | 0.923 | -0.536 |
| 562 | FC(F)(F)c1cc(ccc1)C(N)C(O)=O | -0.008 | -0.022 | 0.133 | -0.269 | -0.345 | -0.023 |
| 563 | FC(F)(F)c1ccc(cc1)C(N)C(O)=O | 0.050 | -0.272 | 0.242 | -0.194 | -0.623 | 0.388 |
| 564 | OC(=O)C(NC(=O)c1cc([N+](=O)[O-])cc([N+](=O)[O-])c1)c1ccccc1 | 2.108 | 1.698 | 0.732 | 8.925 | -0.603 | -0.080 |
| 565 | OC(=O)CNc1ccccc1 | -0.552 | -0.958 | 0.937 | -0.192 | 1.028 | 0.014 |
| 566 | OC(=O)C(N)c1ccccc1 | -0.610 | -0.621 | 0.626 | -0.252 | 0.808 | -0.745 |
| 567 | OC(=O)C1CC=CC1N | -0.886 | -0.791 | 0.656 | 0.087 | -0.067 | -1.735 |
| 568 | OC(=O)C1CC(N)C=C1 | -0.894 | -0.822 | 0.893 | 0.103 | -1.340 | -1.059 |
| 569 | OC(=O)C1(N)CCCCC1 | -0.699 | -0.650 | 0.431 | -0.075 | 0.638 | -1.469 |
| 570 | OC(=O)C1CNC1 | -1.215 | -0.699 | 1.155 | 0.358 | -2.133 | -1.548 |
| 571 | OC(=O)CC(N)c1ccc([N+](=O)[O-])cc1 | 0.099 | 0.358 | 0.337 | 3.394 | -0.693 | 0.727 |
| 572 | OC(=O)C1(N)CCC1 | -1.057 | -0.504 | 0.683 | 0.164 | 0.355 | -1.883 |
| 573 | OC(=O)CC1(N)CCCCC1 | -0.476 | -0.735 | 0.370 | -0.187 | 0.691 | -0.780 |
| 574 | OC(=O)CC1(N)CCCC1 | -0.675 | -0.744 | 0.516 | -0.043 | 0.579 | -1.111 |
| 575 | OC(=O)C1(N)CC1 | -1.250 | -0.378 | 0.831 | 0.292 | 0.222 | -2.127 |
| 576 | OC(=O)C1CCC(N)CC1 | -0.649 | -0.879 | 0.657 | -0.102 | -0.301 | -0.912 |
| 577 | OC(=O)C1(N)CCCC1 | -0.889 | -0.583 | 0.556 | 0.055 | 0.593 | -1.725 |
| 578 | OC(=O)C1CCCCC1(N)C | -0.489 | -0.754 | 0.079 | -0.175 | 0.130 | -1.474 |
| 579 | OC(=O)C1CCCC1(N)C | -0.689 | -0.796 | 0.220 | -0.084 | 0.705 | -1.828 |
| 580 | OC(=O)C1CCCCC1N | -0.667 | -0.951 | 0.464 | -0.101 | 0.415 | -1.385 |
| 581 | OC(=O)C1CCCC1N | -0.875 | -0.713 | 0.635 | -0.012 | 0.512 | -1.614 |
| 582 | OC(=O)C1CCCCCCC1N | -0.264 | -0.969 | 0.107 | -0.178 | 0.132 | -1.188 |
| 583 | OC(=O)C1CCC=CC1N | -0.733 | -0.586 | 0.595 | -0.107 | 0.600 | -1.227 |
| 584 | OC(=O)C1CC=CCC1N | -0.731 | -0.870 | 0.616 | -0.012 | -1.057 | -1.256 |
| 585 | Oc1cc(ccc1O)CC(N)C(O)=O | -0.072 | 0.879 | 0.374 | -0.369 | -0.778 | 0.203 |
| 586 | OC(=O)C(N)CC=C | -0.963 | -0.618 | -0.828 | 0.115 | 0.655 | -0.932 |
| 587 | OC(=O)CC(N)c1cc2c(cc1)cccc2 | 0.282 | -0.454 | 1.410 | -0.497 | 0.557 | 0.257 |
| 588 | OC(=O)CC(N)c1cccnc1 | -0.486 | 0.490 | 0.781 | -0.220 | 0.230 | 0.105 |
| 589 | FC(F)(F)C(N)C(O)=O | -1.051 | 0.595 | -1.299 | 0.183 | -0.378 | -1.603 |
| 590 | s1cc(c2c1cccc2)CC(N)C(O)=O | 0.137 | -0.432 | 1.263 | -0.217 | 1.369 | -0.001 |
| 591 | OC(=O)C(N)Cc1nc2c(cc1)cccc2 | 0.257 | -0.141 | 1.473 | -0.366 | 1.431 | 0.278 |
| 592 | OC(=O)C(N)Cc1cc2c(nc1)cccc2 | 0.256 | 0.089 | 1.523 | -0.388 | 0.879 | 0.478 |
| 593 | OC(=O)C(N)(C)C | -1.131 | -0.639 | -1.224 | 0.271 | 0.005 | -2.430 |
| 594 | OC(=O)C(N)CC | -1.126 | -0.565 | -0.779 | 0.272 | 0.020 | -1.306 |
| 595 | OC(=O)C(N)Cc1nc2c(nc1)cccc2 | 0.194 | 0.713 | 1.574 | -0.248 | 0.567 | 0.414 |
| 596 | o1cccc1CC(N)C(O)=O | -0.644 | -0.322 | 0.783 | 0.086 | 0.927 | -0.298 |
| 597 | s1cc(cc1)CC(N)C(O)=O | -0.540 | -0.590 | 0.667 | 0.032 | 0.709 | -0.158 |
| 598 | S1CC(=CC=C)C(=C1)CC(N)C(O)=O | 0.088 | -0.376 | 0.011 | -0.192 | 0.090 | 0.108 |
| 599 | OC(=O)C(N)Cc1ncc2c(c1)cccc2 | 0.264 | 0.007 | 1.532 | -0.381 | 1.531 | 0.410 |
| 600 | OC(=O)C(N)C(c1ccccc1)c1ccccc1 | 0.616 | -0.677 | 0.819 | -0.646 | 1.095 | -0.595 |
| 601 | OC(=O)C(N)Cc1c2c(ccc1)cccc2 | 0.279 | -0.527 | 1.216 | -0.461 | 1.479 | -0.060 |
| 602 | OC(=O)C(N)Cc1c2c(cc3c1cccc3)cccc2 | 1.092 | -0.874 | 1.782 | -0.446 | 1.189 | -0.686 |
| 603 | OC(=O)C(N)Cn1ncnc1 | -0.680 | 1.565 | 0.851 | 0.286 | -0.636 | -0.261 |
| 604 | OC(=O)C(N)Cn1nccc1 | -0.661 | 0.684 | 0.813 | -0.090 | 0.325 | -0.315 |
| 605 | OC(=O)C(N)Cc1ncccc1 | -0.483 | 0.545 | 0.794 | -0.219 | 1.092 | 0.038 |
| 606 | s1cccc1CC(N)C(O)=O | -0.557 | -0.528 | 0.723 | 0.044 | 0.951 | -0.177 |
| 607 | OC(=O)C(N)Cc1cccnc1 | -0.460 | 0.166 | 0.817 | -0.204 | -0.340 | 0.167 |
| 608 | OC(=O)C(N)Cc1ccncc1 | -0.466 | 0.143 | 0.801 | -0.163 | -0.391 | 0.138 |
| 609 | s1cc(nc1)CC(N)C(O)=O | -0.588 | 0.478 | 0.773 | 0.086 | 0.548 | 0.022 |
| 610 | OC(=O)C(N)CC=Cc1ccccc1 | 0.047 | -0.363 | 0.671 | -0.311 | 1.955 | 0.943 |
| 611 | OC(=O)C(NC(=O)Cc1c2c([nH]c1)cccc2)C | 0.742 | -0.540 | 1.247 | -0.387 | -0.029 | 0.277 |
| 612 | OC(=O)C(N)Cc1cc2c(cc1)cccc2 | 0.325 | -0.679 | 1.441 | -0.489 | 1.576 | 0.343 |
| 613 | O(C(=O)C(NC(C(O)=O)C)CCc1ccccc1)CC | 1.361 | 0.753 | -0.486 | -0.556 | 1.113 | 1.016 |
| 614 | OC(=O)C(N)CC1CCCCC1 | -0.176 | -1.196 | 0.247 | -0.114 | 1.275 | -0.361 |
| 615 | OC(=O)C(N)=C | -1.317 | -1.136 | -0.700 | 0.490 | -0.861 | -2.327 |
| Eigenvalues | / | 77.906 | 24.017 | 9.670 | 6.306 | 5.550 | 4.736 |
| Pencent_explained | / | 50.27 | 15.50 | 6.23 | 4.06 | 3.58 | 3.06 |
| Cumulative pencent_explained | / | 50.27 | 65.77 | 72.00 | 76.06 | 79.64 | 82.70 |
