# Supplementary material for: An Index for Characterization of Natural and Non-Natural Amino Acids for Peptidomimetics
Source: PLoS One. 2013 Jul 23;8(7):e67844. doi: 10.1371/journal.pone.0067844 (PMC3720802; doi:10.1371/journal.pone.0067844)
Supplement: Table S2 — Sequences of BTDs with the observed and predicted activities. (DOC) [file pone.0067844.s005.doc]

**Table S2.** Sequences of BTDs with the observed and predicted activities

| **No.** | **Peptide** | **Experimental pT** | **Predicted pT** | | | |
| --- | --- | --- | --- | --- | --- | --- |
|  |  |  | PLS^a^ | Error^b^ | GA-PLS^c^ | Error^d^ |
| 1 | GV | 1.13 | 1.265 | -0.135 | 1.371 | -0.241 |
| 2 | GL | 1.68 | 1.459 | 0.221 | 1.619 | 0.061 |
| 3 | GI | 1.70 | 1.365 | 0.335 | 1.523 | 0.177 |
| 4 | GP | 1.35 | 1.697 | -0.347 | 1.632 | -0.282 |
| 5 | GF | 1.80 | 1.941 | -0.141 | 1.864 | -0.064 |
| 6 | GW | 1.89 | 2.083 | -0.193 | 2.062 | -0.172 |
| 7 | GY | 1.77 | 1.637 | 0.133 | 1.573 | 0.197 |
| 8 | AV | 1.16 | 1.382 | -0.222 | 1.406 | -0.246 |
| 9 | AL | 1.70 | 1.577 | 0.123 | 1.654 | 0.046 |
| 10 | AF | 1.72 | 2.058 | -0.338 | 1.899 | -0.179 |
| 11 | VG | 1.19 | 1.278 | -0.088 | 1.114 | 0.076 |
| 12 | VA | 1.16 | 1.440 | -0.280 | 1.382 | -0.222 |
| 13 | VV | 1.71 | 1.822 | -0.112 | 1.867 | -0.157 |
| 14 | VL | 2.00 | 2.017 | -0.017 | 2.114 | -0.114 |
| 15 | LG | 1.72 | 1.541 | 0.179 | 1.467 | 0.253 |
| 16 | LA | 1.72 | 1.703 | 0.017 | 1.735 | -0.015 |
| 17 | LL | 2.35 | 2.280 | 0.070 | 2.467 | -0.117 |
| 18 | LF | 2.75 | 2.762 | -0.012 | 2.712 | 0.038 |
| 19 | LW | 3.40 | 2.904 | 0.496 | 2.910 | 0.490 |
| 20 | LY | 2.46 | 2.458 | 0.002 | 2.421 | 0.039 |
| 21 | IG | 1.68 | 1.436 | 0.244 | 1.271 | 0.409 |
| 22 | IA | 1.68 | 1.598 | 0.082 | 1.538 | 0.142 |
| 23 | IV | 2.05 | 1.980 | 0.070 | 2.023 | 0.027 |
| 24 | IL | 2.26 | 2.175 | 0.085 | 2.271 | -0.011 |
| 25 | II | 2.26 | 2.081 | 0.179 | 2.175 | 0.085 |
| 26 | IP | 2.40 | 2.412 | -0.012 | 2.284 | 0.116 |
| 27 | IW | 3.05 | 2.799 | 0.251 | 2.713 | 0.337 |
| 28 | IN | 1.49 | 1.274 | 0.216 | 1.257 | 0.233 |
| 29 | ID | 1.37 | 1.368 | 0.002 | 1.250 | 0.120 |
| 30 | IQ | 1.49 | 1.520 | -0.030 | 1.559 | -0.069 |
| 31 | IE | 1.37 | 1.382 | -0.012 | 1.412 | -0.042 |
| 32 | IK | 1.65 | 2.193 | -0.543 | 2.195 | -0.545 |
| 33 | IS | 1.49 | 1.083 | 0.407 | 1.026 | 0.464 |
| 34 | IT | 1.49 | 1.317 | 0.173 | 1.330 | 0.160 |
| 35 | PA | 1.32 | 1.556 | -0.236 | 1.567 | -0.247 |
| 36 | PL | 2.22 | 2.132 | 0.088 | 2.300 | -0.080 |
| 37 | PI | 2.33 | 2.039 | 0.291 | 2.204 | 0.126 |
| 38 | PY | 1.80 | 2.310 | -0.510 | 2.254 | -0.454 |
| 39 | PF | 2.80 | 2.614 | 0.186 | 2.545 | 0.255 |
| 40 | FG | 1.77 | 1.979 | -0.209 | 1.927 | -0.157 |
| 41 | FL | 2.87 | 2.718 | 0.152 | 2.927 | -0.057 |
| 42 | FP | 2.70 | 2.955 | -0.255 | 2.940 | -0.240 |
| 43 | FF | 3.10 | 3.200 | -0.1001 | 3.172 | -0.072 |
| 44 | FY | 3.13 | 2.896 | 0.234 | 2.881 | 0.249 |
| 45 | WE | 1.56 | 2.023 | -0.463 | 1.941 | -0.381 |
| 46 | WW | 3.60 | 3.439 | 0.161 | 3.242 | 0.358 |
| 47 | YL | 2.40 | 2.544 | -0.144 | 2.624 | -0.224 |
| 48 | SL | 1.49 | 1.490 | 0.000 | 1.560 | -0.070 |

^a^ The predicted pT value by the PLS model, ^b^ The predicted pT error by the PLS model, ^c^ The predicted pT value by the GA-PLS model, ^d^ The predicted pT error by the GA-PLS model
