# Supplementary material for: An Index for Characterization of Natural and Non-Natural Amino Acids for Peptidomimetics
Source: PLoS One. 2013 Jul 23;8(7):e67844. doi: 10.1371/journal.pone.0067844 (PMC3720802; doi:10.1371/journal.pone.0067844)
Supplement: Table S3 — Computationally designed peptidomimetics of BTDs. (DOC) [file pone.0067844.s006.doc]

**Table S3. Computationally designed peptidomimetics of BTDsa**

| **No.** | **Molecule** | **Predicted pT** |
| --- | --- | --- |
| 1 | 206-108 | 9.784 |
| 2 | 206-439 | 9.204 |
| 3 | 206-206 | 9.174 |
| 4 | 206-524 | 8.827 |
| 5 | 206-350 | 8.748 |
| 6 | 206-534 | 8.723 |
| 7 | 125-108 | 8.593 |
| 8 | 541-108 | 8.563 |
| 9 | 206-551 | 8.526 |
| 10 | 540-108 | 8.486 |
| 11 | 206-547 | 8.370 |
| 12 | 206-437 | 8.326 |
| 13 | 206-497 | 8.302 |
| 14 | 182-108 | 8.265 |
| 15 | 206-541 | 8.216 |
| 16 | 206-527 | 8.172 |
| 17 | 206-540 | 8.092 |
| 18 | 206-71 | 8.058 |
| 19 | 125-439 | 8.013 |
| 20 | 206-125 | 8.005 |
| 21 | 541-439 | 7.983 |
| 22 | 125-206 | 7.982 |
| 23 | 541-206 | 7.953 |
| 24 | 219-108 | 7.915 |
| 25 | 540-439 | 7.906 |
| 26 | 205-108 | 7.904 |
| 27 | 540-206 | 7.875 |
| 28 | 544-108 | 7.869 |
| 29 | 498-108 | 7.806 |
| 30 | 206-182 | 7.778 |
| 31 | 206-500 | 7.736 |
| 32 | 507-108 | 7.701 |
| 33 | 182-439 | 7.685 |
| 34 | 182-206 | 7.654 |
| 35 | 125-524 | 7.635 |
| 36 | 541-524 | 7.606 |
| 37 | 51-108 | 7.578 |
| 38 | 125-350 | 7.556 |
| 39 | 217-108 | 7.552 |
| 40 | 125-534 | 7.531 |
| 41 | 540-524 | 7.528 |
| 42 | 541-350 | 7.527 |
| 43 | 541-534 | 7.502 |
| 44 | 206-548 | 7.492 |
| 45 | 183-108 | 7.462 |
| 46 | 540-350 | 7.449 |
| 47 | 540-534 | 7.424 |
| 48 | 206-51 | 7.368 |
| 49 | 219-439 | 7.335 |
| 50 | 125-551 | 7.334 |
| 51 | 205-439 | 7.325 |
| 52 | 182-524 | 7.307 |
| 53 | 541-551 | 7.305 |
| 54 | 219-206 | 7.304 |
| 55 | 205-206 | 7.294 |
| 56 | 544-439 | 7.289 |
| 57 | 216-108 | 7.276 |
| 58 | 544-206 | 7.258 |
| 59 | 182-350 | 7.228 |
| 60 | 540-551 | 7.228 |
| 61 | 498-439 | 7.226 |
| 62 | 182-534 | 7.203 |
| 63 | 498-206 | 7.195 |
| 64 | 125-547 | 7.179 |
| 65 | 430-108 | 7.155 |
| 66 | 541-547 | 7.149 |
| 67 | 125-437 | 7.134 |
| 68 | 507-439 | 7.121 |
| 69 | 125-497 | 7.110 |
| 70 | 541-437 | 7.105 |
| 71 | 507-206 | 7.090 |
| 72 | 541-497 | 7.080 |
| 73 | 540-547 | 7.072 |
| 74 | 206-498 | 7.049 |
| 75 | 548-108 | 7.041 |
| 76 | 540-437 | 7.027 |
| 77 | 125-541 | 7.024 |
| 78 | 366-108 | 7.010 |
| 79 | 182-551 | 7.006 |
| 80 | 540-497 | 7.003 |
| 81 | 51-439 | 6.999 |
| 82 | 541-541 | 6.995 |
| 83 | 125-527 | 6.980 |
| 84 | 217-439 | 6.972 |
| 85 | 51-206 | 6.968 |
| 86 | 219-524 | 6.957 |
| 87 | 541-527 | 6.950 |
| 88 | 205-524 | 6.947 |
| 89 | 217-206 | 6.942 |
| 90 | 540-541 | 6.918 |
| 91 | 544-524 | 6.911 |
| 92 | 125-540 | 6.900 |
| 93 | 183-439 | 6.882 |
| 94 | 219-350 | 6.878 |
| 95 | 540-527 | 6.873 |
| 96 | 541-540 | 6.871 |
| 97 | 205-350 | 6.868 |
| 98 | 125-71 | 6.866 |
| 99 | 219-534 | 6.853 |
| 100 | 183-206 | 6.852 |
| 101 | 182-547 | 6.851 |
| 102 | 498-524 | 6.848 |
| 103 | 214-108 | 6.843 |
| 104 | 205-534 | 6.843 |
| 105 | 541-71 | 6.837 |
| 106 | 544-350 | 6.832 |
| 107 | 206-507 | 6.830 |
| 108 | 125-125 | 6.813 |
| 109 | 544-534 | 6.807 |
| 110 | 182-437 | 6.806 |
| 111 | 540-540 | 6.793 |
| 112 | 541-125 | 6.784 |
| 113 | 182-497 | 6.782 |
| 114 | 498-350 | 6.769 |
| 115 | 540-71 | 6.759 |
| 116 | 498-534 | 6.744 |
| 117 | 507-524 | 6.743 |
| 118 | 540-125 | 6.707 |
| 119 | 182-541 | 6.696 |
| 120 | 216-439 | 6.696 |
| 121 | 216-206 | 6.665 |
| 122 | 507-350 | 6.664 |
| 123 | 219-551 | 6.657 |
| 124 | 182-527 | 6.652 |
| 125 | 205-551 | 6.646 |
| 126 | 507-534 | 6.639 |
| 127 | 51-524 | 6.621 |
| 128 | 544-551 | 6.610 |
| 129 | 217-524 | 6.595 |
| 130 | 125-182 | 6.586 |
| 131 | 430-439 | 6.575 |
| 132 | 182-540 | 6.572 |
| 133 | 541-182 | 6.557 |
| 134 | 498-551 | 6.548 |
| 135 | 430-206 | 6.545 |
| 136 | 125-500 | 6.544 |
| 137 | 51-350 | 6.542 |
| 138 | 182-71 | 6.538 |
| 139 | 51-534 | 6.517 |
| 140 | 217-350 | 6.516 |
| 141 | 541-500 | 6.515 |
| 142 | 183-524 | 6.505 |
| 143 | 219-547 | 6.501 |
| 144 | 205-547 | 6.490 |
| 145 | 217-534 | 6.490 |
| 146 | 182-125 | 6.485 |
| 147 | 540-182 | 6.479 |
| 148 | 548-439 | 6.461 |
| 149 | 219-437 | 6.456 |
| 150 | 544-547 | 6.455 |
| 151 | 205-437 | 6.446 |
| 152 | 507-551 | 6.443 |
| 153 | 540-500 | 6.437 |
| 154 | 219-497 | 6.432 |
| 155 | 548-206 | 6.431 |
| 156 | 366-439 | 6.430 |
| 157 | 183-350 | 6.426 |
| 158 | 205-497 | 6.422 |
| 159 | 544-437 | 6.410 |
| 160 | 183-534 | 6.400 |
| 161 | 366-206 | 6.399 |
| 162 | 498-547 | 6.392 |
| 163 | 544-497 | 6.386 |
| 164 | 498-437 | 6.347 |
| 165 | 219-541 | 6.347 |
| 166 | 205-541 | 6.336 |
| 167 | 206-370 | 6.330 |
| 168 | 498-497 | 6.323 |
| 169 | 51-551 | 6.320 |
| 170 | 216-524 | 6.318 |
| 171 | 219-527 | 6.302 |
| 172 | 544-541 | 6.300 |
| 173 | 125-548 | 6.300 |
| 174 | 217-551 | 6.294 |
| 175 | 205-527 | 6.292 |
| 176 | 507-547 | 6.287 |
| 177 | 541-548 | 6.271 |
| 178 | 214-439 | 6.263 |
| 179 | 182-182 | 6.258 |
| 180 | 544-527 | 6.256 |
| 181 | 507-437 | 6.242 |
| 182 | 216-350 | 6.239 |
| 183 | 498-541 | 6.238 |
| 184 | 214-206 | 6.233 |
| 185 | 219-540 | 6.222 |
| 186 | 507-497 | 6.218 |
| 187 | 182-500 | 6.216 |
| 188 | 216-534 | 6.214 |
| 189 | 205-540 | 6.212 |
| 190 | 183-551 | 6.204 |
| 191 | 430-524 | 6.198 |
| 192 | 540-548 | 6.193 |
| 193 | 498-527 | 6.193 |
| 194 | 219-71 | 6.188 |
| 195 | 205-71 | 6.178 |
| 196 | 125-51 | 6.176 |
| 197 | 544-540 | 6.176 |
| 198 | 51-547 | 6.165 |
| 199 | 454-108 | 6.150 |
| 200 | 541-51 | 6.147 |
| 201 | 544-71 | 6.142 |
| 202 | 217-547 | 6.138 |
| 203 | 219-125 | 6.136 |
| 204 | 507-541 | 6.133 |
| 205 | 205-125 | 6.125 |
| 206 | 51-437 | 6.120 |
| 207 | 430-350 | 6.119 |
| 208 | 498-540 | 6.113 |
| 209 | 51-497 | 6.096 |
| 210 | 217-437 | 6.094 |
| 211 | 430-534 | 6.093 |
| 212 | 544-125 | 6.089 |
| 213 | 507-527 | 6.088 |
| 214 | 548-524 | 6.083 |
| 215 | 498-71 | 6.079 |
| 216 | 540-51 | 6.069 |
| 217 | 217-497 | 6.069 |
| 218 | 366-524 | 6.052 |
| 219 | 183-547 | 6.048 |
| 220 | 498-125 | 6.027 |
| 221 | 216-551 | 6.017 |
| 222 | 51-541 | 6.010 |
| 223 | 507-540 | 6.008 |
| 224 | 548-350 | 6.004 |
| 225 | 183-437 | 6.004 |
| 226 | 217-541 | 5.984 |
| 227 | 183-497 | 5.979 |
| 228 | 548-534 | 5.979 |
| 229 | 507-71 | 5.974 |
| 230 | 366-350 | 5.973 |
| 231 | 182-548 | 5.972 |
| 232 | 51-527 | 5.966 |
| 233 | 206-336 | 5.964 |
| 234 | 366-534 | 5.948 |
| 235 | 217-527 | 5.939 |
| 236 | 507-125 | 5.922 |
| 237 | 219-182 | 5.908 |
| 238 | 205-182 | 5.898 |
| 239 | 430-551 | 5.897 |
| 240 | 183-541 | 5.894 |
| 241 | 51-540 | 5.886 |
| 242 | 214-524 | 5.886 |
| 243 | 219-500 | 5.866 |
| 244 | 206-298 | 5.866 |
| 245 | 544-182 | 5.862 |
| 246 | 216-547 | 5.862 |
| 247 | 217-540 | 5.860 |
| 248 | 125-498 | 5.857 |
| 249 | 205-500 | 5.856 |
| 250 | 440-108 | 5.854 |
| 251 | 51-71 | 5.852 |
| 252 | 183-527 | 5.849 |
| 253 | 182-51 | 5.848 |
| 254 | 541-498 | 5.828 |
| 255 | 217-71 | 5.826 |
| 256 | 544-500 | 5.820 |
| 257 | 216-437 | 5.817 |
| 258 | 214-350 | 5.806 |
| 259 | 498-182 | 5.799 |
| 260 | 51-125 | 5.799 |
| 261 | 216-497 | 5.793 |
| 262 | 548-551 | 5.783 |
| 263 | 214-534 | 5.781 |
| 264 | 217-125 | 5.773 |
| 265 | 183-540 | 5.770 |
| 266 | 498-500 | 5.757 |
| 267 | 366-551 | 5.751 |
| 268 | 540-498 | 5.750 |
| 269 | 430-547 | 5.741 |
| 270 | 183-71 | 5.736 |
| 271 | 216-541 | 5.707 |
| 272 | 575-108 | 5.707 |
| 273 | 430-437 | 5.697 |
| 274 | 507-182 | 5.694 |
| 275 | 183-125 | 5.683 |
| 276 | 430-497 | 5.672 |
| 277 | 216-527 | 5.663 |
| 278 | 507-500 | 5.652 |
| 279 | 461-108 | 5.647 |
| 280 | 125-507 | 5.638 |
| 281 | 548-547 | 5.627 |
| 282 | 219-548 | 5.622 |
| 283 | 205-548 | 5.612 |
| 284 | 541-507 | 5.608 |
| 285 | 1-108 | 5.603 |
| 286 | 206-300 | 5.602 |
| 287 | 366-547 | 5.596 |
| 288 | 206-75 | 5.591 |
| 289 | 430-541 | 5.587 |
| 290 | 214-551 | 5.585 |
| 291 | 216-540 | 5.583 |
| 292 | 548-437 | 5.582 |
| 293 | 544-548 | 5.576 |
| 294 | 51-182 | 5.572 |
| 295 | 454-439 | 5.570 |
| 296 | 548-497 | 5.558 |
| 297 | 366-437 | 5.551 |
| 298 | 216-71 | 5.549 |
| 299 | 217-182 | 5.546 |
| 300 | 430-527 | 5.542 |
| 301 | 454-206 | 5.539 |
| 302 | 540-507 | 5.531 |
| 303 | 51-500 | 5.530 |
| 304 | 182-498 | 5.529 |
| 305 | 366-497 | 5.527 |
| 306 | 498-548 | 5.513 |
| 307 | 593-108 | 5.511 |
| 308 | 287-108 | 5.507 |
| 309 | 217-500 | 5.504 |
| 310 | 219-51 | 5.498 |
| 311 | 216-125 | 5.496 |
| 312 | 205-51 | 5.488 |
| 313 | 548-541 | 5.473 |
| 314 | 206-546 | 5.466 |
| 315 | 430-540 | 5.463 |
| 316 | 183-182 | 5.456 |
| 317 | 544-51 | 5.452 |
| 318 | 366-541 | 5.441 |
| 319 | 214-547 | 5.429 |
| 320 | 430-71 | 5.429 |
| 321 | 548-527 | 5.428 |
| 322 | 183-500 | 5.414 |
| 323 | 507-548 | 5.408 |
| 324 | 366-527 | 5.397 |
| 325 | 498-51 | 5.389 |
| 326 | 214-437 | 5.384 |
| 327 | 430-125 | 5.376 |
| 328 | 214-497 | 5.360 |
| 329 | 548-540 | 5.349 |
| 330 | 366-540 | 5.317 |
| 331 | 548-71 | 5.315 |
| 332 | 182-507 | 5.310 |
| 333 | 51-548 | 5.286 |
| 334 | 507-51 | 5.284 |
| 335 | 366-71 | 5.283 |
| 336 | 214-541 | 5.275 |
| 337 | 440-439 | 5.275 |
| 338 | 216-182 | 5.269 |
| 339 | 548-125 | 5.262 |
| 340 | 217-548 | 5.260 |
| 341 | 440-206 | 5.244 |
| 342 | 366-125 | 5.230 |
| 343 | 214-527 | 5.230 |
| 344 | 615-108 | 5.228 |
| 345 | 216-500 | 5.227 |
| 346 | 454-524 | 5.192 |
| 347 | 219-498 | 5.179 |
| 348 | 183-548 | 5.170 |
| 349 | 205-498 | 5.169 |
| 350 | 51-51 | 5.162 |
| 351 | 214-540 | 5.151 |
| 352 | 430-182 | 5.149 |
| 353 | 125-370 | 5.138 |
| 354 | 217-51 | 5.136 |
| 355 | 544-498 | 5.133 |
| 356 | 575-439 | 5.127 |
| 357 | 214-71 | 5.117 |
| 358 | 454-350 | 5.113 |
| 359 | 541-370 | 5.108 |
| 360 | 430-500 | 5.107 |
| 361 | 206-1 | 5.103 |
| 362 | 575-206 | 5.097 |
| 363 | 454-534 | 5.088 |
| 364 | 498-498 | 5.070 |
| 365 | 461-439 | 5.067 |
| 366 | 214-125 | 5.064 |
| 367 | 570-108 | 5.051 |
| 368 | 183-51 | 5.046 |
| 369 | 461-206 | 5.036 |
| 370 | 548-182 | 5.035 |
| 371 | 540-370 | 5.031 |
| 372 | 1-439 | 5.023 |
| 373 | 366-182 | 5.003 |
| 374 | 548-500 | 4.993 |
| 375 | 1-206 | 4.992 |
| 376 | 216-548 | 4.983 |
| 377 | 507-498 | 4.965 |
| 378 | 366-500 | 4.961 |
| 379 | 219-507 | 4.960 |
| 380 | 206-615 | 4.952 |
| 381 | 205-507 | 4.950 |
| 382 | 206-454 | 4.947 |
| 383 | 206-287 | 4.938 |
| 384 | 593-439 | 4.931 |
| 385 | 287-439 | 4.927 |
| 386 | 134-108 | 4.925 |
| 387 | 544-507 | 4.914 |
| 388 | 108-108 | 4.910 |
| 389 | 593-206 | 4.901 |
| 390 | 440-524 | 4.897 |
| 391 | 287-206 | 4.896 |
| 392 | 454-551 | 4.892 |
| 393 | 430-548 | 4.863 |
| 394 | 216-51 | 4.859 |
| 395 | 498-507 | 4.851 |
| 396 | 51-498 | 4.843 |
| 397 | 214-182 | 4.837 |
| 398 | 206-10 | 4.835 |
| 399 | 440-350 | 4.818 |
| 400 | 217-498 | 4.816 |
| 401 | 182-370 | 4.810 |
| 402 | 214-500 | 4.795 |
| 403 | 440-534 | 4.793 |
| 404 | 125-336 | 4.772 |
| 405 | 575-524 | 4.750 |
| 406 | 548-548 | 4.748 |
| 407 | 507-507 | 4.746 |
| 408 | 541-336 | 4.743 |
| 409 | 430-51 | 4.739 |
| 410 | 454-547 | 4.736 |
| 411 | 183-498 | 4.727 |
| 412 | 206-440 | 4.726 |
| 413 | 366-548 | 4.717 |
| 414 | 454-437 | 4.691 |
| 415 | 461-524 | 4.689 |
| 416 | 125-298 | 4.674 |
| 417 | 575-350 | 4.671 |
| 418 | 454-497 | 4.667 |
| 419 | 540-336 | 4.665 |
| 420 | 615-439 | 4.648 |
| 421 | 575-534 | 4.646 |
| 422 | 1-524 | 4.645 |
| 423 | 541-298 | 4.645 |
| 424 | 206-457 | 4.634 |
| 425 | 548-51 | 4.625 |
| 426 | 51-507 | 4.624 |
| 427 | 615-206 | 4.618 |
| 428 | 461-350 | 4.610 |
| 429 | 217-507 | 4.597 |
| 430 | 440-551 | 4.596 |
| 431 | 366-51 | 4.593 |
| 432 | 461-534 | 4.585 |
| 433 | 454-541 | 4.582 |
| 434 | 540-298 | 4.567 |
| 435 | 1-350 | 4.566 |
| 436 | 593-524 | 4.554 |
| 437 | 214-548 | 4.550 |
| 438 | 287-524 | 4.549 |
| 439 | 1-534 | 4.541 |
| 440 | 216-498 | 4.540 |
| 441 | 454-527 | 4.537 |
| 442 | 183-507 | 4.507 |
| 443 | 206-570 | 4.488 |
| 444 | 593-350 | 4.475 |
| 445 | 570-439 | 4.472 |
| 446 | 287-350 | 4.470 |
| 447 | 206-461 | 4.470 |
| 448 | 219-370 | 4.460 |
| 449 | 454-540 | 4.458 |
| 450 | 205-370 | 4.450 |
| 451 | 593-534 | 4.449 |
| 452 | 575-551 | 4.449 |
| 453 | 287-534 | 4.445 |
| 454 | 182-336 | 4.444 |
| 455 | 570-206 | 4.441 |
| 456 | 440-547 | 4.441 |
| 457 | 214-51 | 4.427 |
| 458 | 454-71 | 4.423 |
| 459 | 430-498 | 4.420 |
| 460 | 544-370 | 4.414 |
| 461 | 125-300 | 4.410 |
| 462 | 125-75 | 4.399 |
| 463 | 440-437 | 4.396 |
| 464 | 461-551 | 4.389 |
| 465 | 541-300 | 4.381 |
| 466 | 440-497 | 4.372 |
| 467 | 454-125 | 4.371 |
| 468 | 541-75 | 4.370 |
| 469 | 498-370 | 4.351 |
| 470 | 182-298 | 4.346 |
| 471 | 134-439 | 4.345 |
| 472 | 1-551 | 4.344 |
| 473 | 108-439 | 4.330 |
| 474 | 216-507 | 4.321 |
| 475 | 134-206 | 4.314 |
| 476 | 548-498 | 4.305 |
| 477 | 540-300 | 4.303 |
| 478 | 108-206 | 4.299 |
| 479 | 575-547 | 4.293 |
| 480 | 540-75 | 4.292 |
| 481 | 440-541 | 4.286 |
| 482 | 366-498 | 4.274 |
| 483 | 125-546 | 4.274 |
| 484 | 615-524 | 4.271 |
| 485 | 593-551 | 4.253 |
| 486 | 575-437 | 4.249 |
| 487 | 287-551 | 4.249 |
| 488 | 507-370 | 4.246 |
| 489 | 541-546 | 4.244 |
| 490 | 440-527 | 4.242 |
| 491 | 206-134 | 4.235 |
| 492 | 461-547 | 4.233 |
| 493 | 575-497 | 4.225 |
| 494 | 430-507 | 4.200 |
| 495 | 615-350 | 4.191 |
| 496 | 1-547 | 4.189 |
| 497 | 461-437 | 4.188 |
| 498 | 540-546 | 4.167 |
| 499 | 615-534 | 4.166 |
| 500 | 461-497 | 4.164 |
| 501 | 440-540 | 4.162 |
| 502 | 1-437 | 4.144 |
| 503 | 454-182 | 4.143 |
| 504 | 575-541 | 4.139 |
| 505 | 440-71 | 4.128 |
| 506 | 51-370 | 4.124 |
| 507 | 1-497 | 4.120 |
| 508 | 214-498 | 4.107 |
| 509 | 454-500 | 4.101 |
| 510 | 217-370 | 4.097 |
| 511 | 593-547 | 4.097 |
| 512 | 575-527 | 4.095 |
| 513 | 219-336 | 4.094 |
| 514 | 570-524 | 4.094 |
| 515 | 287-547 | 4.093 |
| 516 | 548-507 | 4.086 |
| 517 | 205-336 | 4.084 |
| 518 | 182-300 | 4.082 |
| 519 | 461-541 | 4.078 |
| 520 | 440-125 | 4.075 |
| 521 | 182-75 | 4.071 |
| 522 | 366-507 | 4.055 |
| 523 | 593-437 | 4.053 |
| 524 | 287-437 | 4.048 |
| 525 | 544-336 | 4.048 |
| 526 | 1-541 | 4.034 |
| 527 | 461-527 | 4.034 |
| 528 | 593-497 | 4.028 |
| 529 | 287-497 | 4.024 |
| 530 | 570-350 | 4.015 |
| 531 | 575-540 | 4.015 |
| 532 | 183-370 | 4.007 |
| 533 | 219-298 | 3.996 |
| 534 | 570-534 | 3.990 |
| 535 | 1-527 | 3.990 |
| 536 | 205-298 | 3.986 |
| 537 | 498-336 | 3.985 |
| 538 | 575-71 | 3.981 |
| 539 | 615-551 | 3.970 |
| 540 | 134-524 | 3.967 |
| 541 | 461-540 | 3.954 |
| 542 | 108-524 | 3.952 |
| 543 | 544-298 | 3.950 |
| 544 | 182-546 | 3.946 |
| 545 | 593-541 | 3.943 |
| 546 | 287-541 | 3.939 |
| 547 | 575-125 | 3.928 |
| 548 | 461-71 | 3.920 |
| 549 | 125-1 | 3.911 |
| 550 | 1-540 | 3.910 |
| 551 | 593-527 | 3.898 |
| 552 | 287-527 | 3.894 |
| 553 | 214-507 | 3.888 |
| 554 | 134-350 | 3.888 |
| 555 | 498-298 | 3.887 |
| 556 | 541-1 | 3.882 |
| 557 | 507-336 | 3.880 |
| 558 | 1-71 | 3.876 |
| 559 | 108-350 | 3.873 |
| 560 | 461-125 | 3.868 |
| 561 | 134-534 | 3.863 |
| 562 | 454-548 | 3.857 |
| 563 | 440-182 | 3.848 |
| 564 | 108-534 | 3.848 |
| 565 | 1-125 | 3.823 |
| 566 | 216-370 | 3.821 |
| 567 | 593-540 | 3.819 |
| 568 | 287-540 | 3.814 |
| 569 | 615-547 | 3.814 |
| 570 | 440-500 | 3.806 |
| 571 | 540-1 | 3.804 |
| 572 | 570-551 | 3.793 |
| 573 | 593-71 | 3.785 |
| 574 | 507-298 | 3.782 |
| 575 | 287-71 | 3.780 |
| 576 | 615-437 | 3.770 |
| 577 | 125-615 | 3.760 |
| 578 | 51-336 | 3.758 |
| 579 | 125-454 | 3.755 |
| 580 | 125-287 | 3.746 |
| 581 | 615-497 | 3.745 |
| 582 | 454-51 | 3.734 |
| 583 | 219-300 | 3.732 |
| 584 | 593-125 | 3.732 |
| 585 | 217-336 | 3.731 |
| 586 | 541-615 | 3.730 |
| 587 | 287-125 | 3.728 |
| 588 | 541-454 | 3.725 |
| 589 | 205-300 | 3.722 |
| 590 | 219-75 | 3.721 |
| 591 | 541-287 | 3.717 |
| 592 | 205-75 | 3.711 |
| 593 | 575-182 | 3.701 |
| 594 | 430-370 | 3.700 |
| 595 | 544-300 | 3.686 |
| 596 | 544-75 | 3.675 |
| 597 | 134-551 | 3.666 |
| 598 | 51-298 | 3.660 |
| 599 | 615-541 | 3.660 |
| 600 | 575-500 | 3.659 |
| 601 | 540-615 | 3.653 |
| 602 | 108-551 | 3.651 |
| 603 | 540-454 | 3.648 |
| 604 | 125-8 | 3.644 |
| 605 | 183-336 | 3.641 |
| 606 | 461-182 | 3.640 |
| 607 | 540-287 | 3.640 |
| 608 | 570-547 | 3.638 |
| 609 | 217-298 | 3.633 |
| 610 | 498-300 | 3.623 |
| 611 | 615-527 | 3.615 |
| 612 | 541-8 | 3.614 |
| 613 | 498-75 | 3.612 |

^a^ The 46th sample with a highest pT in the training set was used a template to design peptidomimetics of BTDs.
