# Supplementary material for: An Index for Characterization of Natural and Non-Natural Amino Acids for Peptidomimetics
Source: PLoS One. 2013 Jul 23;8(7):e67844. doi: 10.1371/journal.pone.0067844 (PMC3720802; doi:10.1371/journal.pone.0067844)
Supplement: Table S4 — Sequences of ACE inhibitors with the observed and predicted activities. (DOC) [file pone.0067844.s007.doc]

**Table S4. Sequences of ACE inhibitors with the observed and predicted activities**

| **No.** | **Peptide** | **Experimental pIC_50_** | **Predicted pIC_50_** | | | |
| --- | --- | --- | --- | --- | --- | --- |
|  |  |  | **PLS^a^** | **Error^b^** | **GA-PLS^c^** | **Error^d^** |
| 1 | VW | 5.80 | 4.767 | 1.033 | 4.967 | 0.833 |
| 2 | IW | 5.70 | 4.887 | 0.813 | 5.098 | 0.602 |
| 3 | IY | 5.43 | 4.359 | 1.071 | 4.513 | 0.917 |
| 4 | AW | 5.00 | 4.326 | 0.674 | 4.560 | 0.440 |
| 5 | RW | 4.80 | 4.947 | -0.147 | 4.971 | -0.171 |
| 6 | VY | 4.66 | 4.238 | 0.422 | 4.383 | 0.277 |
| 7 | GW | 4.52 | 4.054 | 0.466 | 4.288 | 0.232 |
| 8 | VF | 4.28 | 4.388 | -0.108 | 4.376 | -0.096 |
| 9 | AY | 4.06 | 3.798 | 0.262 | 3.976 | 0.084 |
| 10 | IP | 3.89 | 3.979 | -0.089 | 3.997 | -0.107 |
| 11 | RP | 3.74 | 4.039 | -0.299 | 3.870 | -0.130 |
| 12 | AF | 3.72 | 3.948 | -0.228 | 3.969 | -0.249 |
| 13 | GY | 3.68 | 3.526 | 0.154 | 3.704 | -0.024 |
| 14 | AP | 3.64 | 3.418 | 0.222 | 3.460 | 0.180 |
| 15 | RF | 3.64 | 4.569 | -0.929 | 4.380 | -0.740 |
| 16 | VP | 3.38 | 3.859 | -0.479 | 3.866 | -0.486 |
| 17 | GP | 3.35 | 3.146 | 0.204 | 3.188 | 0.162 |
| 18 | GF | 3.20 | 3.676 | -0.476 | 3.697 | -0.497 |
| 19 | IF | 3.03 | 4.508 | -1.478 | 4.506 | -1.476 |
| 20 | VG | 2.96 | 2.810 | 0.150 | 2.825 | 0.135 |
| 21 | IG | 2.92 | 2.930 | -0.010 | 2.955 | -0.035 |
| 22 | GI | 2.92 | 2.538 | 0.382 | 2.522 | 0.398 |
| 23 | GM | 2.85 | 2.987 | -0.137 | 2.708 | 0.142 |
| 24 | GA | 2.70 | 2.124 | 0.576 | 2.183 | 0.517 |
| 25 | YG | 2.70 | 2.450 | 0.250 | 2.512 | 0.188 |
| 26 | GL | 2.60 | 2.656 | -0.056 | 2.576 | 0.024 |
| 27 | AG | 2.60 | 2.369 | 0.231 | 2.418 | 0.182 |
| 28 | GH | 2.51 | 3.113 | -0.603 | 3.172 | -0.662 |
| 29 | GR | 2.49 | 2.989 | -0.499 | 2.739 | -0.249 |
| 30 | KG | 2.49 | 2.792 | -0.302 | 2.781 | -0.291 |
| 31 | FG | 2.43 | 2.611 | -0.181 | 2.457 | -0.027 |
| 32 | GS | 2.42 | 1.874 | 0.546 | 1.954 | 0.466 |
| 33 | GV | 2.34 | 2.422 | -0.082 | 2.412 | -0.072 |
| 34 | MG | 2.32 | 2.964 | -0.644 | 2.798 | -0.478 |
| 35 | GK | 2.27 | 2.939 | -0.669 | 2.855 | -0.585 |
| 36 | GE | 2.27 | 2.292 | -0.022 | 2.253 | 0.017 |
| 37 | GT | 2.24 | 1.996 | 0.244 | 2.063 | 0.177 |
| 38 | WG | 2.23 | 2.510 | -0.280 | 2.418 | -0.188 |
| 39 | HG | 2.20 | 2.090 | 0.110 | 1.969 | 0.231 |
| 40 | GQ | 2.15 | 2.378 | -0.228 | 2.330 | -0.180 |
| 41 | GG | 2.14 | 2.097 | 0.043 | 2.146 | -0.006 |
| 42 | QG | 2.13 | 2.272 | -0.142 | 2.233 | -0.103 |
| 43 | SG | 2.07 | 1.962 | 0.108 | 2.043 | 0.027 |
| 44 | LG | 2.06 | 2.933 | -0.873 | 2.897 | -0.837 |
| 45 | GD | 2.04 | 2.118 | -0.078 | 2.220 | -0.180 |
| 46 | TG | 2.00 | 2.258 | -0.258 | 2.331 | -0.331 |
| 47 | EG | 2.00 | 2.180 | -0.180 | 2.209 | -0.209 |
| 48 | DG | 1.85 | 2.166 | -0.316 | 2.259 | -0.409 |
| 49 | PG | 1.77 | 2.488 | -0.718 | 2.345 | -0.575 |
| 50 | LA | 3.51 | 2.960 | 0.550 | 2.934 | 0.576 |
| 51 | KA | 3.42 | 2.819 | 0.601 | 2.818 | 0.602 |
| 52 | RA | 3.34 | 3.017 | 0.323 | 2.865 | 0.475 |
| 53 | YA | 3.34 | 2.477 | 0.863 | 2.548 | 0.792 |
| 54 | AA | 3.21 | 2.396 | 0.814 | 2.455 | 0.755 |
| 55 | FR | 3.04 | 3.503 | -0.463 | 3.050 | -0.010 |
| 56 | HL | 2.49 | 2.650 | -0.160 | 2.399 | 0.091 |
| 57 | DA | 2.42 | 2.194 | 0.226 | 2.296 | 0.124 |
| 58 | EA | 2.00 | 2.207 | -0.207 | 2.245 | -0.245 |

^a^ The Predicted pIC_50_ value by the PLS model, ^b^ The predicted pIC_50_ error by the PLS model, ^c^ The predicted pIC_50_ value by the GA-PLS model, ^d^ The predicted pIC_50_ error by the GA-PLS model.
