# Supplementary material for: An Index for Characterization of Natural and Non-Natural Amino Acids for Peptidomimetics
Source: PLoS One. 2013 Jul 23;8(7):e67844. doi: 10.1371/journal.pone.0067844 (PMC3720802; doi:10.1371/journal.pone.0067844)
Supplement: Table S5 — Computationally designed peptidomimetics of ACE inhibitors. (DOC) [file pone.0067844.s008.doc]

**Table S5. Computationally designed peptidomimetics of ACE inhibitors a**

| **No.** | **Molecule** | **Predicted pIC_50_** |
| --- | --- | --- |
| 1 | 512-439 | 8.338 |
| 2 | 512-534 | 8.040 |
| 3 | 512-524 | 8.036 |
| 4 | 512-437 | 7.970 |
| 5 | 512-497 | 7.848 |
| 6 | 512-527 | 7.794 |
| 7 | 245-439 | 7.793 |
| 8 | 108-439 | 7.671 |
| 9 | 512-495 | 7.601 |
| 10 | 512-551 | 7.589 |
| 11 | 245-534 | 7.494 |
| 12 | 245-524 | 7.490 |
| 13 | 512-33 | 7.486 |
| 14 | 512-547 | 7.447 |
| 15 | 245-437 | 7.424 |
| 16 | 108-534 | 7.372 |
| 17 | 108-524 | 7.368 |
| 18 | 298-439 | 7.311 |
| 19 | 245-497 | 7.303 |
| 20 | 108-437 | 7.302 |
| 21 | 512-525 | 7.266 |
| 22 | 245-527 | 7.249 |
| 23 | 546-439 | 7.232 |
| 24 | 512-71 | 7.190 |
| 25 | 108-497 | 7.181 |
| 26 | 370-439 | 7.135 |
| 27 | 108-527 | 7.126 |
| 28 | 512-548 | 7.120 |
| 29 | 300-439 | 7.106 |
| 30 | 512-500 | 7.098 |
| 31 | 512-27 | 7.095 |
| 32 | 512-233 | 7.077 |
| 33 | 245-495 | 7.055 |
| 34 | 245-551 | 7.044 |
| 35 | 298-534 | 7.012 |
| 36 | 298-524 | 7.008 |
| 37 | 336-439 | 6.996 |
| 38 | 512-350 | 6.967 |
| 39 | 71-439 | 6.945 |
| 40 | 298-437 | 6.943 |
| 41 | 245-33 | 6.941 |
| 42 | 546-534 | 6.933 |
| 43 | 108-495 | 6.933 |
| 44 | 546-524 | 6.929 |
| 45 | 108-551 | 6.922 |
| 46 | 245-547 | 6.901 |
| 47 | 546-437 | 6.864 |
| 48 | 370-534 | 6.836 |
| 49 | 370-524 | 6.832 |
| 50 | 298-497 | 6.821 |
| 51 | 108-33 | 6.819 |
| 52 | 300-534 | 6.807 |
| 53 | 300-524 | 6.803 |
| 54 | 108-547 | 6.779 |
| 55 | 298-527 | 6.767 |
| 56 | 370-437 | 6.766 |
| 57 | 546-497 | 6.742 |
| 58 | 300-437 | 6.738 |
| 59 | 27-439 | 6.726 |
| 60 | 245-525 | 6.720 |
| 61 | 512-103 | 6.714 |
| 62 | 525-439 | 6.710 |
| 63 | 336-534 | 6.698 |
| 64 | 336-524 | 6.694 |
| 65 | 546-527 | 6.688 |
| 66 | 512-60 | 6.657 |
| 67 | 71-534 | 6.646 |
| 68 | 370-497 | 6.645 |
| 69 | 245-71 | 6.644 |
| 70 | 71-524 | 6.642 |
| 71 | 336-437 | 6.628 |
| 72 | 512-600 | 6.621 |
| 73 | 300-497 | 6.616 |
| 74 | 103-439 | 6.612 |
| 75 | 108-525 | 6.598 |
| 76 | 370-527 | 6.590 |
| 77 | 71-437 | 6.576 |
| 78 | 245-548 | 6.575 |
| 79 | 298-495 | 6.574 |
| 80 | 298-551 | 6.562 |
| 81 | 300-527 | 6.562 |
| 82 | 245-500 | 6.552 |
| 83 | 245-27 | 6.549 |
| 84 | 512-238 | 6.539 |
| 85 | 245-233 | 6.531 |
| 86 | 108-71 | 6.522 |
| 87 | 512-236 | 6.515 |
| 88 | 512-560 | 6.509 |
| 89 | 336-497 | 6.507 |
| 90 | 546-495 | 6.495 |
| 91 | 546-551 | 6.483 |
| 92 | 512-235 | 6.472 |
| 93 | 298-33 | 6.459 |
| 94 | 71-497 | 6.455 |
| 95 | 108-548 | 6.452 |
| 96 | 336-527 | 6.452 |
| 97 | 108-500 | 6.430 |
| 98 | 27-534 | 6.428 |
| 99 | 108-27 | 6.427 |
| 100 | 27-524 | 6.424 |
| 101 | 245-350 | 6.422 |
| 102 | 298-547 | 6.420 |
| 103 | 525-534 | 6.411 |
| 104 | 108-233 | 6.409 |
| 105 | 525-524 | 6.407 |
| 106 | 71-527 | 6.401 |
| 107 | 370-495 | 6.397 |
| 108 | 370-551 | 6.386 |
| 109 | 546-33 | 6.380 |
| 110 | 512-449 | 6.372 |
| 111 | 300-495 | 6.369 |
| 112 | 27-437 | 6.358 |
| 113 | 300-551 | 6.357 |
| 114 | 75-439 | 6.356 |
| 115 | 512-361 | 6.352 |
| 116 | 525-437 | 6.341 |
| 117 | 546-547 | 6.341 |
| 118 | 457-439 | 6.341 |
| 119 | 103-534 | 6.313 |
| 120 | 103-524 | 6.309 |
| 121 | 108-350 | 6.300 |
| 122 | 370-33 | 6.283 |
| 123 | 336-495 | 6.259 |
| 124 | 300-33 | 6.254 |
| 125 | 336-551 | 6.247 |
| 126 | 103-437 | 6.243 |
| 127 | 370-547 | 6.243 |
| 128 | 298-525 | 6.239 |
| 129 | 27-497 | 6.237 |
| 130 | 525-497 | 6.220 |
| 131 | 300-547 | 6.215 |
| 132 | 71-495 | 6.208 |
| 133 | 71-551 | 6.196 |
| 134 | 27-527 | 6.182 |
| 135 | 245-103 | 6.168 |
| 136 | 525-527 | 6.166 |
| 137 | 298-71 | 6.162 |
| 138 | 546-525 | 6.160 |
| 139 | 336-33 | 6.145 |
| 140 | 103-497 | 6.122 |
| 141 | 245-60 | 6.112 |
| 142 | 336-547 | 6.105 |
| 143 | 298-548 | 6.093 |
| 144 | 71-33 | 6.093 |
| 145 | 546-71 | 6.083 |
| 146 | 245-600 | 6.076 |
| 147 | 298-500 | 6.071 |
| 148 | 298-27 | 6.068 |
| 149 | 103-527 | 6.068 |
| 150 | 370-525 | 6.062 |
| 151 | 75-534 | 6.057 |
| 152 | 71-547 | 6.053 |
| 153 | 75-524 | 6.053 |
| 154 | 298-233 | 6.050 |
| 155 | 108-103 | 6.046 |
| 156 | 457-534 | 6.042 |
| 157 | 457-524 | 6.038 |
| 158 | 300-525 | 6.034 |
| 159 | 546-548 | 6.014 |
| 160 | 512-189 | 6.009 |
| 161 | 245-238 | 5.993 |
| 162 | 546-500 | 5.992 |
| 163 | 108-60 | 5.990 |
| 164 | 27-495 | 5.989 |
| 165 | 546-27 | 5.989 |
| 166 | 75-437 | 5.987 |
| 167 | 370-71 | 5.986 |
| 168 | 27-551 | 5.977 |
| 169 | 525-495 | 5.973 |
| 170 | 457-437 | 5.972 |
| 171 | 546-233 | 5.971 |
| 172 | 245-236 | 5.969 |
| 173 | 245-560 | 5.963 |
| 174 | 525-551 | 5.961 |
| 175 | 300-71 | 5.957 |
| 176 | 108-600 | 5.953 |
| 177 | 298-350 | 5.940 |
| 178 | 245-235 | 5.927 |
| 179 | 336-525 | 5.924 |
| 180 | 370-548 | 5.916 |
| 181 | 370-500 | 5.894 |
| 182 | 370-27 | 5.891 |
| 183 | 300-548 | 5.888 |
| 184 | 27-33 | 5.875 |
| 185 | 103-495 | 5.875 |
| 186 | 370-233 | 5.873 |
| 187 | 71-525 | 5.872 |
| 188 | 108-238 | 5.871 |
| 189 | 75-497 | 5.866 |
| 190 | 300-500 | 5.866 |
| 191 | 103-551 | 5.863 |
| 192 | 300-27 | 5.863 |
| 193 | 546-350 | 5.861 |
| 194 | 525-33 | 5.858 |
| 195 | 457-497 | 5.851 |
| 196 | 336-71 | 5.848 |
| 197 | 108-236 | 5.847 |
| 198 | 300-233 | 5.845 |
| 199 | 108-560 | 5.841 |
| 200 | 27-547 | 5.835 |
| 201 | 245-449 | 5.826 |
| 202 | 525-547 | 5.818 |
| 203 | 75-527 | 5.812 |
| 204 | 245-361 | 5.806 |
| 205 | 108-235 | 5.804 |

^a^ The first sample with a highest pIC_50_ in the training set was used a template to design peptidomimetics of ACE inhibitors.
