# Supplementary material for: An Index for Characterization of Natural and Non-Natural Amino Acids for Peptidomimetics
Source: PLoS One. 2013 Jul 23;8(7):e67844. doi: 10.1371/journal.pone.0067844 (PMC3720802; doi:10.1371/journal.pone.0067844)
Supplement: Table S6 — Predicted activities of BTDs composed of 22 natural amino acids by excluding the 48 training samples using the QSAR model of BTDs. (DOC) [file pone.0067844.s009.doc]

# Table S6. Predicted activities of BTDs composed of 22 natural amino acids by excluding the 48 training samples using the QSAR model of BTDs

| No. | Peptide | Predicted pT |
| --- | --- | --- |
| 1 | MW | 3.542 |
| 2 | RW | 3.538 |
| 3 | MR | 3.500 |
| 4 | RR | 3.496 |
| 5 | FW | 3.370 |
| 6 | MF | 3.344 |
| 7 | RF | 3.341 |
| 8 | OW | 3.340 |
| 9 | FR | 3.328 |
| 10 | MM | 3.318 |
| 11 | RM | 3.314 |
| 12 | OR | 3.298 |
| 13 | KW | 3.204 |
| 14 | WR | 3.200 |
| 15 | KR | 3.162 |
| 16 | FM | 3.146 |
| 17 | OF | 3.142 |
| 18 | OM | 3.116 |
| 19 | MP | 3.112 |
| 20 | RP | 3.109 |
| 21 | ML | 3.099 |
| 22 | RL | 3.096 |
| 23 | YW | 3.067 |
| 24 | MY | 3.053 |
| 25 | RY | 3.050 |
| 26 | WF | 3.044 |
| 27 | YR | 3.025 |
| 28 | MK | 3.024 |
| 29 | RK | 3.020 |
| 30 | WM | 3.018 |
| 31 | KF | 3.006 |
| 32 | MI | 3.004 |
| 33 | RI | 3.000 |
| 34 | KM | 2.980 |
| 35 | OP | 2.910 |
| 36 | OL | 2.897 |
| 37 | HW | 2.876 |
| 38 | YF | 2.869 |
| 39 | LR | 2.868 |
| 40 | FK | 2.852 |
| 41 | MV | 2.851 |
| 42 | OY | 2.851 |
| 43 | RV | 2.848 |
| 44 | YM | 2.843 |
| 45 | HR | 2.834 |
| 46 | FI | 2.831 |
| 47 | OK | 2.822 |
| 48 | WP | 2.812 |
| 49 | OI | 2.802 |
| 50 | WL | 2.799 |
| 51 | KP | 2.774 |
| 52 | KL | 2.761 |
| 53 | WY | 2.753 |
| 54 | PW | 2.743 |
| 55 | QW | 2.729 |
| 56 | WK | 2.724 |
| 57 | KY | 2.715 |
| 58 | MO | 2.708 |
| 59 | MH | 2.706 |
| 60 | RO | 2.705 |
| 61 | WI | 2.704 |
| 62 | RH | 2.703 |
| 63 | PR | 2.701 |
| 64 | QR | 2.687 |
| 65 | LM | 2.686 |
| 66 | KK | 2.686 |
| 67 | FV | 2.679 |
| 68 | EW | 2.678 |
| 69 | HF | 2.678 |
| 70 | IR | 2.671 |
| 71 | KI | 2.665 |
| 72 | HM | 2.652 |
| 73 | OV | 2.650 |
| 74 | YP | 2.637 |
| 75 | ER | 2.636 |
| 76 | YY | 2.578 |
| 77 | VW | 2.557 |
| 78 | WV | 2.552 |
| 79 | YK | 2.549 |
| 80 | FO | 2.536 |
| 81 | FH | 2.534 |
| 82 | QF | 2.531 |
| 83 | YI | 2.529 |
| 84 | PM | 2.519 |
| 85 | IF | 2.516 |
| 86 | VR | 2.515 |
| 87 | KV | 2.513 |
| 88 | OO | 2.506 |
| 89 | QM | 2.505 |
| 90 | OH | 2.505 |
| 91 | IM | 2.489 |
| 92 | CW | 2.485 |
| 93 | MC | 2.483 |
| 94 | LP | 2.480 |
| 95 | EF | 2.480 |
| 96 | RC | 2.480 |
| 97 | EM | 2.454 |
| 98 | HP | 2.446 |
| 99 | CR | 2.443 |
| 100 | HL | 2.433 |
| 101 | MU | 2.417 |
| 102 | RU | 2.414 |
| 103 | WO | 2.408 |
| 104 | WH | 2.407 |
| 105 | UW | 2.394 |
| 106 | LK | 2.392 |
| 107 | MQ | 2.388 |
| 108 | HY | 2.387 |
| 109 | RQ | 2.384 |
| 110 | YV | 2.377 |
| 111 | LI | 2.372 |
| 112 | KO | 2.370 |
| 113 | KH | 2.368 |
| 114 | MA | 2.367 |
| 115 | RA | 2.363 |
| 116 | VF | 2.359 |
| 117 | HK | 2.358 |
| 118 | UR | 2.352 |
| 119 | HI | 2.337 |
| 120 | VM | 2.333 |
| 121 | NW | 2.314 |
| 122 | PP | 2.313 |
| 123 | FC | 2.311 |
| 124 | QP | 2.299 |
| 125 | CF | 2.287 |
| 126 | QL | 2.286 |
| 127 | OC | 2.282 |
| 128 | NR | 2.272 |
| 129 | CM | 2.261 |
| 130 | EP | 2.248 |
| 131 | FU | 2.245 |
| 132 | ME | 2.241 |
| 133 | QY | 2.240 |
| 134 | RE | 2.237 |
| 135 | EL | 2.235 |
| 136 | YO | 2.233 |
| 137 | YH | 2.232 |
| 138 | PK | 2.225 |
| 139 | IY | 2.225 |
| 140 | LV | 2.220 |
| 141 | OU | 2.216 |
| 142 | FQ | 2.215 |
| 143 | QK | 2.211 |
| 144 | UF | 2.196 |
| 145 | FA | 2.194 |
| 146 | QI | 2.191 |
| 147 | EY | 2.189 |
| 148 | OQ | 2.186 |
| 149 | HV | 2.185 |
| 150 | WC | 2.183 |
| 151 | UM | 2.170 |
| 152 | OA | 2.165 |
| 153 | EK | 2.160 |
| 154 | MT | 2.159 |
| 155 | TW | 2.156 |
| 156 | RT | 2.156 |
| 157 | KC | 2.145 |
| 158 | EI | 2.140 |
| 159 | VP | 2.127 |
| 160 | DW | 2.127 |
| 161 | WU | 2.118 |
| 162 | NF | 2.116 |
| 163 | TR | 2.114 |
| 164 | MG | 2.099 |
| 165 | AW | 2.097 |
| 166 | RG | 2.096 |
| 167 | NM | 2.090 |
| 168 | WQ | 2.088 |
| 169 | MN | 2.086 |
| 170 | DR | 2.085 |
| 171 | RN | 2.082 |
| 172 | KU | 2.079 |
| 173 | MD | 2.079 |
| 174 | LO | 2.076 |
| 175 | RD | 2.075 |
| 176 | LH | 2.075 |
| 177 | FE | 2.068 |
| 178 | VY | 2.068 |
| 179 | WA | 2.067 |
| 180 | CP | 2.055 |
| 181 | AR | 2.055 |
| 182 | PV | 2.052 |
| 183 | KQ | 2.049 |
| 184 | CL | 2.042 |
| 185 | HO | 2.042 |
| 186 | HH | 2.040 |
| 187 | VK | 2.039 |
| 188 | OE | 2.039 |
| 189 | QV | 2.039 |
| 190 | KA | 2.029 |
| 191 | GR | 2.020 |
| 192 | VI | 2.019 |
| 193 | YC | 2.008 |
| 194 | SW | 2.003 |
| 195 | CY | 1.996 |
| 196 | EV | 1.988 |
| 197 | FT | 1.987 |
| 198 | CK | 1.967 |
| 199 | UP | 1.964 |
| 200 | SR | 1.961 |
| 201 | TF | 1.958 |
| 202 | OT | 1.957 |
| 203 | UL | 1.951 |
| 204 | CI | 1.947 |
| 205 | YU | 1.943 |
| 206 | TM | 1.932 |
| 207 | DF | 1.929 |
| 208 | FN | 1.913 |
| 209 | YQ | 1.913 |
| 210 | PO | 1.909 |
| 211 | PH | 1.907 |
| 212 | FD | 1.907 |
| 213 | UY | 1.905 |
| 214 | DM | 1.903 |
| 215 | KE | 1.902 |
| 216 | OG | 1.897 |
| 217 | QO | 1.895 |
| 218 | QH | 1.894 |
| 219 | YA | 1.892 |
| 220 | NP | 1.884 |
| 221 | ON | 1.884 |
| 222 | IO | 1.879 |
| 223 | IH | 1.878 |
| 224 | OD | 1.877 |
| 225 | UK | 1.876 |
| 226 | AM | 1.873 |
| 227 | NL | 1.871 |
| 228 | WT | 1.859 |
| 229 | UI | 1.855 |
| 230 | MS | 1.855 |
| 231 | LC | 1.851 |
| 232 | RS | 1.851 |
| 233 | EO | 1.844 |
| 234 | EH | 1.843 |
| 235 | GM | 1.838 |
| 236 | NY | 1.825 |
| 237 | KT | 1.821 |
| 238 | HC | 1.817 |
| 239 | SF | 1.805 |
| 240 | WG | 1.799 |
| 241 | NK | 1.796 |
| 242 | CV | 1.795 |
| 243 | WN | 1.786 |
| 244 | LU | 1.786 |
| 245 | SM | 1.779 |
| 246 | WD | 1.779 |
| 247 | NI | 1.776 |
| 248 | YE | 1.766 |
| 249 | KG | 1.761 |
| 250 | LQ | 1.756 |
| 251 | HU | 1.751 |
| 252 | KN | 1.747 |
| 253 | KD | 1.741 |
| 254 | TP | 1.726 |
| 255 | VO | 1.723 |
| 256 | VH | 1.722 |
| 257 | HQ | 1.721 |
| 258 | TL | 1.713 |
| 259 | UV | 1.703 |
| 260 | HA | 1.700 |
| 261 | DP | 1.697 |
| 262 | YT | 1.684 |
| 263 | PC | 1.684 |
| 264 | DL | 1.684 |
| 265 | FS | 1.683 |
| 266 | QC | 1.670 |
| 267 | TY | 1.667 |
| 268 | AP | 1.667 |
| 269 | IC | 1.655 |
| 270 | OS | 1.653 |
| 271 | CO | 1.651 |
| 272 | CH | 1.650 |
| 273 | TK | 1.638 |
| 274 | DY | 1.638 |
| 275 | YG | 1.624 |
| 276 | NV | 1.624 |
| 277 | EC | 1.619 |
| 278 | PU | 1.618 |
| 279 | TI | 1.618 |
| 280 | YN | 1.611 |
| 281 | LE | 1.609 |
| 282 | DK | 1.609 |
| 283 | AY | 1.608 |
| 284 | QU | 1.605 |
| 285 | YD | 1.604 |
| 286 | IU | 1.589 |
| 287 | DI | 1.588 |
| 288 | PQ | 1.588 |
| 289 | AK | 1.579 |
| 290 | QQ | 1.575 |
| 291 | HE | 1.574 |
| 292 | SP | 1.573 |
| 293 | UO | 1.560 |
| 294 | AI | 1.558 |
| 295 | UH | 1.558 |
| 296 | WS | 1.555 |
| 297 | QA | 1.554 |
| 298 | EU | 1.553 |
| 299 | GK | 1.544 |
| 300 | LT | 1.527 |
| 301 | EQ | 1.524 |
| 302 | KS | 1.517 |
| 303 | SY | 1.514 |
| 304 | EA | 1.503 |
| 305 | VC | 1.498 |
| 306 | HT | 1.493 |
| 307 | SK | 1.485 |
| 308 | NO | 1.480 |
| 309 | NH | 1.479 |
| 310 | TV | 1.466 |
| 311 | SI | 1.465 |
| 312 | LN | 1.454 |
| 313 | LD | 1.447 |
| 314 | PE | 1.441 |
| 315 | DV | 1.436 |
| 316 | VU | 1.433 |
| 317 | HG | 1.433 |
| 318 | QE | 1.428 |
| 319 | CC | 1.427 |
| 320 | HN | 1.419 |
| 321 | HD | 1.413 |
| 322 | VQ | 1.403 |
| 323 | YS | 1.380 |
| 324 | EE | 1.377 |
| 325 | CU | 1.361 |
| 326 | PT | 1.360 |
| 327 | QT | 1.346 |
| 328 | UC | 1.335 |
| 329 | CQ | 1.331 |
| 330 | TO | 1.322 |
| 331 | TH | 1.321 |
| 332 | SV | 1.313 |
| 333 | CA | 1.310 |
| 334 | PG | 1.300 |
| 335 | ET | 1.295 |
| 336 | DO | 1.293 |
| 337 | DH | 1.291 |
| 338 | PN | 1.286 |
| 339 | QG | 1.286 |
| 340 | PD | 1.280 |
| 341 | QN | 1.273 |
| 342 | UU | 1.269 |
| 343 | QD | 1.266 |
| 344 | AO | 1.263 |
| 345 | AH | 1.261 |
| 346 | VE | 1.256 |
| 347 | NC | 1.255 |
| 348 | UQ | 1.239 |
| 349 | EG | 1.235 |
| 350 | GO | 1.228 |
| 351 | GH | 1.226 |
| 352 | LS | 1.223 |
| 353 | EN | 1.222 |
| 354 | UA | 1.218 |
| 355 | ED | 1.215 |
| 356 | NU | 1.190 |
| 357 | HS | 1.188 |
| 358 | CE | 1.184 |
| 359 | VT | 1.174 |
| 360 | SO | 1.169 |
| 361 | SH | 1.168 |
| 362 | NQ | 1.160 |
| 363 | NA | 1.139 |
| 364 | CT | 1.102 |
| 365 | VN | 1.101 |
| 366 | TC | 1.097 |
| 367 | VD | 1.094 |
| 368 | UE | 1.092 |
| 369 | DC | 1.068 |
| 370 | PS | 1.056 |
| 371 | CG | 1.042 |
| 372 | QS | 1.042 |
| 373 | AC | 1.038 |
| 374 | TU | 1.032 |
| 375 | CN | 1.029 |
| 376 | CD | 1.022 |
| 377 | NE | 1.013 |
| 378 | UT | 1.011 |
| 379 | GC | 1.003 |
| 380 | DU | 1.002 |
| 381 | TQ | 1.002 |
| 382 | ES | 0.991 |
| 383 | TA | 0.981 |
| 384 | DQ | 0.972 |
| 385 | AU | 0.972 |
| 386 | DA | 0.951 |
| 387 | UG | 0.951 |
| 388 | SC | 0.944 |
| 389 | AQ | 0.942 |
| 390 | GU | 0.937 |
| 391 | UN | 0.937 |
| 392 | NT | 0.931 |
| 393 | UD | 0.931 |
| 394 | AA | 0.921 |
| 395 | GQ | 0.907 |
| 396 | GA | 0.887 |
| 397 | SU | 0.879 |
| 398 | NG | 0.871 |
| 399 | VS | 0.870 |
| 400 | NN | 0.858 |
| 401 | TE | 0.855 |
| 402 | ND | 0.851 |
| 403 | SQ | 0.849 |
| 404 | SA | 0.828 |
| 405 | DE | 0.825 |
| 406 | CS | 0.798 |
| 407 | AE | 0.795 |
| 408 | TT | 0.773 |
| 409 | GE | 0.760 |
| 410 | DT | 0.744 |
| 411 | AT | 0.714 |
| 412 | TG | 0.713 |
| 413 | US | 0.706 |
| 414 | SE | 0.702 |
| 415 | TN | 0.700 |
| 416 | TD | 0.693 |
| 417 | DG | 0.684 |
| 418 | GT | 0.679 |
| 419 | DN | 0.670 |
| 420 | DD | 0.664 |
| 421 | AG | 0.654 |
| 422 | AN | 0.640 |
| 423 | AD | 0.634 |
| 424 | NS | 0.627 |
| 425 | ST | 0.620 |
| 426 | GG | 0.619 |
| 427 | GN | 0.605 |
| 428 | GD | 0.599 |
| 429 | SG | 0.560 |
| 430 | SN | 0.547 |
| 431 | SD | 0.540 |
| 432 | TS | 0.469 |
| 433 | DS | 0.439 |
| 434 | AS | 0.409 |
| 435 | GS | 0.375 |
| 436 | SS | 0.316 |
