# Supplementary material for: An Index for Characterization of Natural and Non-Natural Amino Acids for Peptidomimetics
Source: PLoS One. 2013 Jul 23;8(7):e67844. doi: 10.1371/journal.pone.0067844 (PMC3720802; doi:10.1371/journal.pone.0067844)
Supplement: Table S7 — Predicted activities of ACE inhibitors composed of 22 natural amino acids by excluding the 58 training samples using the QSAR model for ACE inhibitors. (DOC) [file pone.0067844.s010.doc]

# Table S7. Predicted activities of ACE inhibitors composed of 22 natural amino acids by excluding the 58 training samples using the QSAR model for ACE inhibitors

| No. | Peptide | Predicted pIC_50_ |
| --- | --- | --- |
| 1 | LW | 5.039 |
| 2 | MW | 4.940 |
| 3 | KW | 4.924 |
| 4 | OW | 4.923 |
| 5 | YW | 4.654 |
| 6 | FW | 4.599 |
| 7 | CW | 4.564 |
| 8 | WW | 4.561 |
| 9 | UW | 4.521 |
| 10 | PW | 4.487 |
| 11 | TW | 4.474 |
| 12 | LY | 4.455 |
| 13 | LF | 4.448 |
| 14 | DW | 4.402 |
| 15 | RY | 4.386 |
| 16 | QW | 4.376 |
| 17 | MY | 4.356 |
| 18 | EW | 4.351 |
| 19 | MF | 4.349 |
| 20 | KY | 4.339 |
| 21 | OY | 4.339 |
| 22 | KF | 4.333 |
| 23 | OF | 4.332 |
| 24 | NW | 4.320 |
| 25 | SW | 4.185 |
| 26 | HW | 4.111 |
| 27 | YY | 4.069 |
| 28 | YF | 4.063 |
| 29 | FY | 4.015 |
| 30 | FF | 4.008 |
| 31 | IH | 3.981 |
| 32 | CY | 3.980 |
| 33 | WY | 3.976 |
| 34 | CF | 3.973 |
| 35 | WF | 3.970 |
| 36 | LP | 3.939 |
| 37 | UY | 3.937 |
| 38 | UF | 3.930 |
| 39 | LH | 3.923 |
| 40 | PY | 3.903 |
| 41 | PF | 3.896 |
| 42 | TY | 3.889 |
| 43 | TF | 3.883 |
| 44 | RH | 3.854 |
| 45 | VH | 3.851 |
| 46 | MP | 3.840 |
| 47 | MH | 3.824 |
| 48 | KP | 3.823 |
| 49 | OP | 3.822 |
| 50 | DY | 3.817 |
| 51 | DF | 3.811 |
| 52 | KH | 3.807 |
| 53 | OH | 3.807 |
| 54 | QY | 3.791 |
| 55 | QF | 3.784 |
| 56 | EY | 3.766 |
| 57 | EF | 3.760 |
| 58 | IO | 3.758 |
| 59 | NY | 3.735 |
| 60 | NF | 3.729 |
| 61 | LO | 3.700 |
| 62 | IK | 3.664 |
| 63 | RO | 3.631 |
| 64 | VO | 3.627 |
| 65 | LK | 3.606 |
| 66 | MO | 3.601 |
| 67 | SY | 3.600 |
| 68 | SF | 3.594 |
| 69 | KO | 3.584 |
| 70 | OO | 3.583 |
| 71 | YP | 3.553 |
| 72 | IR | 3.548 |
| 73 | YH | 3.538 |
| 74 | RK | 3.537 |
| 75 | VK | 3.534 |
| 76 | HY | 3.527 |
| 77 | HF | 3.520 |
| 78 | IM | 3.517 |
| 79 | MK | 3.507 |
| 80 | FP | 3.499 |
| 81 | KK | 3.490 |
| 82 | LR | 3.490 |
| 83 | OK | 3.489 |
| 84 | FH | 3.483 |
| 85 | CP | 3.464 |
| 86 | WP | 3.460 |
| 87 | LM | 3.459 |
| 88 | CH | 3.448 |
| 89 | WH | 3.444 |
| 90 | AH | 3.444 |
| 91 | RR | 3.422 |
| 92 | UP | 3.421 |
| 93 | VR | 3.418 |
| 94 | UH | 3.405 |
| 95 | MR | 3.391 |
| 96 | RM | 3.390 |
| 97 | PP | 3.387 |
| 98 | VM | 3.386 |
| 99 | IL | 3.385 |
| 100 | KR | 3.374 |
| 101 | OR | 3.374 |
| 102 | TP | 3.373 |
| 103 | PH | 3.371 |
| 104 | MM | 3.360 |
| 105 | TH | 3.357 |
| 106 | KM | 3.343 |
| 107 | OM | 3.342 |
| 108 | II | 3.331 |
| 109 | LL | 3.327 |
| 110 | YO | 3.314 |
| 111 | DP | 3.301 |
| 112 | DH | 3.285 |
| 113 | QP | 3.275 |
| 114 | LI | 3.273 |
| 115 | FO | 3.260 |
| 116 | QH | 3.259 |
| 117 | RL | 3.259 |
| 118 | VL | 3.255 |
| 119 | EP | 3.250 |
| 120 | EH | 3.235 |
| 121 | ML | 3.228 |
| 122 | CO | 3.225 |
| 123 | WO | 3.221 |
| 124 | AO | 3.221 |
| 125 | IV | 3.221 |
| 126 | YK | 3.220 |
| 127 | NP | 3.219 |
| 128 | KL | 3.212 |
| 129 | OL | 3.211 |
| 130 | RI | 3.205 |
| 131 | NH | 3.203 |
| 132 | VI | 3.201 |
| 133 | UO | 3.182 |
| 134 | MI | 3.174 |
| 135 | FK | 3.166 |
| 136 | LV | 3.163 |
| 137 | KI | 3.158 |
| 138 | OI | 3.157 |
| 139 | PO | 3.148 |
| 140 | IQ | 3.139 |
| 141 | TO | 3.134 |
| 142 | CK | 3.131 |
| 143 | WK | 3.127 |
| 144 | AK | 3.127 |
| 145 | YR | 3.105 |
| 146 | RV | 3.094 |
| 147 | VV | 3.090 |
| 148 | UK | 3.088 |
| 149 | SP | 3.084 |
| 150 | LQ | 3.081 |
| 151 | YM | 3.073 |
| 152 | SH | 3.069 |
| 153 | MV | 3.064 |
| 154 | IE | 3.062 |
| 155 | DO | 3.062 |
| 156 | PK | 3.054 |
| 157 | KV | 3.047 |
| 158 | OV | 3.046 |
| 159 | TK | 3.040 |
| 160 | QO | 3.036 |
| 161 | ID | 3.029 |
| 162 | FM | 3.018 |
| 163 | CR | 3.015 |
| 164 | RQ | 3.013 |
| 165 | EO | 3.011 |
| 166 | WR | 3.011 |
| 167 | AR | 3.011 |
| 168 | HP | 3.010 |
| 169 | VQ | 3.009 |
| 170 | LE | 3.004 |
| 171 | HH | 2.995 |
| 172 | IA | 2.992 |
| 173 | CM | 2.984 |
| 174 | IN | 2.983 |
| 175 | MQ | 2.982 |
| 176 | NO | 2.980 |
| 177 | WM | 2.980 |
| 178 | AM | 2.980 |
| 179 | UR | 2.972 |
| 180 | LD | 2.971 |
| 181 | DK | 2.968 |
| 182 | KQ | 2.966 |
| 183 | OQ | 2.965 |
| 184 | GO | 2.949 |
| 185 | QK | 2.942 |
| 186 | YL | 2.942 |
| 187 | UM | 2.940 |
| 188 | PR | 2.938 |
| 189 | RE | 2.936 |
| 190 | VE | 2.932 |
| 191 | LN | 2.925 |
| 192 | IC | 2.925 |
| 193 | TR | 2.925 |
| 194 | EK | 2.917 |
| 195 | PM | 2.907 |
| 196 | ME | 2.905 |
| 197 | RD | 2.903 |
| 198 | VD | 2.899 |
| 199 | TM | 2.893 |
| 200 | KE | 2.888 |
| 201 | YI | 2.888 |
| 202 | OE | 2.888 |
| 203 | FL | 2.887 |
| 204 | NK | 2.886 |
| 205 | MD | 2.872 |
| 206 | IT | 2.872 |
| 207 | LC | 2.867 |
| 208 | VA | 2.862 |
| 209 | RN | 2.857 |
| 210 | KD | 2.856 |
| 211 | OD | 2.855 |
| 212 | VN | 2.853 |
| 213 | DR | 2.852 |
| 214 | CL | 2.852 |
| 215 | WL | 2.848 |
| 216 | AL | 2.848 |
| 217 | SO | 2.845 |
| 218 | MA | 2.835 |
| 219 | FI | 2.833 |
| 220 | RG | 2.828 |
| 221 | QR | 2.826 |
| 222 | MN | 2.826 |
| 223 | DM | 2.821 |
| 224 | OA | 2.818 |
| 225 | LT | 2.814 |
| 226 | KN | 2.810 |
| 227 | UL | 2.809 |
| 228 | ON | 2.809 |
| 229 | ER | 2.802 |
| 230 | RC | 2.798 |
| 231 | CI | 2.798 |
| 232 | QM | 2.795 |
| 233 | VC | 2.794 |
| 234 | WI | 2.794 |
| 235 | AI | 2.794 |
| 236 | OG | 2.781 |
| 237 | YV | 2.777 |
| 238 | PL | 2.775 |
| 239 | HO | 2.772 |
| 240 | EM | 2.770 |
| 241 | NR | 2.770 |
| 242 | MC | 2.768 |
| 243 | IS | 2.763 |
| 244 | TL | 2.762 |
| 245 | UI | 2.755 |
| 246 | SK | 2.751 |
| 247 | KC | 2.751 |
| 248 | OC | 2.750 |
| 249 | RT | 2.745 |
| 250 | VT | 2.741 |
| 251 | NM | 2.739 |
| 252 | FV | 2.723 |
| 253 | IU | 2.722 |
| 254 | PI | 2.721 |
| 255 | MT | 2.715 |
| 256 | TI | 2.708 |
| 257 | LS | 2.705 |
| 258 | KT | 2.698 |
| 259 | OT | 2.697 |
| 260 | YQ | 2.696 |
| 261 | DL | 2.689 |
| 262 | CV | 2.688 |
| 263 | WV | 2.684 |
| 264 | AV | 2.684 |
| 265 | HK | 2.678 |
| 266 | LU | 2.664 |
| 267 | QL | 2.663 |
| 268 | UV | 2.645 |
| 269 | FQ | 2.641 |
| 270 | EL | 2.639 |
| 271 | RS | 2.637 |
| 272 | SR | 2.636 |
| 273 | DI | 2.635 |
| 274 | VS | 2.633 |
| 275 | YE | 2.619 |
| 276 | PV | 2.611 |
| 277 | QI | 2.609 |
| 278 | NL | 2.607 |
| 279 | CQ | 2.606 |
| 280 | MS | 2.606 |
| 281 | SM | 2.604 |
| 282 | WQ | 2.603 |
| 283 | AQ | 2.602 |
| 284 | TV | 2.597 |
| 285 | RU | 2.596 |
| 286 | VU | 2.592 |
| 287 | KS | 2.589 |
| 288 | OS | 2.589 |
| 289 | YD | 2.586 |
| 290 | EI | 2.585 |
| 291 | MU | 2.565 |
| 292 | FE | 2.564 |
| 293 | UQ | 2.563 |
| 294 | HR | 2.562 |
| 295 | NI | 2.553 |
| 296 | KU | 2.549 |
| 297 | OU | 2.548 |
| 298 | YN | 2.540 |
| 299 | FD | 2.531 |
| 300 | HM | 2.530 |
| 301 | PQ | 2.529 |
| 302 | CE | 2.529 |
| 303 | WE | 2.525 |
| 304 | AE | 2.525 |
| 305 | DV | 2.525 |
| 306 | TQ | 2.516 |
| 307 | QV | 2.499 |
| 308 | CD | 2.496 |
| 309 | FA | 2.494 |
| 310 | WD | 2.492 |
| 311 | AD | 2.492 |
| 312 | UE | 2.486 |
| 313 | FN | 2.485 |
| 314 | YC | 2.481 |
| 315 | EV | 2.474 |
| 316 | SL | 2.473 |
| 317 | CA | 2.459 |
| 318 | WA | 2.455 |
| 319 | UD | 2.453 |
| 320 | PE | 2.452 |
| 321 | CN | 2.450 |
| 322 | WN | 2.446 |
| 323 | AN | 2.446 |
| 324 | DQ | 2.444 |
| 325 | NV | 2.443 |
| 326 | TE | 2.438 |
| 327 | YT | 2.428 |
| 328 | FC | 2.427 |
| 329 | CG | 2.422 |
| 330 | PD | 2.419 |
| 331 | SI | 2.419 |
| 332 | QQ | 2.417 |
| 333 | UA | 2.416 |
| 334 | UN | 2.407 |
| 335 | TD | 2.406 |
| 336 | EQ | 2.393 |
| 337 | CC | 2.392 |
| 338 | WC | 2.388 |
| 339 | AC | 2.388 |
| 340 | PA | 2.382 |
| 341 | UG | 2.379 |
| 342 | FT | 2.374 |
| 343 | PN | 2.373 |
| 344 | TA | 2.368 |
| 345 | DE | 2.366 |
| 346 | NQ | 2.362 |
| 347 | TN | 2.360 |
| 348 | UC | 2.349 |
| 349 | HI | 2.345 |
| 350 | QE | 2.340 |
| 351 | CT | 2.339 |
| 352 | WT | 2.335 |
| 353 | AT | 2.335 |
| 354 | DD | 2.334 |
| 355 | YS | 2.320 |
| 356 | EE | 2.316 |
| 357 | PC | 2.315 |
| 358 | SV | 2.308 |
| 359 | QD | 2.307 |
| 360 | TC | 2.301 |
| 361 | UT | 2.296 |
| 362 | DN | 2.288 |
| 363 | NE | 2.284 |
| 364 | ED | 2.283 |
| 365 | YU | 2.279 |
| 366 | QA | 2.270 |
| 367 | FS | 2.265 |
| 368 | PT | 2.262 |
| 369 | QN | 2.261 |
| 370 | ND | 2.251 |
| 371 | TT | 2.248 |
| 372 | EN | 2.237 |
| 373 | HV | 2.235 |
| 374 | CS | 2.230 |
| 375 | DC | 2.229 |
| 376 | SQ | 2.227 |
| 377 | WS | 2.226 |
| 378 | AS | 2.226 |
| 379 | FU | 2.224 |
| 380 | NA | 2.214 |
| 381 | NN | 2.205 |
| 382 | QC | 2.203 |
| 383 | CU | 2.189 |
| 384 | US | 2.187 |
| 385 | WU | 2.185 |
| 386 | AU | 2.185 |
| 387 | EC | 2.178 |
| 388 | NG | 2.177 |
| 389 | DT | 2.176 |
| 390 | GN | 2.174 |
| 391 | HQ | 2.153 |
| 392 | PS | 2.153 |
| 393 | QT | 2.150 |
| 394 | SE | 2.150 |
| 395 | NC | 2.147 |
| 396 | UU | 2.146 |
| 397 | TS | 2.140 |
| 398 | ET | 2.125 |
| 399 | SD | 2.117 |
| 400 | GC | 2.116 |
| 401 | PU | 2.112 |
| 402 | TU | 2.099 |
| 403 | NT | 2.094 |
| 404 | SA | 2.079 |
| 405 | HE | 2.076 |
| 406 | SN | 2.071 |
| 407 | DS | 2.067 |
| 408 | HD | 2.043 |
| 409 | QS | 2.041 |
| 410 | DU | 2.027 |
| 411 | ES | 2.017 |
| 412 | SC | 2.012 |
| 413 | HA | 2.006 |
| 414 | QU | 2.000 |
| 415 | HN | 1.997 |
| 416 | NS | 1.985 |
| 417 | EU | 1.976 |
| 418 | ST | 1.959 |
| 419 | NU | 1.944 |
| 420 | HC | 1.938 |
| 421 | GU | 1.913 |
| 422 | HT | 1.885 |
| 423 | SS | 1.851 |
| 424 | SU | 1.810 |
| 425 | HS | 1.777 |
| 426 | HU | 1.736 |
