# Supplementary material for: An Index for Characterization of Natural and Non-Natural Amino Acids for Peptidomimetics
Source: PLoS One. 2013 Jul 23;8(7):e67844. doi: 10.1371/journal.pone.0067844 (PMC3720802; doi:10.1371/journal.pone.0067844)
Supplement: Table S8 — Sequences of 20 inorganic-binding peptides with actual group and predicted group. (DOC) [file pone.0067844.s011.doc]

**Table S8. Sequences of 20 inorganic-binding peptides with actual group and predicted group**

| **No.** | **Molecule** | **%binding** | **Actual group** | **Predicted group**^a^ | **Cross validated**  **predicted group** | **Discriminant**  **scores** |
| --- | --- | --- | --- | --- | --- | --- |
| 1 | TNLFGHL | 60 | 2 | 2 | 2 | -0.957 |
| 2 | THADHAK | 64 | 2 | 1 | 1 | 1.732 |
| 3 | DETPHTT | 63 | 2 | 2 | 2 | -0.610 |
| 4 | STPVNKT | 42 | 2 | 2 | 2 | -1.185 |
| 5 | PYGDTDW | 64 | 2 | 2 | 2 | -1.816 |
| 6 | DRAWPIG | 43 | 2 | 2 | 2 | -0.093 |
| 7 | PPTSLNL | 30 | 2 | 2 | 2 | -2.064 |
| 8 | TLKMPLP | 83 | 1 | 1 | 2 | 0.127 |
| 9 | MVSPRPS | 74 | 1 | 1 | 1 | 1.128 |
| 10 | DLQSQSC | 77 | 1 | 1 | 2 | 0.112 |
| 11 | HQNPLPL | 80 | 1 | 1 | 1 | 1.128 |
| 12 | HAALTMQ | 90 | 1 | 1 | 1 | 0.735 |
| 13 | TLTRVGW | 75 | 1 | 1 | 1 | 1.896 |
| 14 | ISNQKHT | 38 | 2 | 2 | 2 | -0.957 |
| 15 | SLQRATP | 66 | 2 | 2 | 2 | -0.610 |
| 16 | PITLQPA | 80 | 1 | 1 | 1 | 1.128 |
| 17 | ETAGQNT | 51 | 2 | 2 | 2 | -2.064 |
| 18 | QPHDSSS | 84 | 1 | 1 | 2 | 0.112 |
| 19 | PMALDPL | 75 | 1 | 1 | 1 | 1.128 |
| 20 | LPDSAPK | 60 | 2 | 1 | 1 | 1.128 |

^a^ 1: strong binder, 2: moderate binder
