# Supplementary material for: An Index for Characterization of Natural and Non-Natural Amino Acids for Peptidomimetics
Source: PLoS One. 2013 Jul 23;8(7):e67844. doi: 10.1371/journal.pone.0067844 (PMC3720802; doi:10.1371/journal.pone.0067844)
Supplement: Table S9 — Computationally designed mica-binding peptidomimetics. (DOC) [file pone.0067844.s012.doc]

**Table S9.** Computationally designed mica-binding peptidomimetics

| **No.** | **Molecule** | **Predicted group** | **Discriminant**  **score** |
| --- | --- | --- | --- |
| 1 | 17-11-17-2-20-108-18 | 1 | 1.000 |
| 2 | 17-11-17-2-20-439-18 | 1 | 1.000 |
| 3 | 17-11-17-2-20-71-18 | 1 | 1.000 |
| 4 | 17-11-17-2-20-547-18 | 1 | 1.000 |
| 5 | 17-11-17-2-20-350-18 | 1 | 1.000 |
| 6 | 17-11-17-2-20-551-18 | 1 | 1.000 |
| 7 | 17-11-17-2-20-500-18 | 1 | 1.000 |
| 8 | 17-11-17-2-20-534-18 | 1 | 1.000 |
| 9 | 17-11-17-2-20-548-18 | 1 | 1.000 |
| 10 | 17-11-17-2-20-524-18 | 1 | 1.000 |
| 11 | 17-11-17-2-20-437-18 | 1 | 1.000 |
| 12 | 17-11-17-2-20-527-18 | 1 | 1.000 |
| 13 | 17-11-17-2-20-497-18 | 1 | 1.000 |
| 14 | 17-11-17-2-20-564-18 | 1 | 1.000 |
| 15 | 17-11-17-2-20-379-18 | 1 | 1.000 |
| 16 | 17-11-17-2-20-530-18 | 1 | 1.000 |
| 17 | 17-11-17-2-20-231-18 | 1 | 1.000 |
| 18 | 17-11-17-2-20-179-18 | 1 | 1.000 |
| 19 | 17-11-17-2-20-180-18 | 1 | 1.000 |
| 20 | 17-11-17-2-20-56-18 | 1 | 1.000 |
| 21 | 17-11-17-2-20-149-18 | 1 | 1.000 |
| 22 | 17-11-17-2-20-58-18 | 1 | 1.000 |
| 23 | 17-11-17-2-20-495-18 | 1 | 1.000 |
| 24 | 17-11-17-2-20-33-18 | 1 | 1.000 |
| 25 | 17-11-17-2-20-361-18 | 1 | 1.000 |
| 26 | 17-11-17-2-20-60-18 | 1 | 1.000 |
| 27 | 17-11-17-2-20-42-18 | 1 | 0.997 |
| 28 | 17-11-17-2-20-233-18 | 1 | 0.976 |
| 29 | 17-11-17-2-20-449-18 | 1 | 0.973 |
| 30 | 17-11-17-2-20-462-18 | 1 | 0.963 |
| 31 | 17-11-17-2-20-491-18 | 1 | 0.960 |
| 32 | 17-11-17-2-20-8-18 | 1 | 0.940 |
| 33 | 17-11-17-2-20-615-18 | 1 | 0.927 |
| 34 | 17-11-17-2-20-1-18 | 1 | 0.925 |
| 35 | 17-11-17-2-20-570-18 | 1 | 0.604 |
| 36 | 17-11-17-2-20-600-18 | 1 | 0.577 |
| 37 | 17-11-17-2-20-16-18 | 1 | 0.514 |
